# Feasibility of SARS-CoV-2 Surveillance Testing Among Children and Childcare Workers at German Day Care Centers: A Nonrandomized Controlled Trial

**DOI:** 10.1001/jamanetworkopen.2021.42057

**Published:** 2022-01-04

**Authors:** Johannes Forster, Andrea Streng, Paul Rudolph, Viktoria Rücker, Julia Wallstabe, Sandra Timme, Franziska Pietsch, Katrin Hartmann, Maike Krauthausen, Julia Schmidt, Timo Ludwig, David Gierszewski, Thomas Jans, Geraldine Engels, Benedikt Weißbrich, Marcel Romanos, Lars Dölken, Peter Heuschmann, Christoph Härtel, Ildikó Gágyor, Marc Thilo Figge, Oliver Kurzai, Johannes Liese

**Affiliations:** 1 Institute for Hygiene and Microbiology, University of Wuerzburg, Wuerzburg, Germany; 2 Department of Pediatrics, University Hospital Wuerzburg, Wuerzburg, Germany; 3 Leibniz Institute for Natural Product Research and Infection Biology, Hans-Knoell-Institute, Jena, Germany; 4 Institute of Clinical Epidemiology and Biometry, University of Wuerzburg, Wuerzburg, Germany; 5 Department of General Practice, University Hospital Wuerzburg, Wuerzburg, Germany; 6 Department of Child and Adolescent Psychiatry, Psychosomatics and Psychotherapy, University Hospital Wuerzburg, Wuerzburg, Germany; 7 Institute for Virology and Immunobiology, University of Wuerzburg, Wuerzburg, Germany; 8 Clinical Trial Center Wuerzburg, University Hospital Wuerzburg, Wuerzburg, Germany

## Abstract

**Importance:**

Closure of day care centers has been implemented globally to contain the COVID-19 pandemic but has negative effects on children’s health and psychosocial well-being.

**Objective:**

To investigate the feasibility of surveillance among children and childcare workers and to model the efficacy of surveillance on viral spread prevention.

**Design, Setting, and Participants:**

This nonrandomized controlled trial was conducted at 9 day care centers in Wuerzburg, Germany, from October 2020 to March 2021. Participants included children attending day care, childcare workers, and household members. Participating day care centers were assigned to different surveillance modules in a nonrandomized feasibility study. A mathematical model for SARS-CoV-2 spread in day care centers was developed to identify optimal surveillance.

**Interventions:**

Modules 1, 2, and 3 involved continuous surveillance of asymptomatic children and childcare workers by SARS-CoV-2 polymerase chain reaction testing of either midturbinate nasal swabs twice weekly (module 1) or once weekly (module 2) or self-sampled saliva samples twice weekly (module 3). Module 4 involved symptom-based, on-demand testing of children, childcare workers, and their household members by oropharyngeal swabs. All participants underwent SARS-CoV-2 antibody status testing before and after the sampling period. Questionnaires on attitudes and perception of the pandemic were administered in weeks 1, 6, and 12. Mathematical modeling was used to estimate SARS-CoV-2 spread in day care centers.

**Main Outcomes and Measures:**

The primary outcomes were acceptance of the respective surveillance protocols (feasibility study) and the estimated number of secondary infections (mathematical modeling).

**Results:**

Of 954 eligible individuals (772 children and 182 childcare workers), 592 (62%), including 442 children (median [IQR] age, 3 [2-4] years; 214 [48.6%] female) and 150 childcare workers (median [IQR] age, 29 [25-44] years; 129 [90.8%] female) participated in the surveillance. In total, 4755 tests for SARS-CoV-2 detected 2 infections (1 childcare worker and 1 adult household member). Acceptance for continuous surveillance was highest for biweekly saliva testing (150 of 221 eligible individuals [67.9%; 95% CI, 61.5%-73.7%]) compared with biweekly (51 of 117 individuals [43.6%; 95% CI, 35.0%-52.6%]) and weekly (44 of 128 individuals [34.4%; 95% CI, 26.7%-43.0%]) midturbinate swabbing (*P* < .001). Dropout rates were higher for midturbinate swabbing (biweekly, 11 of 62 participants [18%]; once weekly, 11 of 55 participants [20%]) than for saliva testing (6 of 156 participants [4%]). Mathematical modeling based on study and literature data identified biweekly testing of at least 50% of children and childcare workers as minimal requirements to limit secondary infections.

**Conclusions and Relevance:**

In this nonrandomized controlled trial, surveillance for SARS-CoV-2 in 9 German day care centers was feasible and well accepted. Mathematical modeling estimated that testing can minimize the spread of SARS-CoV-2 in day care centers. These findings enable setup of surveillance programs to maintain institutional childcare.

**Trial Registration:**

German Registry for Clinical Trials Identifier: DRKS00023721

## Key Points

**Question:**

Is continuous SARS-CoV-2 testing accepted by children, parents, and childcare workers and can it prevent viral spreading in day care centers?

**Findings:**

In this nonrandomized controlled trial, surveillance testing for SARS-CoV-2 among 954 eligible individuals was well accepted by children, parents, and childcare workers if saliva sampling at home was used. Mathematical modeling based on study and literature data identified biweekly testing of at least 50% of children and childcare workers as minimal requirements to limit secondary infections.

**Meaning:**

These findings suggest that SARS-CoV-2 surveillance testing is feasible and allows for continued day care attendance for children during the COVID-19 pandemic.

## Teaser

This nonrandomized controlled trial investigates the feasibility of SARS-CoV-2 surveillance among children and childcare workers at day care centers in Germany and models the efficacy of surveillance for preventing viral spread.

## Introduction

The global COVID-19 pandemic has resulted in substantial public health measures, including closure of schools and day care centers (DCCs).^1^ The latter was mainly based on evidence showing that children are associated with transmission of influenza and that DCC closure can be effective in limiting viral spread.^2,3,4^ In addition, effective nonpharmaceutical interventions, such as medical masks or physical distancing, cannot be applied for preschool children. Despite differences between influenza viruses and SARS-CoV-2,^5^ modeling studies^6,7,8^ have suggested that closure of schools and DCCs might be beneficial in limiting the dissemination of SARS-CoV-2. However, other models^9,10^ support reopening of childcare facilities, suggesting that they have only a minor impact on viral spreading in the population or disproportionate negative effects. Diminished educational, psychosocial, and nutritional opportunities are negative consequences of DCC closures,^11^ especially for children with special educational needs, chronic disease, and poor socioeconomic status.^12^ Closure of childcare facilities also poses a substantial risk of parenting-related exhaustion,^13^ which is a factor associated with increased risk of child abuse. Indeed, violence against children has been addressed as “a hidden crisis of the COVID-19 pandemic” by the World Health Organization.^14^ Closure of DCCs and schools mainly benefits the society at large, because children are mostly mildly affected by SARS-CoV-2 and complications are rare.^15,16^ Up to now, children have had a very low seroprevalence for SARS-CoV-2,^17^ and a longitudinal testing study^18^ revealed no relevant number of hidden cases among children in kindergarten or schools. Prospective contact tracing indicated a very low risk of secondary transmission in DCCs, and childcare exposure was not associated with SARS-CoV-2 infection.^19,20^ Surveillance testing to prevent viral spread in DCCs could offer an alternative to complete closure. Therefore, we conducted a feasibility study addressing acceptance of different surveillance protocols among children, parents, and childcare workers (CCWs) and performed mathematical modeling to identify feasible and effective testing approaches for DCCs.

## Methods

### Feasibility Study Wü-KiTa-CoV

#### Design and Conduct

**Figure 1.**
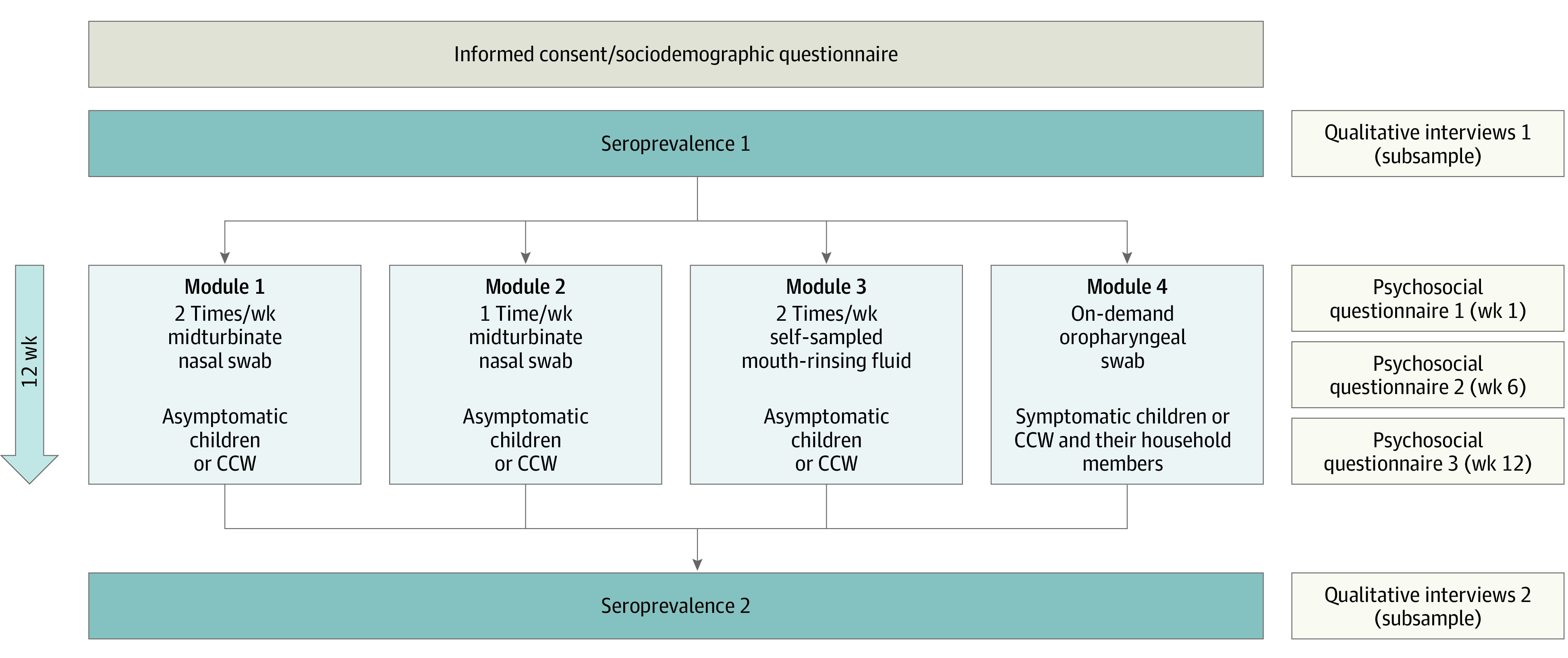
Overview of Study Design Children and childcare workers (CCWs) from day care centers (DCCs) were allocated (per DCC) to 1 of 4 surveillance modules, with respiratory sampling (laboratory analysis by polymerase chain reaction) planned for a period of 12 weeks. Blood sampling (finger prick test) for seroprevalence was conducted before and after the sampling period. Respiratory sampling was accompanied by psychosocial questionnaires at weeks 1, 6, and 12, and qualitative interviews were performed for a representative subsample of participants. As an additional service outside the regular testing schedule, on-demand testing was offered also for symptomatic DCC children and CCWs from modules 1, 2, and 3.

The full protocol of this nonrandomized controlled trial is available in Supplement 1; for an overview of the study design, see Figure 1. The study protocol was approved by the ethics committee of the University Hospital Wuerzburg. The study was conducted according to the Declaration of Helsinki.^21^ Written informed consent was obtained from all CCWs and parents or guardians of the children, with assent from children when appropriate for their age. The study follows the Transparent Reporting of Evaluations With Nonrandomized Designs (TREND) reporting guideline for nonrandomized controlled trials.

In brief, 9 DCCs within the study region of Wuerzburg, Germany, participated in the study from October 2020 to March 2021. Wuerzburg (approximately 130 000 inhabitants) is a city in the north of the German state of Bavaria and is the administrative seat of the district Lower Franconia. Each participating DCC was assigned nonrandomly to 1 of 4 testing approaches (modules 1-4) for 12 weeks. Continuous testing of asymptomatic children and CCWs was done by midturbinate nasal swabbing twice weekly (module 1) or once weekly (module 2) performed by trained test teams in the DCC or by self-sampled mouth-rinsing fluid (ie, saliva testing) sampled by parents or CCWs after initial online video instruction at home twice a week (module 3). In module 4, we instructed children, CCWs, and their household members about SARS-CoV-2–associated symptoms and tested on demand by oropharyngeal swabbing. Parents of children aged 1 year or older and CCWs were asked for informed consent in modules 1, 2, and 4. For module 3, the minimum age was 2 years with regard to the children’s ability to participate in sample acquisition. Before and after the testing period, SARS-CoV-2 antibody testing was performed. SARS-CoV-2 polymerase chain reaction and antibody testing are described in eAppendix 1 in Supplement 2.

#### Questionnaires and Interviews

Before the start of the screening and at weeks 1, 6, and 12, CCWs, children, and their parents answered questionnaires and online surveys on personal data, general attitudes, perception of the pandemic and experience of the specific surveillance method applied. For detailed information see eAppendix 1 in Supplement 2.

#### End Points

The primary end points for modules 1, 2, and 3 were the acceptance rate of the respective surveillance protocol, defined as the proportion of children or CCWs with successful participation (at least 60% of all scheduled samples collected successfully) among all eligible children and CCWs in the DCC estimated via 95% CI according to Wilson score. The primary end point for module 4 is the proportion of cases with successful sampling (ie, completed diagnostic analysis, or receipt of test result in the case of external testing, within 72 hours after first telephone contact with the study center) among all cases of sample collection recommended for symptomatic children, CCWs, or household members, estimated via 95% CI according to Wilson score.

### Statistical Analysis

To determine the module’s association with consent into surveillance, we performed univariable and multivariable logistic regression analysis on the attitude of the parents toward SARS-CoV-2 variables and sociodemographic factors, such as age, sex, and school. Secondary end points included initial consent rates to respiratory surveillance (modules 1-4), dropout rates (modules 1-3), and acceptance of finger-prick blood sampling, stratified by children and CCWs. For details see the study protocol in Supplement 1 and supplemental methods in eAppendix 1 in Supplement 2.

The mathematical model for estimating effectiveness of surveillance testing for prevention of viral spread is described in eAppendix 1 in Supplement 2. Briefly, we set up a DCC-specific state-based model (SBM). Groups consisted of subgroups of children participating or not participating in regular testing. All individuals within the same group could interact with or infect each other. Furthermore, infection transmission was also possible between individuals from different groups and via CCW. To estimate infection spread within the DCC for various scenarios, we simulated each scenario 40 000 times, calculated the average number of secondary infections (ASI), and compared those between different scenarios. A 1-sided permutation test was used to calculate significance, with the threshold set at *P* < .001 (see eAppendix 1 in Supplement 2 for more details). Python pandas version 1.3.4 and Python numpy version 1.21.1 (Python) were used for data analysis.

## Results

### Study Setting

Nine large DCCs (approximately 20% of preschool DCC places in Wuerzburg) were selected for study participation, with 812 registered children and 182 CCWs. Of these, 772 of 812 children (95%) and all CCWs were eligible for the study; 40 children younger than 2 years were excluded in module 3 (eFigure 1A in Supplement 2). Of 954 eligible persons, 592 (62%) participated in the surveillance modules (Table 1), including 442 children (median [IQR] age, 3 [2-4] years; 214 [48.6%] female) and 150 CCWs (median [IQR] age, 29 [25-44] years; 129 [90.8%] female).

**Table 1.** Participants Who Initially Consented to Participate in SARS-CoV-2 Testing at Day Care Centers

Group	Participants, No./total No. of eligible persons (%)				
	Overall	Module 1	Module 2	Module 3	Module 4
Children^a^	442/772 (57)	44/99 (44)	43/104 (41)	121/180 (67)	234/389 (60)
Childcare workers	150/182 (82)	19/19 (100)	14/26 (54)	37/43 (86)	80/94 (85)
Total	592/954 (62)	63/118 (53)	57/130 (44)	158/223 (71)	314/483 (65)

^a^ For children, parents or guardians provided informed consent.

**Figure 2.**
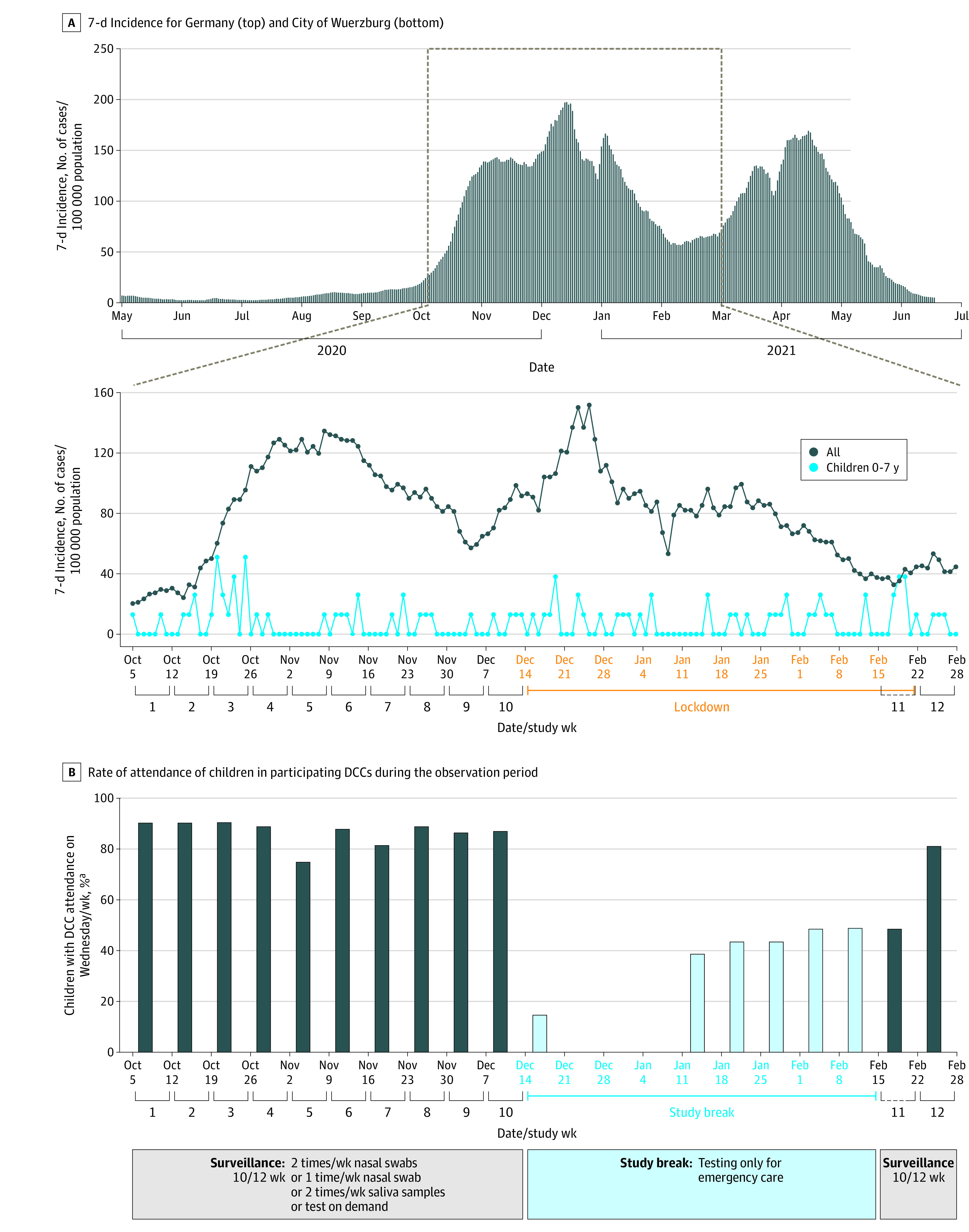
General Overview of the Pandemic Activity in Wuerzburg During the Study Period A, Top panel shows 7-day incidence per week for Germany, and bottom panel shows data for the city of Wuerzburg. B, Graphs shows rate of attendance of children in participating day care centers (DCCs) during the observation period. Data are the average attendance rate on Wednesdays of at least 8 of 9 DCCs.

The study took place from October 2020 to March 2021, before any vaccine availability for study participants, and included 12 weeks of regular respiratory surveillance (Figure 2 and eFigure 2 in Supplement 2). The regional 7-day incidence during the study period was 20 to 152 cases per 100 000 population (Figure 2). As the result of a national lockdown, the study was paused from December 14, 2020, to February 14, 2021.

### Initial Attitudes and Concerns of Parents and CCWs Regarding the Pandemic

Detailed sociodemographic characteristics and information on attitudes regarding the pandemic were available from 570 parents with 635 eligible children (440 participants and 195 nonparticipants in the respiratory surveillance) (eTable 1 in Supplement 2) and from 157 CCWs (150 participants and 7 nonparticipants) (eTable 2 in Supplement 2) at the start of the study. Of all parents, 88% (446 of 505 parents) considered DCC access as fairly or very important in their daily life, and 39% (197 of 509 parents) were personally acquainted with someone affected by COVID-19. With regard to SARS-CoV-2, 33% (166 of 506 parents) considered the virus as fairly or very dangerous to their family, and 72% (366 of 507 parents) regarded it as fairly or very dangerous to society overall. Eighty-four percent (424 of 506 parents) approved restrictive measures to control SARS-CoV-2 as fairly or very important, although 36% (185 of 509 parents) felt fairly or very limited in their personal life. CCWs had similar attitudes and felt similarly limited as parents did by the pandemic and by control measures. There were higher proportions of CCWs vs parents who were strongly affected by the fear of transmitting SARS-CoV-2 infection to others (42% [53 of 127 CCWs] vs 26% [90 of 343 parents]; difference, 16%; 95% CI, 6%-25%; *P* = .001), of getting infected themselves (20% [25 of 127 CCWs] vs 10% [33 of 340 parents]; difference, 10%; 95% CI, 3%-18%; *P* = .003), or of developing a severe course of COVID-19 (20% [25 of 126 CCWs] vs 8% [26 of 340 parents]; difference, 12%; 95% CI, 5%-20%; *P* < .001). Furthermore, 23% of responding CCWs (27 of 119 CCWs) but only 8% of parents (27 of 340 parents) showed values above norm for depression and anxiety (difference, 14%; 95% CI, 7%-23%; *P* < .001).

### Initial Consent for SARS-CoV-2 Testing Among Parents and CCWs

Consent to respiratory surveillance was received for 442 of 772 children (57%) and 150 of 182 CCWs (82%) (Table 1). Initial consent rates were 59% (278 of 471 participants) for continuous testing (modules 1-3) and 65% (314 of 483 participants) for symptomatic testing (module 4). In multivariable logistic regression adjusted for age, sex, education, and attitudes toward SARS-CoV-2, the proposed type of respiratory sampling (module) was a key factor in the parents’ decision to participate or not, with lowest consent for biweekly nasal swabs (odds ratio [OR], 0.11; 95% CI, 0.05-0.27; *P* < . 001). Parents who agreed for their children to participate in testing had a higher educational level (secondary education, OR, 1.9; 95% CI, 0.72-5.05; *P* = .01), felt less restricted by the pandemic in their personal life (OR, 1.78; 95% CI, 1.08-2.94; *P* = .02), considered vaccinations in general as more important (OR, 0.31; 95% CI, 0.15-0.63; *P* = .001), and were more willing to be vaccinated in the future against SARS-CoV-2 (OR, 5.01; 95% CI, 2.25-11.18; *P* < .001) than nonparticipants (eTable 1 and eTable 3 in Supplement 2). Perception of the pandemic as a strong danger to society, personal acquaintance with someone with a confirmed case of SARS-CoV-2, and considering restrictive measures against the pandemic as important were positively associated with the parents’ decision to participate in univariable analysis, but not confirmed by multivariable analysis.

Of 171 parents who rejected participating in the respiratory surveillance, 92 (54%) stated their reasons (eTable 3 in Supplement 2). They mainly feared a negative experience for their children due to respiratory sampling and/or the presence of study personnel (27 of 92 parents [29%]). Only 5 of 171 parents (3%) expressed outright denial of the pandemic.

### Acceptance Rates for Long-term Surveillance

Long-term acceptance (defined as successful participation in at least 60% of all scheduled samples) of continuous respiratory surveillance (modules 1-3) as the main outcome measure was highest for biweekly saliva testing (150 of 221 eligible individuals [67.9%; 95% CI, 61.5%-73.7%]) compared with biweekly (51 of 117 individuals [43.6%; 95% CI, 35.0%-52.6%]) and weekly (44 of 128 individuals [34.4%; 95% CI, 26.7%-43.0%]) midturbinate swabbing (*P* < .001) (Table 2). Similarly, acceptance among participating children was also highest for saliva sampling (117 of 179 children [65.4%]), compared with 33.3% (33 of 99 children) for biweekly nasal swabbing and 31.7% (33 of 104 children) for weekly nasal swabbing. In all modules, CCW acceptance rates were higher than those of children (Table 2). In total, 10% of participants (28 of 273 participants, including 24 children and 4 CCWs) dropped out from continuous surveillance (eTable 4 in Supplement 2). Overall dropout rates were 18% (11 of 62 participants) in module 1, 20% (11 of 55 participants) in module 2, and 4% (6 of 156 participants) in module 3. Dropout rates for children were approximately 10 times higher for midturbinate swabbing (module 1, 25% [11 of 44 participants]; module 2, 23% [10 of 43 participants]) than for saliva testing (module 3, 2.5% [3 of 120 participants]). The main reason for dropout in children was repeated refusal of sampling. For 66 children who successfully completed respiratory surveillance, the median (IQR) time for taking a single nasal swab by medically trained persons was 1.6 (1.4-1.8) minutes) in contrast to a median (IQR) time of 2.5 (2.0-3.0) minutes for 15 children who finally dropped out (*P* < .001; Mann-Whitney *U* statistic = 935.5; *z* = 3.8924). For successful saliva sampling at home, 117 parents reported a median (IQR) time of less than 1 minute (0.95 [0.8-1.2] minutes). In module 4, 179 symptomatic participants (77 children, 25 CCWs, and 77 household members) were scheduled for 220 oropharyngeal swabs (96 children, 35 CCWs, and 89 household members) (eFigure 1B in Supplement 2). Of those, 214 respiratory samples were collected and 199 (94%) tests were successful (defined as result available within 72 hours) (eTable 4 in Supplement 2).

**Table 2.** Acceptance of Continuous Respiratory Surveillance

Group	Module 1^a^		Module 2^b^		Module 3^c^	
	Successful, No. of participants/total No.^d^	Rate (95% CI), %	Successful, No. of participants/total No.^d^	Rate (95% CI), %	Successful, No. of participants/total No.^d^	Rate (95% CI), %
Children	33/99	33.3 (24.8-43.1)	33/104	31.7 (23.3-41.2)	117/179	65.4 (58.1-71.9)
Child care workers	18/18	100.0 (82.4-100.0)	11/24	45.8 (27.9-64.9)	33/42	78.6 (64.1-88.3)
Overall	51/117	43.6 (35.0-52.6)	44/128	34.4 (26.7-43.0)	150/221	67.9 (61.5-73.7)

^a^ Module 1 included SARS-CoV-2 surveillance by biweekly nasal midturbinate swabs over the regular study period of 12 weeks.

^b^ Module 2 included SARS-CoV-2 surveillance by weekly nasal midturbinate swabs over the regular study period of 12 weeks.

^c^ Module 3 included SARS-CoV-2 surveillance by weekly saliva sampling over the regular study period of 12 weeks.

^d^ A participant in the respiratory surveillance was classified as successful if 60% of all planned samples were available from the participant. One child and 4 childcare workers who were initially considered eligible and had given informed consent to respiratory sampling were excluded from primary end point analysis, because they could not participate in respiratory sampling for reasons unrelated to the respiratory surveillance measures (eg, seropositivity for SARS-CoV-2 at the beginning of the study, maternity leave, and change of day care center).

During the study the proportion of parents with indicators of anxiety or depression (Patient Health Questionnaire–4 values above the norm) remained stable in continuous testing (modules 1-3, 10 [9%] vs 13 [11.5%] of 113 parents) but increased with testing on demand (module 4, 7 [5%] vs 18 [13%] of 138 parents; *P* = .008; McNemar test χ^2^_1_ = 7.1). At the end of the study, parents from modules 1 to 3 rated the positive effects of the study measures significantly higher than did parents from module 4 (eFigure 3A in Supplement 2), regarding overall usefulness (fairly or very useful, 123 of 129 parents [95%] vs 114 of 156 parents [73%]; χ^2^_4_ = 61.7; *P* < .001), satisfaction with the implementation (fairly or very satisfied, 123 of 129 parents [95%] vs 129 of 157 parents [82%]; χ^2^_4_ = 42.0; *P* < .001), and sense of security (fairly or very affected, 100 of 129 parents [78%] vs 79 of parents 156 [51%]; χ^2^_4_ = 25.4; *P* < .001), and felt less affected by the study measures regarding themselves (not at all or a little affected, 117 of 130 parents [90%] vs 120 of 157 parents [76%]; χ^2^_4_ = 13.8; *P* = .008), their children (126 of 145 parents [87%] vs 126 of 183 parents [69%]; χ^2^_4_ = 42.2; *P* < .001), families (124 of 130 parents [95%] vs 114 of 156 parents [73%]; χ^2^_3_ = 44.3; *P* < .001), and daily life (124 of 129 parents [96%] vs 117 of 155 parents [75%]; χ^2^_4_ = 24.0; *P* < .001). CCWs from modules 1 to 3 were also more content (eFigure 3B in Supplement 2). The proportion of CCWs with anxiety or depression remained stable in all modules.

### Identification of SARS-CoV-2 Imports Into DCCs

In total, 4755 tests for SARS-CoV-2 were conducted during the regular study period. In DCCs with continuous testing (modules 1-3), no SARS-CoV-2 infection was detected in either asymptomatic children (3442 tests) or CCWs (1099 tests) during the regular 12-week study period. In module 4, 214 oropharyngeal swabs from 179 symptomatic participants were conducted, and SARS-CoV-2 was detected in 2 participants (1 CCW and 1 adult household member). For additional SARS-CoV-2 detections during the lockdown and for test indications other than symptoms, see eAppendix 2 in Supplement 2.

Overall, 72% (559 of 772) of eligible participants initially agreed to serological testing. For 78% (435 of 559 participants), data were available for both time points. At study start, antibodies against SARS-CoV-2 were detected in 1 of 381 children (0.3%) and 1 of 139 CCWs (0.7%), both with previously documented infection. Seroconversion in modules 1 to 3 (continuous testing) was observed in 4 of 165 children (2.4%) and 0 of 51 CCWs. Seroconversion in module 4 (symptomatic testing) occurred in 4 of 173 children (2.3%) and 5 of 58 CCWs (8.6%). All seroconversions were related to recorded SARS-CoV-2 infections detected by the study procedures, by additional testing or contact tracing of study participants (see eAppendix 2 and eFigure 4 in Supplement 2). Antibody testing did not identify additional hidden infections in modules 1 to 3 or module 4.

### Modeling an Optimal Surveillance Protocol for DCCs

**Figure 3.**
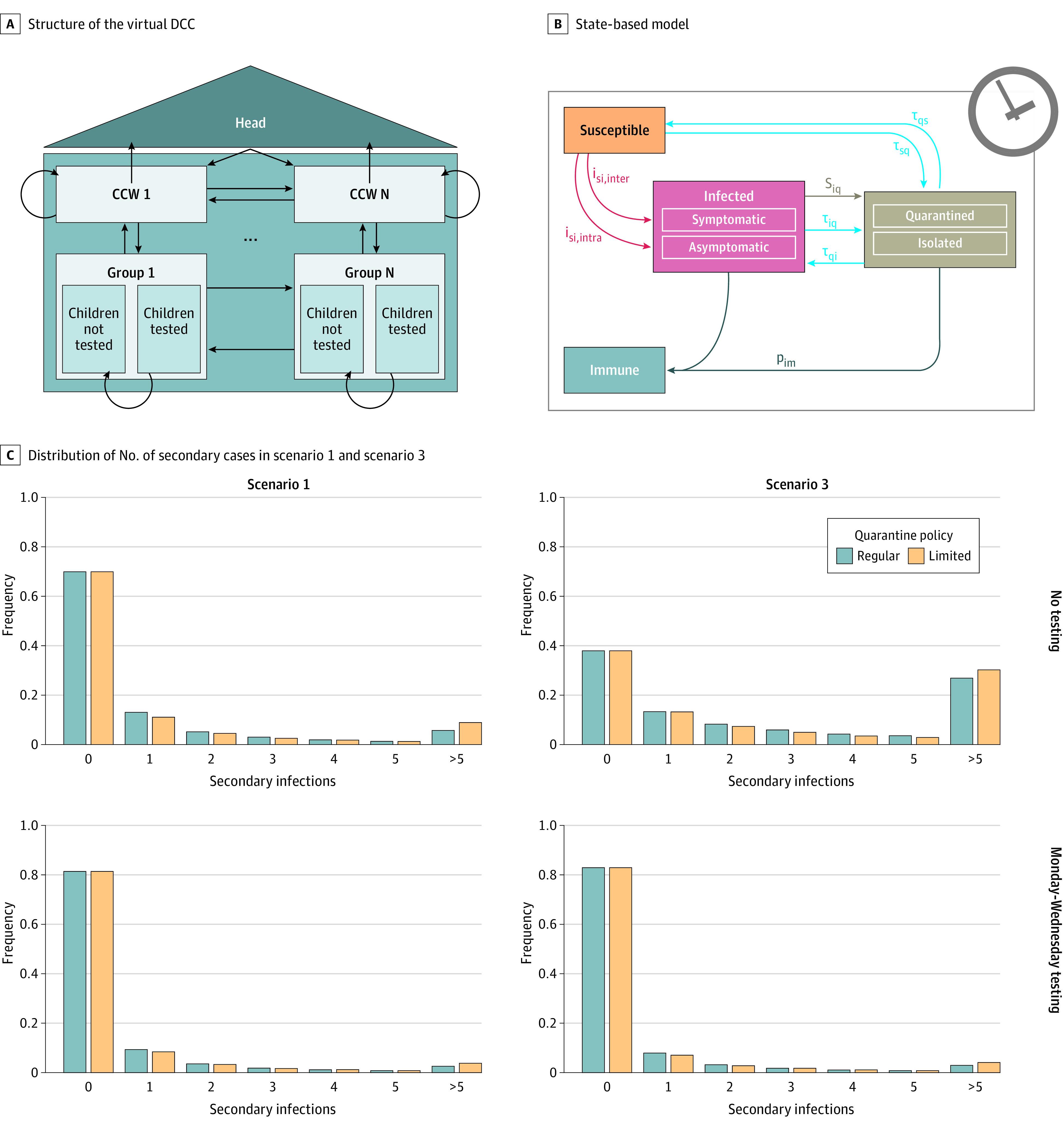
Day Care Center (DCC) Infection Spread Model A, Structure of the virtual DCC. Arrows indicate interactions between different groups. B, State-based model with states depicted in boxes and state transitions as arrows that are defined by their corresponding transition rates. C, Distribution of number of secondary cases after introduction of an index case for each scenario 1 and scenario 3. CCW indicates childcare worker. Transition rates i, p, s, and τ are labeled with subscripts: im, infected-to-immune; inter, intergroup infection; intra, intragroup infection; iq, infected-to-quarantined/isolated; qi, quarantined-to-infected; qs, quarantined-to-susceptible; si, susceptible-to-infected; sq, susceptible-to-quarantine.

Although our study estimates feasibility of SARS-CoV-2 surveillance in DCCs, it does not address efficiency. Thus, we developed an SBM based on parameters identified in our study and literature data (eTable 5, eTable 6, and eTable 7 in Supplement 2) to estimate infection spread in DCCs (Figure 3). This model includes the applied test frequencies and participation rates reached in the feasibility study but also reports expected efficacy in altered settings. We considered 3 different scenarios, with the index patient being an infected child randomly chosen to be symptomatic or asymptomatic and to participate or not in testing (scenario 1), an infected asymptomatic child who does not participate in testing (scenario 2), and an infected CCW who is randomly symptomatic or asymptomatic and participates in testing (scenario 3). For all scenarios, higher participation rates and higher test frequencies reduce the ASI in DCCs (see eFigure 5, eFigure 6, and eFigure 7 in Supplement 2 for scenarios 1-3). In scenario 1, for the test participation of children of 50%, compared with no testing for the regular quarantine policy, Monday testing will reduce ASI by 39.24%, Monday-Wednesday testing will reduce ASI by 50.26%, and Monday-Wednesday-Friday testing will reduce ASI by 55.28%. In scenario 3, for the test participation of children of 50%, compared with no testing for the regular quarantine policy, Monday testing will reduce ASI by 70.11%, Monday-Wednesday testing will reduce ASI by 87.01%, and Monday-Wednesday-Friday testing will reduce the ASI by 94.06%. Furthermore, the ASI is consistently higher and test participation rate has only a minor impact (eFigure 7 in Supplement 2) because CCWs have more contact possibilities and the average viral load peak and the viral aerosol transmission is higher for adults.^22,23^ Efficacy of surveillance testing depends on rapid reporting of results within 1 day. ASI for all relevant scenarios increased with a longer time to result (1.5 days) (eFigure 8, eFigure 9, and eFigure 10 in Supplement 2). ASI also depended on the specific days that are selected for regular testing, with Monday being an optimal testing day. Importantly, for all scenarios, an ASI less than 1 could be realized independently of the quarantine policy with a test frequency of twice per week (including Monday as testing day) and a test participation rate of at least 50% (eFigure 5 in Supplement 2).

## Discussion

To avoid complete DCC closures, alternative approaches that enable safe childcare in a pandemic are urgently required. Combining a real-life feasibility study with mathematical modeling, the results of this nonrandomized controlled trial allow us to define important parameters enabling effective SARS-CoV-2 surveillance in DCCs. Importantly, we can show that long-term surveillance testing is feasible and well accepted by children, parents, and CCWs, with long-term acceptance rates greater than 65% when using noninvasive saliva sampling. Efforts for collecting saliva specimens at home were negligible and conceived as unproblematic by a large majority of parents. Although a symptomatic testing approach identifies more cases compared with continuous testing of asymptomatic individuals, our data suggest that continuous testing of asymptomatic individuals is better suited to detect cases early and avoid transmission within DCCs. Importantly, continuous testing but not on-demand symptomatic testing significantly improved the parents’ sense of security and avoided an increase of anxiety and depression. Despite a regional 7-day incidence of 20 to 152 cases per 100 000 population, the frequency of SARS-CoV-2 infections found in DCCs was very low and comparable to other studies.^18,19,24,25,26,27^ During our study, introduction of SARS-CoV-2 into DCCs occurred mainly via adult index cases. This observation supports early vaccination of CCWs, as has been prioritized in many countries including Germany. Importantly, serological testing before and after surveillance indicated that few or no infections occurred unnoticed.

Although our study clearly shows that surveillance testing is feasible, it is very difficult to evaluate effectiveness thoroughly in a real-life study. Thus, we developed a mathematical model that specifically addresses viral spread in DCCs. Because the number of individuals in DCCs is generally low, and, thus, stochastic variation is high, we used an SBM to account for the stochasticity of the system. SBMs have been used previously to model infections, where low numbers of individuals, high variation, and discrete events need to be considered.^28,29,30,31^ Modeling based on literature data and study data suggests that continuous testing in DCCs under realistic conditions (biweekly testing including Monday as a testing day, with >50% participation rate) can reduce the ASI to less than 1. Importantly, our model estimates that under these conditions, a limited quarantine policy, isolating cases but avoiding quarantine for contact persons, is feasible and does not result in significantly higher numbers of secondary infections. This model is flexible and parameters (eg, those specific for virus variants) can be adapted.

### Limitations

Limitations of our study include a nonrandomized design, a lack of control for saliva sampling at home, and the exclusion of children younger than 2 years in module 3. Furthermore, the feasibility study was performed in only 1 city and was largely conducted before the emergence of highly transmittable SARS-CoV-2 variants.^32^ We cannot exclude clustering of households and DCCs in the different modules; to minimize its effect, only 1 person per household was included in the descriptive and multivariable models.

## Conclusions

In this nonrandomized controlled trial combined with mathematical modeling, we explored avenues for continuous and safe preschool childcare during the SARS-CoV-2 pandemic. Our findings suggest that evidence-based continuous surveillance can help to avoid the negative impact of closure of DCCs.

## Notes

Supplement 1.
Trial Protocol

Supplement 2. **eAppendix 1.** Supplemental Methods **eAppendix 2.** Supplemental Results **eTable 1.** Characteristics and Attitudes of Participating Parents **eTable 2. **Characteristics and Attitudes of Participating CCW **eTable 3. **Factors Associated With and Reasons for Declined Participation in Parents **eTable 4.** Dropout Rates in Modules 1-3 and Unsuccessful Testing in Module 4 **eTable 5.** Number of Samples Tested for SARS-CoV-2 in DCC With Continuous Surveillance (Modules 1-3) **eTable 6.** Model Parameters Used for All Simulations **eTable 7. **Varied Parameters and Processes in the Model **eFigure 1.** Flowcharts of Wue-KiTa-CoV Study **eFigure 2.** General and Specific Measures Implemented Against SARS-CoV-2 **eFigure 3.** Final Assessment of Study Measures by Parents and CCWs **eFigure 4.** Events Associated With Potential Introduction of SARS-CoV-2 Into a DCC **eFigure 5.** Modeling Results Scenario 1 **eFigure 6.** Simulation Results of the Virtual Infection Spread Model for Scenario 2 **eFigure 7.** Modeling Results Scenario 3 **eFigure 8.** Modeling: Effects of Longer Time to Result on Scenario 1 **eFigure 9.** Modeling: Effects of Longer Time to Result on Scenario 2 **eFigure 10.** Modeling: Effects of Longer Time to Result on Scenario 3
eReferences

Supplement 3.
Wü-KiTa-CoV-Study Group Members

Supplement 4.
Data Sharing Statement
