# Supplementary material for: Feasibility of SARS-CoV-2 Surveillance Testing Among Children and Childcare Workers at German Day Care Centers: A Nonrandomized Controlled Trial
Source: JAMA Netw Open. 2022 Jan 4;5(1):e2142057. doi: 10.1001/jamanetworkopen.2021.42057 (PMC8728621; doi:10.1001/jamanetworkopen.2021.42057)
Supplement: Supplement 1. — Trial Protocol [file jamanetwopen-e2142057-s001.pdf]

Wü-KiTa-CoV

InfectControl Würzburg Childcare Study during the COVID-19 pandemic:

Acceptability of various surveillance protocols for the timely detection of SARS-CoV-2 infections and their spread in daycare centers

(Feasibility study)

Study lead

Prof. Dr. med. Oliver Kurzai

Prof. Dr. med. Johannes Liese, MSc

Date of version:

July 24, 2020

Final Version 1.28

The contents of this study protocol are confidential and must not be passed on to third parties.

TABLE OF CONTENTS

PAGE

1 GENERAL INFORMATION 4
1.1 Participating institutions and persons responsible 4
1.2 Signatures 6
1.3 Abbreviations 7
1.4 Synopsis 8
1.5 Flow chart study process recruitment/data collection 11
1.6 Background: COVID-19 and daycare 12
1.7 Research questions and rationale 16
1.8 Risk benefit assessment 18
2 STUDY OBJECTIVES 21
2.1 Primary Hypotheses 21
2.2 Secondary Hypotheses 21
2.3 Study design und time schedule 21
3 STUDY POPULATION 23
3.1 Selection of participating centers 23
3.2 Inclusion criteria for participants in the daycare centers 23
3.3 Exclusion criteria for participants in the daycare centers 23
4 STUDY PROCESS 24
4.1 Recruitment of study centers and participants 24
4.2 Informed Consent Process 24
4.3 Questionnaires, interviews and clinical examinations 25
5 PARTICIPANT SAFETY 31
6 DATA MANAGEMENT 32
6.1 Source data and source material 32
6.2 Study database 33
6.3 Laboratory methods 34
6.4 Sampling 34
7 BIOMETRICAL ASPECTS 36
7.1 Endpoints 36
7.2 Data analysis methods 38
7.3 Populations for analysis 39
7.4 Timing of analysis 39
7.5 Sample size discussion and power analysis 39
8 QUALITY ASSURANCE AND QUALITY CONTROL 42
8.1 Date flow chart / responsibilities 44
9 PUBLICATION OF RESULTS 45
10 INSURANCE COVER FOR PARTICIPANTS 46
11 ETHICAL CONSIDERATIONS 47
11.1 Ethics committee approval 47
11.2 Participant information and informed consent to sample collection 47
11.3 Informed consent to study participation 47

11.4 Data use, storage and disclosure ... 47

12 REFERENCES ... 49

13 APPENDIX ... 51

13.1 Participant information sheets ... 51
13.2 Informed consent forms ... 51
13.3 Data collection instruments ... 51
13.4 Additional information material ... 51

1 GENERAL INFORMATION

1.1 Participating institutions and persons responsible

Study lead:	Prof. Dr. med. Oliver Kurzai<br>Institute of Hygiene and Microbiology, University of Würzburg<br>Josef-Schneider-Str. 2-E1, 97080 Würzburg
	Prof. Dr. med. Johannes Liese, MSc<br>Department of Pediatrics, University Hospital Würzburg<br>Josef-Schneider-Str. 2-D31, 97080 Würzburg
Research scientists:	Prof. Dr. med. Lars Dölken<br>Institute of Virology, University Würzburg<br>Versbacher Str. 7, 97078 Würzburg
	Prof. Dr. med. Ildikó Gágyor<br>Department for General Medicine, University Hospital Würzburg<br>Josef-Schneider-Str. 2-D7, 97080 Würzburg
	Prof. Dr. med. Thomas Keil, MSc<br>Institute for Clinical Epidemiology and Biometry,<br>University of Würzburg, Josef-Schneider-Str. 2-D7, 97080 Würzburg
	Prof. Dr. med. Marcel Romanos<br>Department of Child and Adolescent Psychiatry, Psychosomatics and Psychotherapy, University Hospital Würzburg<br>Margarete-Höppel-Platz 1, 97080 Würzburg
	PD Dr. rer.nat. Andrea Streng<br>Department of Pediatrics, University Hospital Würzburg<br>Josef-Schneider-Str. 2-D31, 97080 Würzburg
	Dr. med. Benedikt Weißbrich<br>Institute of Virology, University Würzburg<br>Versbacher Str. 7, 97078 Würzburg
Advisers:	Dr. Hülya Düber<br>Head of the Office for youth, family and social affairs / Würzburg City Council, Stadt Würzburg<br>Karmelitenstraße 43, 97070 Würzburg
	Monika Kraft<br>Deputy head of the Office for youth, family and social affairs / Würzburg City Council, Stadt Würzburg
	Dr. med. Johann Löw<br>Head of local health authority (“Gesundheitsamt“) Würzburg<br><br>Dr. Barbara Finkenberg<br>Gesundheitsamt Würzburg

Study coordination:	Dr. med. Johannes Forster<br>Institute of Hygiene and Microbiology, University of Würzburg
	PD Dr. rer.nat. Andrea Streng<br>Department of Pediatrics, University Hospital Würzburg
Biometry and data management:	Prof. Dr. med. Peter Heuschmann<br>Viktoria Rücker<br>Institute for Clinical Epidemiology and Biometry,<br>University of Würzburg, Josef-Schneider-Str. 2-D7, 97080 Würzburg

1.2 Signatures

Study lead

____

Place, date

____

Prof. Dr. Oliver Kurzai

Institute of Hygiene and Microbiology, University of Würzburg

____

Place, date

____

Prof. Dr. med. Johannes Liese, MSc

Department of Pediatrics, University Hospital Würzburg

1.3 Abbreviations

COVID-19	Coronavirus disease 2019
ELISA	Enzyme-linked immunosorbent assay
FFP	Filtering face piece
PCR	Polymerase chain reaction
SARS-CoV-2	Severe acute respiratory syndrome coronavirus 2

1.4 Synopsis

Study title	Wü-KiTa-CoV: Würzburg Daycare Study during the COVID-19 pandemic:<br>Acceptability of various surveillance protocols for the timely detection of SARS-CoV-2 infections and their spread in daycare centers
Short title	Würzburg Daycare Study into the acceptability of SARS-CoV-2 surveillance
Study aims	Evaluation of four different SARS-CoV-2 surveillance protocols with regard to their acceptance by the children, parents/guardians and childcare workers.<br>The results of this feasibility study are intended to serve as a basis for planning a subsequent multicentre, cluster randomised study, which will aim to evaluate surveillance protocols with good participant acceptability with respect to their effectiveness for the detection of SARS-CoV-2 infections and their spread in daycare centers.
Design	Feasibility study (open, multicentre longitudinal intervention study)
Population	Children aged 1-8 years attending one of up to 7-11 pre-defined daycare centers (crèches, nursery schools/kindergartens) in Würzburg, their families (only module 4) and childcare workers in these daycare centers
Sample size	Surveillance protocols:<br>Module 1 (intensive monitoring: mid-turbinate swabs twice per week):<br>1 daycare centre, ≥100 children, > 5 childcare workers<br>Module 2 (extensive monitoring: mid-turbinate swab once per week):<br>1 daycare centre, ≥100 children, > 5 childcare workers<br>Module 3 (intensive monitoring: saliva samples twice per week): 1-3 daycare centers, in total ≥175 children aged 2 years and older, > 10 childcare workers<br>Module 4 (symptom-based monitoring of household members of the children and childcare workers): up to 5 daycare centers, in total ≥ 250 children, > 25 childcare workers
Tests	PCR of mid-turbinate swab samples and saliva samples; in module 4 PCR of oropharyngeal swabs<br>Antibody detection (point of care test / ELISA of blood serum)
Endpoints	Primary endpoints for the different surveillance protocols:<br>• [Modules 1-3]: Rate of acceptance of the respective surveillance protocol defined as: proportion of

	children/childcare workers with "successful" participation in sample collection (mid-turbinate swab or saliva sample) among all asymptomatic children and childcare workers in the daycare centre.<br><br>Asymptomatic is in this case defined as: absence of respiratory symptoms that would lead to the child / childcare worker being excluded from attending the daycare centre according to the hygiene measures in place at the time of the scheduled sample collection.<br><br>Parent/child groups and childcare workers who do not consent to participation in the study will be included in the calculations as "non-successful" participants. Each study participant will be classed as "successful" with respect to sample collection if at least 60% of all scheduled samples were collected successfully.<br><br>A surveillance protocol will be classed as "successful" if the rate of acceptance exceeds the following level:<br><br>≥30% (Module 1: intensive monitoring / mid-turbinate swabs)<br><br>≥37.5% (Module 2: extensive monitoring / mid-turbinate swabs)<br><br>≥37.5% (Module 3: intensive monitoring / saliva samples)<br><br>• [Module 4]: Rate of acceptance of the surveillance measure:<br><br>Proportion of cases with successful sample collection (i.e. completion of sample collection and diagnostic analysis – or receipt of test result in the case of external testing – within 72h after first telephone contact with hotline) among all cases of sample collection recommended for symptomatic children, symptomatic childcare workers or symptomatic household members of children/childcare workers<br><br>The surveillance protocol will be classed as "successful" if the rate of acceptance exceeds the following level:<br><br>≥70% (Module 4: symptom-based monitoring)<br><br><u>Secondary endpoints (inter alia):</u><br><br>• Acceptance of mid-turbinate swab sample collection (proportion of successfully completed swabs), acceptance of saliva sampling (proportion of successfully performed sampling), acceptance of blood sampling (proportion of blood samples performed in relation to all scheduled blood samples)<br><br>• Satisfaction and sense of security of families/children (using questionnaires and validated scores)

	• Costs per child and sample collection (based on direct costs).<br><br><u>Secondary exploratory endpoints in the event of SARS-CoV-2 cases being detected</u><br><br>• Proportion of positive PCR tests<br>• Proportion of positive serological tests, correlation between positive PCR and seroconversion between first and second serological test<br>• Number and cost of swabs (throat and nose) / saliva samples per detected case of SARS-CoV-2 infection
Statistical Analyses	The primary endpoints will be estimated as 95% confidence intervals using the Wilson score method. Due to the purely exploratory nature of all analyses and the fact that the four modules will be implemented in four separate daycare centers, the significance level will not be adjusted for multiple testing. The significance level will be set to 5% for all analyses. In a first step, descriptive analyses (frequency (percent), mean (SD) or median (IQR)) will be reported for all endpoints. For comparing the four groups with respect to some of the secondary endpoints, a chi-squared-test, ANOVA or Kruskal Wallis test will be used as appropriate. Subsequently, pairwise comparisons will be carried out using appropriate post-hoc tests. Analysis of the development of the psychosocial factors over time will be stratified by monitoring module and will use tests for repeated measurements such as the McNemar test, Repeated Measurements ANOVA and the Friedman test. In addition, baseline values and the follow-up values after 12 weeks will be compared between modules using appropriate tests such as the chi-squared test and ANOVA or non-parametric tests followed by post-hoc tests. Potential predictors of the rate of acceptance will be analysed (stratified by module) in univariable analyses using the chi-squared test and, if a sufficient sample size is obtained, additionally using multivariable logistic regression. If possible, an ICC value (intraclass correlation) for the daycare centers will be calculated as a basis for sample size estimation for a future cluster randomised study. All analyses will be performed in SAS, R or SPSS.
Time schedule	Data collection period of 12 weeks.<br><br>All modules to begin in parallel, if possible directly after the re-opening of daycare centers after the summer holidays. Estimated duration until the beginning of the Christmas holidays, with a minimum duration of 12 weeks.

1.5 Flow chart study process recruitment/data collection

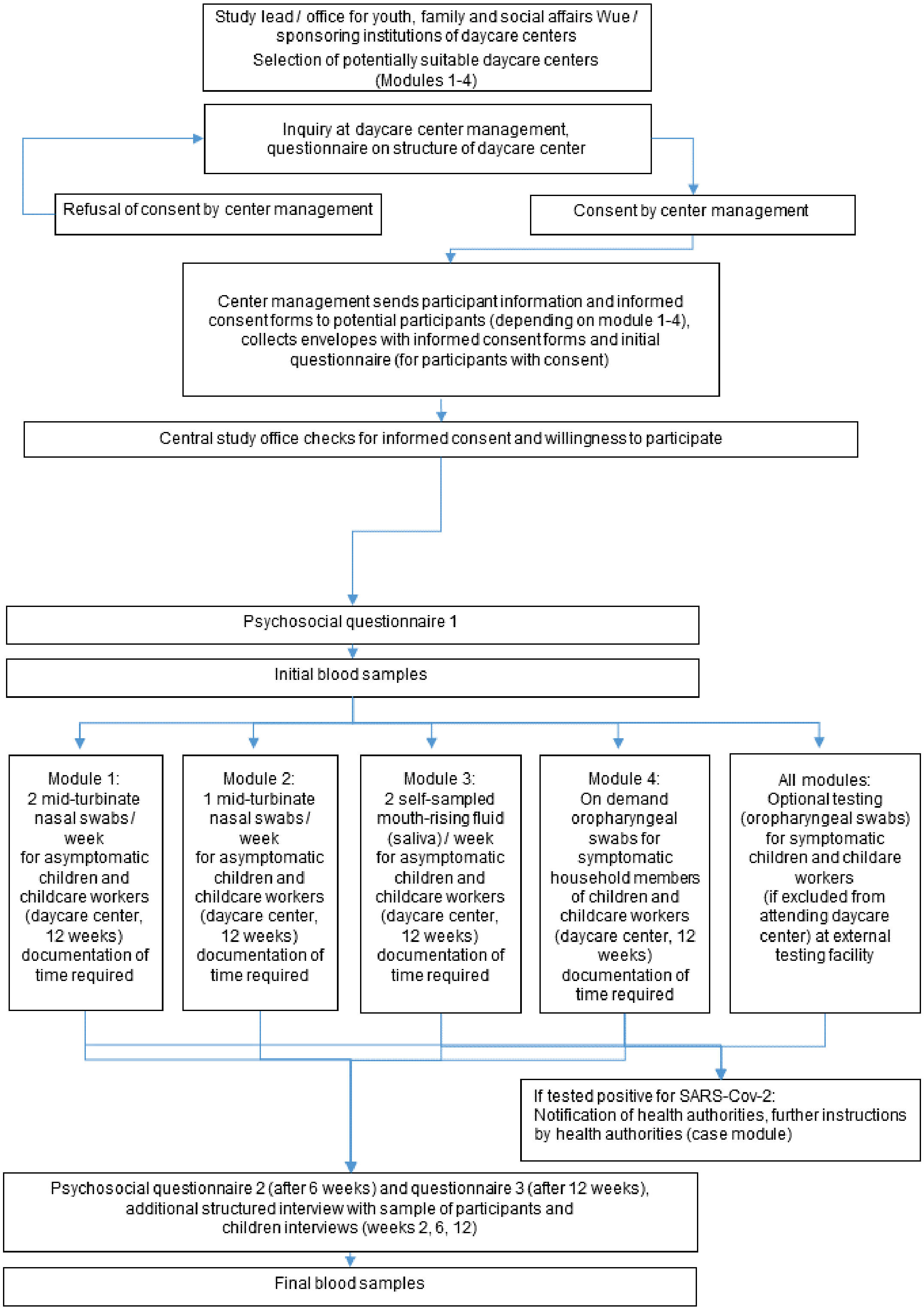

1.6 Background: COVID-19 and daycare

SARS-Cov-2/COVID-19 pandemic

In December 2019 the novel coronavirus SARS-CoV-2 began to spread worldwide. On 11 March 2020 the outbreak was officially declared a pandemic by the WHO. The SARS-CoV-2 virus belongs to the coronavirus family. Human pathogenic coronaviruses usually cause infections of the upper respiratory tract in infected humans. The spectrum of clinical manifestations ranges from normal cold symptoms to severe courses of disease with potential inflammatory-cardiovascular and neurological implications [1].

The disease caused by SARS-CoV-2 is termed COVID-19 („**Co**rona**vi**rus **d**isease 20**19**“). According to the Robert Koch Institute, the course of disease is unspecific, exhibits marked diversity and varies greatly between individuals, from completely asymptomatic forms to cases of severe pneumonia with respiratory failure, multi-organ involvement and death. The cumulated data on cases with information on symptoms recorded in the European Surveillance System TESSy (n = 100,233 from 12 European countries; as of 21 April 2020) shows the most common symptoms reported to be loss or alteration of taste or smell, fever, cough and sore throat [2]. According to the “Epidemiologisches Bulletin“ of the Robert Koch Institute dated 23 April 2020, 8–10 % of cases reported in Germany are hospitalised, with only very rare instances of hospitalisation in children. Overall, children and adolescents develop COVID-19 less frequently than adults and those children and adolescents who do develop COVID-19 usually have predominantly mild, uncomplicated infections of the upper respiratory tract; severe complications are rare [3].

Following the initial spread of the virus in Asia, the WHO European Region became the epicentre of the pandemic from mid March 2020. As of 28 April 2020, the European Region accounted for 63% of worldwide mortality due to the virus [4]. Meanwhile, Russia, Central America, South America and the USA account for the majority of new diagnoses. The epidemiological situation in Africa is largely unclear.

The global outbreak of SARS-CoV-2 has made dramatically clear that new infectious diseases can present considerable and severe challenges at any time even to industrialised countries with an excellent healthcare infrastructure. The fight against the pandemic in Germany has included drastic measures placing considerable restrictions on everyone’s daily lives. Examples of such measures are the cancellation of large events, the focusing of inpatient healthcare on the treatment of COVID-19 patients, worldwide travel warnings issued by the foreign office, far-reaching restrictions on leaving the house and limits on social contacts, the closure of shops and restaurants and the introduction of compulsory face masks in many areas of public life.

Effects of the COVID-19 pandemic on daycare

Among the restrictions with particular impact are the measures concerning daycare. These measures have considerable psychosocial consequences not only for the children, but also their families. Relevant professional associations have pointed out that the closure of daycare centers, playgrounds, schools and sports facilities means that children are deprived of essential prerequisites for their healthy physical, mental and social development [5]. It has been argued that, in the light of the mostly mild course of disease in children and adolescents, the

school closures are not primarily aimed at protecting the children, but at containing the pandemic and thereby protecting the vulnerable older age groups, and point out that the school closures are contrary to children's right to education (UN Convention on the Rights of the Child, Article 28) [6]. In addition, the requirement for parents to fulfil their job responsibilities from home at the same time as caring for their children at home means that some parents will be unable to meet both or even either of these demands sufficiently. The closure of daycare centers and schools is therefore also associated with considerable economic consequences. Furthermore, many of the support and assistance services offered for families (youth welfare offices, non-governmental social services) have become largely unavailable to families due to the physical distancing measures, and outreach work can no longer be guaranteed. Youth welfare offices are therefore not in a position to be able to adequately fulfil their safeguarding responsibilities as advocates of the children and there is an increased risk of cases where the child's welfare is threatened.

Clinical manifestations of SARS-CoV-2 infections in children

An infection with SARS-CoV-2 usually leads to milder clinical symptoms in children than in adults:

A systematic review based on 14 publications from China showed that only 6% of 2,228 children developed a severe course of disease (72% mild, 22% moderate). The publications analysed reported only two fatal cases (one 14-year-old child, one newborn) [7]. Another systematic review based on 45 published case series and case reports concluded that children accounted for 1-5% of the COVID-19 cases diagnosed and that they typically showed a milder course of disease than adults. This publication also reported only individual fatal cases in children [8].

A rare but severe complication of SARS-CoV-2 infections in children which has been reported is the multisystem inflammatory syndrome (Kawasaki-like syndrome) with circulatory dysfunction and macrophage activation syndrome [9,10].

First analyses from Europe (Italy) have also confirmed a generally milder course of SARS-CoV-2 infection in children [11]. In this analysis of 168 laboratory confirmed pediatric cases (median age 2.3 years), of which the majority (67.9%) were hospitalised and a large proportion (19.6%) had comorbidities, only two children required intensive care treatment.

In Germany, 0.1% of all registered COVID-19 cases were aged between 0 and 4 years, 0.23% between 5 and 14 years. The agewise incidence for 0-14 year-olds was reported to lie between 45 and 57 per 100,000 inhabitants, considerably lower than in all other age groups (167-384/100,000 inhabitants) [12]. The Robert Koch Institute concludes that children are less likely to be infected with SARS-CoV-2 than adults according the majority of available studies. According to the Robert Koch Institute, the symptoms of disease in children appear to be often less pronounced than in adults. Asymptomatic infections also seem to be fairly frequent in children (in up to 28% of cases).

The role of children and daycare centers in the spread of SARS-CoV-2

To date, evidence on the role of children in the spread of SARS-CoV-2 remains inconclusive. Publications on infection clusters in France and Australia did not report any cases of the virus having been transmitted to other adults or children by infected children [13,14]. In a large

surveillance study in Iceland, children were more rarely affected than adults both in an at-risk population (6.7% vs. 13.7%) and in a population screening programme (0% vs. 0.8%) [15]. Similar results were obtained in an analysis of the 2,812 inhabitants of the town Vo (Veneto, Italy) during an outbreak: No case was reported for children below the age of 10 years, whereas SARS-CoV-2 was detected in 2.8% of adults [16]. A Japanese study which analysed suspected cases and their contact persons reported the attack rate to be lowest in the age groups 0-10 years and 10-20 years [17]. Reports from Sweden, where no restrictions were placed on daycare services and children are nonetheless underrepresented among the detected cases of infection, might also suggest a lower rate of infection in children. The authors of a systematic review – limited by a poor data basis – arrived at the conclusion that the contribution of school closures to the containment of the pandemic was limited [18].

Other analyses however show that children are in principle as likely as adults to catch the infection [19]. Early on in the course of the pandemic, it was already pointed out that children might in fact be efficient transmitters of SARS-CoV-2 due to a lack of symptoms at the same time as ongoing virus replication. Reports on the efficient spread of SARS-CoV-2 in a school in France were in accordance with this theory [20]. In this school with predominantly older pupils, the attack rate was 40.9% for pupils/teachers and 10.9% for family members. An analysis of virus test results of respiratory material in cases with confirmed SARS-CoV-2 did not give any indication of a relevant reduction in quantitative viral load in the upper respiratory tract of children compared to adults [21]. The authors therefore question the theory of children being less infectious than adults. At the same time, however, they point out that the lower breathing volume and the weaker coughing force of toddlers might lead to children being less infectious than adults with the same levels of viral load.

Overall, the predominant view is that children usually show asymptomatic or mainly mild courses of COVID-19 disease [6]. The question of whether or not they also play a lesser role in the transmission of SARS-CoV-2 remains unresolved. A modelling study on the effectiveness of societal measures aimed at curtailing transmission of SARS-CoV-2 in 41 (mostly European) countries, which was published as a "pre-print" version in June 2020, arrives at the surprising conclusion that – compared to other measures such as curfews and the closure of shops – school closures were on average the most effective measure for reducing transmission rates [22].

Daycare related measures aimed at the containment of the COVID-19 pandemic

From 16 March 2020 on, all schools and daycare centers in Bavaria were closed in an effort to contain the spread of SARS-CoV-2. This measure was based on the assumption that even oligosymptomatic and asymptomatic children are infectious and therefore contribute to the spread of the virus in society if they attend daycare centers. The scientific grounds for this assumption are based almost exclusively on analyses of influenza outbreaks and may possibly not be transferable to the situation of SARS-CoV-2 [6].

After the large-scale closures, only the provision of so-called emergency daycare services, e.g. for the children of parents working in critical jobs, was allowed, and the degree to which these services were available varied greatly. The rulings regulating which parents had the right to make use of the emergency daycare services were adapted several times during the further course of the pandemic. So far, daycare centers in which cases of SARS-CoV-2 were detected have as a rule been closed for a period of at least two weeks by the local health authorities.

Re-opening of daycare services began in May 2020 and continued step by step in June and July 2020. The main reason for the hesitant step by step re-opening is the fact that the proven hygiene measures against SARS-CoV-2 cannot be implemented in daycare centers. In particular, the rules for systematically keeping a distance of 1.5 m to other persons and for wearing compulsory face masks in situations where it is not feasible to keep this distance cannot reasonably be implemented in daycare centers, especially when caring for small children.

Given the fact that the implementation of hygiene measures in daycare centers is barely feasible, effective and reliable surveillance of children attending daycare centers is crucial. Close-knit monitoring programmes that can be carried out easily and on a large scale in adult populations are however not directly transferable to child populations due to a lack of comprehension and cooperation. So far, no programmes for the effective monitoring of children in daycare centers in Germany have been developed. Depending on the prevalence of COVID-19 in the general population, such measures will in the future possibly need to be implemented on a large scale and for extended periods. A critical prerequisite for such monitoring programmes, besides a high level of effectiveness in detecting SARS-CoV-2 cases, is longer-term acceptance of the measures by all those concerned (children, their families, childcare workers, sponsoring institutions). A theoretically highly effective measure for the detection of cases of infection, such as daily swabs of all children in the daycare centre and testing by PCR, will not lead to the desired success if it is not accepted by those concerned. Conversely, a method such as surveillance of the household members rather than the children themselves for respiratory symptoms or the use of (possibly less sensitive) saliva sampling could be expected to achieve high levels of acceptance, but might prove not to be sufficiently effective in terms of preventing the spread of infections in the daycare centre. It is therefore important to evaluate the feasibility and acceptability of potential monitoring measures before evaluating the effectiveness.

Aims of the feasibility study

With the “Wü-KiTa-CoV“ study we plan to test and compare four alternative surveillance programmes for SARS-CoV-2 infections and their spread among children and childcare workers in daycare centers with regard to participants’ acceptance and aspects of practical feasibility. In addition to the acceptability and the technical and logistical feasibility of the measures, their psychosocial effects on children, families and childcare workers will be explored using questionnaires and interviews, and the costs incurred per child will be calculated based on the direct costs (material, personnel, testing).

The overarching aim of this feasibility study is to identify surveillance programmes with good acceptability, which can then be examined with regard to effectiveness in a subsequent study.

Preconditions for planning the subsequent study

Planning of the subsequent study will be subject to the precondition that at least one of the surveillance programmes examined in the feasibility study achieves an acceptance rate exceeding the pre-defined success criteria over the 12-week observation period. Under this precondition it can reasonably be expected that the unmodified continuation of the surveillance

programme in the subsequent study will not result in higher drop out rates and that use of an optimised version of the surveillance programme may even achieve lower drop out rates.

Aims of the intended subsequent study

Based on the results of the feasibility study with respect to acceptability and feasibility, up to two optimised surveillance programmes will be selected, and the effectiveness of these surveillance programmes in terms of timely detection of SARS-CoV-2 infections in children attending daycare centers will be analysed in a subsequent multicentre cluster-randomised study. The ultimate aim is to achieve early interruption of virus transmission in the daycare centre (and, beyond that, in the family) and therefore enable continuity in the provision of daycare services at the centre as far as possible. Daycare centers without any surveillance programmes implemented will form the control group in the subsequent study.

1.7 Research questions and rationale

The Wü-KiTa-CoV study is a feasibility study into the acceptability and practical feasibility of alternative surveillance measures (modules 1-4) in daycare centers.

In modules 1-3, asymptomatic children (and childcare workers) will be tested for SARS-CoV-2 infection on a regular basis using different methods. Asymptomatic is in this case defined as: absence of respiratory symptoms that would lead to the child / childcare worker being excluded from attending the daycare centre according to the hygiene measures in place at the time of the scheduled sample collection.

In module 4 it is not asymptomatic children attending the daycare centre (and childcare workers) who will be monitored; instead, any members of the same households as the children and childcare workers will be tested in the event of symptoms compatible with COVID-19 (“symptom-based monitoring”), i.e. symptomatic children, symptomatic childcare workers and any symptomatic household members.

The results of this study shall serve as the basis for the conceptualisation and planning of a subsequent multicentre, cluster randomised intervention study into the effectiveness of surveillance programmes with good acceptability.

The feasibility study detailed in this protocol primarily aims to answer the following research question:

I. Are surveillance programmes accepted by the children, their families and the childcare workers (*acceptance*)?

In addition, the following research questions will be addressed:

II. To what extent are surveillance programmes in daycare centers feasible with respect to staff requirements, logistical aspects and organisational aspects in the centers (*feasibility*)?

III. Which factors are potential *predictors of acceptance* of surveillance programmes in daycare centers?

IV. What are the *psychosocial effects* of an infection monitoring programme in a daycare setting on children, their families and childcare workers?

V. What is the *SARS-CoV-2 seroprevalence* among children and childcare workers in Würzburg and what changes in seroprevalence occur during the study period?

Further research questions:

- What is the proportion of other respiratory viruses in children attending daycare centers and developing respiratory symptoms during the SARS-CoV-2 pandemic?

- The study is furthermore intended to provide initial data on the frequency of SARS-CoV-2 infections in daycare centers (*effectiveness*) and initial data on the *costs* associated with the different surveillance protocols.

1.8 Risk benefit assessment

The overall risks associated with the study can be classified as low. The sampling procedures are standard examination procedures and only slight side effects are usually to be expected, e.g. a feeling of discomfort while having a mid-turbinate swab taken or the development of a small bruise (haematoma) around the prick region during finger-prick testing.

Risks when taking mid-turbinate swabs:

The occurrence of relevant complications when taking mid-turbinate swabs is very rare. In some cases, a feeling of discomfort can be experienced while the mid-turbinate swab is being taken. The subjective perception of a mid-turbinate swab as an unpleasant experience varies greatly between individuals. One of the aims of this study is to determine to what extent regular mid-turbinate swabs are tolerated by the children / childcare workers (cf. study aims).

Risks when taking oropharyngeal swabs (concerns only module 4 und symptomatic persons in modules 1-3):

Having a oropharyngeal swab taken may cause some temporary discomfort and trigger the gag reflex.

Risks when taking saliva samples:

Taking saliva samples involves no relevant risks as this method of sampling at most requires rinsing the mouth with drinking water.

Risks of finger-prick testing:

In rare instances, finger-prick testing can result in a small bruise (haematoma) around the prick region. In very rare cases, finger-prick testing can lead to a local infection of the prick region.

Risks when taking venous blood samples (concerns only children/childcare workers with positive or borderline results in the point of care diagnostics of the finger-prick test):

- **Bruising (haematoma)** around the puncture site due to injury of the vein or surrounding blood vessels during puncturing
- In rare cases **injuries to arteries** and subsequent bleeding
- In very rare cases **infection** of the puncture site
- In very rare cases **nerve injury** with loss or alteration of motor and/or sensory functions, and a very low risk of lasting damage

All blood sampling will be carried out by physicians under the supervision of a pediatrician.

Risk of infection with SARS-CoV-2 for study population

The initial risk of infection with SARS-CoV-2 for the study population is identical to that of all children attending daycare centers in Würzburg. As the implementation of the surveillance programme is expected to lead to an earlier and more efficient detection of new cases, the secondary risk of infection (infection due to transmission of SARS-CoV-2 in the daycare centre by an infected person not yet diagnosed) will possibly be lower for the study population than in the daycare centers not participating in the study.

The epidemiological situation with respect to the spread of SARS-CoV-2 will be monitored throughout the study period. In every instance of a sample taken in the study being tested positive for SARS-CoV-2, the local health authorities will be informed in accordance the legal provisions. The local health authorities will in all cases be responsible for deciding to what extent quarantine measures are necessary and whether the closure of the entire daycare centre or individual groups within the centre is necessary. In the event of a positive SARS-CoV-2 test result in one of the participating daycare centers, the parents/guardians and childcare workers will immediately be informed once more about the necessity for intensive self-monitoring and immediate testing in the case of symptoms. A telephone hotline service will be available to provide advice to parents/guardians and childcare workers.

Within the study programme, all symptomatic children and childcare workers (who are currently excluded from attending the daycare centre and therefore not included in the surveillance of asymptomatic children and childcare workers in modules 1-3) and their household members will also be offered the option of having a oropharyngeal swab for SARS-CoV-2 at the University Hospital Würzburg.

Benefit:

As yet, it is not foreseeable when the SARS-CoV-2 pandemic will end. There is therefore a need for the development of projectable, reliable and robust measures to enable the continuation of daycare services during the pandemic. Such plans will have the potential to support the decision-making process regarding the installation of measures in the event of new local or national outbreaks of infection.

In this feasibility study, various alternative surveillance programmes for asymptomatic children/childcare workers will be compared with a symptom-based surveillance programme (testing of symptomatic household members of children and childcare workers in module 4). In addition, oropharyngeal swabs for symptomatic children and childcare workers with pre-defined symptoms will also be offered in modules 1-3. Using this study design, it will be possible to compare the alternative surveillance approaches in terms of feasibility and acceptability in order to, in the medium and long term, develop optimal protocols for a continuation of daycare services during the SARS-CoV-2 pandemic and evaluate the effectiveness of these protocols. The results can furthermore be used as a basis for the definition of realistic surveillance protocols for future pandemic respiratory viruses.

The systematic testing implemented within the study will increase safety for the children at the daycare centers and the childcare workers. The availability of medical points of contact via the study hotline will guarantee a maximum degree of safety for the parents/guardians and childcare workers. An aspect of particular importance is the fact that the study design will, by

enabling the timely detection of infections, potentially make it possible to continue providing daycare services even in the event of a case of SARS-CoV-2. The decision on the need for closure of the daycare centre will lie entirely in the responsibility of the local health authorities and will be independent of the research study. The implementation of the study's monitoring programme will, however, have the potential to reduce the probability of a closure at short notice and thereby help to avoid the resulting insecurities and the need for parents to care for their children at home.

A further potential benefit of the feasibility study from an infectious epidemiology point of view is the timely identification of potentially infectious children / childcare workers / household members. This is a prerequisite for efficiently breaking chains of infection and preventing further cases of disease. The results of the study will furthermore include recommendations as to which surveillance programmes for daycare centers should be evaluated specifically with respect to their effectiveness in the prevention of infection transmission.

2 STUDY OBJECTIVES

The primary objective of the Wü-KiTa-CoV study is to evaluate various alternative surveillance programmes for the detection of SARS-CoV-2 cases in daycare centers with respect to their acceptability and practical feasibility. In addition, the study aims to analyse potential predictors of acceptability and the psychosocial effects of the permanent monitoring on children, families and childcare workers. The study is furthermore intended to provide initial data on relevant cost factors associated with the different surveillance protocols.

Depending on data availability, the study will, in addition, provide initial data on the detection of SARS-COV-2 cases and seroprevalence data in children attending daycare centers in the Würzburg area.

2.1 Primary Hypotheses

[1] Screening of asymptomatic children and childcare workers in daycare centers for SARS-CoV-2 using mid-turbinate swabs twice or once a week will be accepted with dropout rates remaining below a level of relevance for the respective testing frequency.

[2] Screening of asymptomatic children and childcare workers in daycare centers for SARS-CoV-2 by saliva samples to be handed in twice a week will be accepted without resulting in a relevant dropout rate.

[3] Surveillance by symptom-based testing for SARS-CoV-2 of household members of children or childcare workers in daycare centers using oropharyngeal swabs will be accepted without resulting in a relevant dropout rate.

(For primary endpoints and definitions cf. Chapter 7.1)

2.2 Secondary Hypotheses

[1] Monitoring for SARS-Cov-2 carried out in daycare centers twice [once] a week using mid-turbinate swabs/saliva samples leads to an increased sense of security and satisfaction amongst the children, their families and the childcare workers.

[2] Surveillance by symptom-based testing for SARS-CoV-2 of household members of children or childcare workers in daycare centers using oropharyngeal swabs leads to an increased sense of security and satisfaction amongst the children, their families and the childcare workers.

2.3 Study design und time schedule

2.3.1 Design

Feasibility study (open, multicentre longitudinal intervention study)

2.3.2 Time schedule

After the end of the summer holidays 2020, childcare workers and parents in the selected daycare centers will be provided with information material and their informed consent will be sought. Implementation of the surveillance modules will begin as soon as possible after the end of the summer holidays, with the requirement of a simultaneous start in all daycare centers and modules. The implementation of the modules in the daycare centers will last at least 12 weeks and is expected to end with the start of the Christmas holidays.

**Year**	**2020**																										**2021**												
**Month**	J	J	J	J	A	A	A	A	S	S	S	S	S/O	O	O	O	O	N	N	N	N	D	D	D	D	D	J	J	J	J	F	F	F	F	M	M	M	M	M
**Calendar week**	28	29	30	31	32	33	34	35	36	37	38	39	40	41	42	43	44	45	46	47	48	49	50	51	52	53	1	2	3	4	5	6	7	8	9	10	11	12	13
**Preparation**																																							
Planning meeting with local health authority, childcare centres' sponsoring institutions, Würzburg City Council	X																																						
Finalisation of study protocol, participant information / informed consent forms	X	X	X																																				
Ethics committee approval (preparation of documents, application process)			X	X	X	X																																	
Recruitment of study personnel (physician, study nurse, medical students)		X	X	X	X	X																																	
Specification of standard operating procedures, training for study team					X	X																																	
Information session/material for childcare centres' sponsoring institutions			X	X																																			
Setting up of central study office, administrative database, organisation of laboratory services			X	X	X																																		
Recruitment of study centres							X	X	X	X																													
**Data collection questionnaires / interviews**																																							
Lists of costs, drop out lists										X	X						X	X						X	X														
Initial questionnaire and psychosocial questionnaire										X	X						X	X						X															
Interviews												X	X	X	X	X								X	X														
**Data collection laboratory data**																																							
Seroprevalence testing										X	X													X	X														
Throat swabs modules 1-4												X	X	X	X	X	X	X	X	X	X	X	X																
**Data management**																																							
Entry of data paper based / electronic questionnaires / lists										X	X	X															X	X	X										
Entry of interview data																											X	X	X										
Entry of laboratory data microbiology and virology												X	X	X	X	X	X	X	X	X	X	X	X	X	X	X	X												
Data linkage biometry, data checks																														X									
Finalisation of database																														X									
**Statistical analyses and report**																														X	X	X	X	X					
**Study report**																														X	X	X	X	X					
**Publication**																																		X	X	X	X	X	
**Documentation of records, deregistration of study at ethics committee**																																							X

3 STUDY POPULATION

3.1 Selection of participating centers

The study aims to include between 7 and 11 of the approximately 80 daycare centers located in the town of Würzburg for a study period of 12 weeks. For logistical reasons, larger daycare centers (>= 50 children) will be included preferentially. The selection of daycare centers will be carried out in cooperation with the office for youth, family and social affairs at Würzburg City Council using the following selection criteria:

…for the daycare centers in which modules 1 and 2 will be implemented:

- one daycare centre each, with a minimum of 100 children cared for at the centre and a minimum of 5 childcare workers
- consent of daycare centre's management to implementation of the study

…for the 1-3 daycare centers in which module 3 will be implemented:

- daycare centers with a minimum of 50 children of age ≥ 2 years cared for per centre (at least 175 children across all centers) and a minimum of 5 childcare workers per centre (at least 10 across all centers)
- consent of daycare centre's management to implementation of the study

… for the daycare centers in which module 4 will be implemented:

- between 4 and 6 daycare centers with a minimum of 250 children cared for and a minimum of 25 childcare workers across all centers
- consent of daycare centre's management to implementation of the study

3.2 Inclusion criteria for participants in the daycare centers

- Children aged 1-8 years attending one of the selected daycare centers in Würzburg; childcare workers at the daycare centers; in module 4: in addition, household members of the children and childcare workers
- Written declarations of consent by both parents/guardians to sample collection, written declaration of consent by at least one parent/guardian to questionnaires/interviews, written declaration of consent by adult participants capable of giving consent (childcare workers, household members where relevant in module 4)

3.3 Exclusion criteria for participants in the daycare centers

- Missing written consent
- Module 3: Children aged <2 years at the time of the start of the study

4 STUDY PROCESS

4.1 Recruitment of study centers and participants

The study will be carried out in selected daycare centers in Würzburg. The selection of daycare centers will be carried out in close cooperation with the office for youth, family and social affairs at Würzburg City Council after prior consultation with the centre's management and, where relevant, the centre's sponsoring institution. The initial selection of daycare centers will be independent of the sponsoring institutions in charge. In preparation for the study, potentially suitable daycare centers were selected beforehand and introductory information on the intended study was provided to the centers' managers. The selection criteria for daycare centers are stated in Chapter 3.1. Following consent by the centre's management, all selected daycare centers will be asked to fill in a short questionnaire regarding the structure and organisation of the daycare centre (number of children cared for, number of childcare workers, number of groups, group sizes, childcare workers per group, number of rooms, overall floor area of daycare centre, hygiene checklists specific to SARS-CoV-2).

The overall aim is to achieve participation of all children and childcare workers at each participating daycare centre as far as possible. Recruitment of only a subgroup of children or groups would be problematic due to interactions between children and groups, the shared use of communal spaces and the potential effect of individual participants being stigmatised.

The aim is to implement the study at 7-11 daycare centers in the town of Würzburg for the full duration of 12 weeks. Respiratory sample collection will not begin until the intended number of study centers have been recruited. This serves to ensure that respiratory sample collection can take place within the same 12-week period at all participating daycare centers, as warranted by the highly dynamic pattern of SARS-CoV-2 infections.

4.2 Informed Consent Process

After the consent of the daycare centre's management and sponsoring institution to implementation of the study has been obtained, all children's families and all childcare workers will receive information material and informed consent forms pertaining to the intended study and the specific module to be carried out in their daycare centre. The documents will be distributed by e-mail by the daycare centre's management. Parents/guardians and childcare workers will be asked for their consent to study participation separately for the following parts of the study: (i) initial questionnaire (paper questionnaire) (ii) further questionnaires (including pseudonymised electronic questionnaires, children interviews), (iii) respiratory sample collection, (iv) blood sampling, (v) guided interviews in a sample of participants. The number of parents/guardians and childcare workers declining participation in any part of the study from the outset will be recorded; if possible, they will be asked to state the reasons for their complete denial of consent. For respiratory sample collection and blood sampling, the consent of both parents/guardians will be required, for the other study parts (questionnaires and interviews) the consent of one parent/guardian will be sufficient. Parents/guardians will be asked to print the informed consent forms and hand in the completed forms in a sealed envelope at the daycare centre. If required, printed versions of the informed consent forms can be provided at the daycare centre. Sealable envelopes pre-addressed to the central study office will also be deposited at the daycare centre for the parents/guardians.

Together with the informed consent forms, an initial basic questionnaire will be sent out. The informed consent forms will include the option to give consent separately for this part of the study, and the aim is that as many parents/guardians as possible will participate in this initial questionnaire. If consent to other study parts is denied, the initial questionnaire aims to establish the reasons. In addition, the initial questionnaire will include questions on basic sociodemographic data (e.g. age and sex of the index child, number of family/household members, parents' employment status and educational level). For non-participants (denial of consent to participation in central surveillance measures) who nonetheless have provided written consent to participation in this initial basic questionnaire, the data obtained in the initial questionnaire will be completely anonymised. The initial questionnaire will also include questions on participants' opinions regarding the pandemic (personal assessment of the dangers of SARS-CoV-2 and of the sociopolitical response to the pandemic) and their opinions on vaccinations. Persons consenting to participation in this part of the study will be asked to enclose the initial questionnaire in the envelope with the informed consent forms to be handed in at the daycare centre.

Members of the study team will transport the sealed envelopes with the consent forms and, where applicable, the initial basic questionnaires, to the central study office, where the documents will be recorded and archived. For each daycare centre, participant lists for the individual study parts will be created, and each participant will be assigned an ID number (pseudonymisation).

For these daycare centers participating in the main parts of the study, the proportion of potential participants (children, childcare workers) not participating due to missing informed consent will be recorded.

For all participants, written consent to participation in further questionnaires (including children interviews), respiratory sample collection, serological testing (blood sampling at the beginning and end of the study (cf. 6.4.3)) and in guided interviews (in a sample of participants) will be recorded.

Once a daycare centre's participation in the central monitoring measures of the study has been established, the objectives and contents of the study will be explained in child-friendly language to all children of suitable age groups participating in the respective surveillance module.

4.3 Questionnaires, interviews and clinical examinations

Within this study, four alternative monitoring programmes (4.3.1) for surveillance purposes will be compared. The monitoring measures will be carried out within the same 12-week period at all participating daycare centers. Irrespective of the type of monitoring implemented in the individual daycare centers, the participating children, families and childcare workers will be asked to answer questions regarding the psychosocial implications of the respective monitoring strategy (4.3.1). The SARS-CoV-2 immune status of children and childcare workers for whom informed consent to blood sampling was obtained, in module 4 also parents/guardians and other household members, will be determined using serological testing.

4.3.1 Monitoring programmes (SARS-COV-2)

4.3.1.1 Module 1: Intensive Monitoring – mid-turbinate swabs (1 centre, at least 100 children,

<u>2 mid-turbinate swabs per week)</u>

All participating children and childcare workers in the selected daycare centre will be tested for SARS-CoV-2 with a mid-turbinate swab (cf. 6.4.1) twice a week (on Mondays and Thursdays). Test results will be available 24 hours after taking the swab. If a person is tested positive for SARS-CoV-2, the case module (4.3.1.8) will come into effect.

The monitoring programme will be continued for a period of 12 weeks.

4.3.1.2 Module 2: Extensive Monitoring – mid-turbinate swabs (1 centre, at least 100 children, 1 mid-turbinate swab per week)

All participating children and childcare workers in the selected daycare centre will be tested for SARS-CoV-2 with a mid-turbinate swab (cf. 6.4.1) once a week (on Wednesdays). Test results will be available 24 hours after taking the swab. If a person is tested positive for SARS-CoV-2, the case module (4.3.1.8) will come into effect.

The monitoring programme will be continued for a period of 12 weeks.

4.3.1.3 Module 3: Intensive Monitoring – saliva samples (1-3 centers, at least 175 children overall, 2 saliva samples per week)

All participating children and childcare workers in the selected daycare centers will be tested for SARS-CoV-2 using a saliva sample (cf. 6.4.2.) twice a week (on Tuesdays and Fridays). Test results will be available 24 hours after sampling. If a person is tested positive for SARS-CoV-2, the case module (4.3.1.8) will come into effect.

Children below the age of 2 years will not be included in the monitoring programme of module 3.

4.3.1.4 Module 4: Symptom-based Monitoring (4-6 centers)

The symptom-based monitoring programme is based on a strategy of testing symptomatic children and childcare workers at the daycare centers and also *symptomatic household members* of children and childcare workers in the event of *potential COVID-19 symptoms*. All household members will be informed about potential symptoms of COVID-19 using suitable information material and announcements on notice boards in the daycare centre. They will be asked to contact the central study office directly in the event of symptoms.

Symptomatic participants of module 4 will be offered the option of having a oropharyngeal swab for SARS-CoV-2 in the COVID-19 testing centre at the University Hospital Würzburg, building D20. Symptomatic participants who do not take up this offer will be asked to notify the central study office of the result of the SARS-CoV-2 test performed by third parties (e.g. general practitioner). All symptomatic persons (in the case of symptomatic children the parents/guardians) will be asked to provide information on the time of disease onset and the observed symptoms in a standardised questionnaire.

If a symptomatic child or childcare worker or a symptomatic household member of a child or childcare worker in module 4 is tested positive for SARS-CoV-2, the case module (4.3.1.8) will come into effect.

The symptom-based monitoring programme (module 4) will also include serological tests (4.3.3) and psychosocial questionnaires (4.3.2).

The monitoring programme will be continued for a period of 12 weeks.

4.3.1.5 Additional offers for symptom-based testing of symptomatic children and childcare workers in modules 1-3

According to the regulations in force at present, children with infection symptoms are not allowed to attend daycare centers and symptomatic childcare workers are obliged to stay off work. This restriction will concern all study participants, irrespective of which study module the children and childcare workers are enrolled in.

According to the study protocol for module 4, symptomatic children, symptomatic childcare workers and symptomatic household members of participating children and childcare workers will be tested for SARS-CoV-2.

In modules 1-3, symptomatic children and/or childcare workers and their symptomatic household members will be offered the option to arrange an appointment for a oropharyngeal swab for SARS-CoV-2 in the COVID-19 testing centre at the University Hospital Würzburg, building D20.

Parents/guardians of children participating in modules 1-3 will be given the option to perform sample collection (mid-turbinate swabs, saliva samples) of their symptomatic children themselves.

If they do not take up either of these offers, they will be asked to notify the central study office of the result of the SARS-CoV-2 test performed by third parties (e.g. general practitioner).

All symptomatic persons (in the case of symptomatic children the parents/guardians) will be asked to provide information on the time of disease onset und the observed symptoms in a standardised questionnaire.

Medical care for symptomatic persons will not be provided within this study, but will lie in the responsibility of the participants‘ primary care physicians/pediatricians.

4.3.1.6 Sample collection and sample processing

All staff taking swabs will be protected by an unvalved FFP2 mask or by a face shield.

The mid-turbinate swabs will be taken within the premises of the daycare centre by medically trained staff (medical students working as research assistants, practice nurses, medical technical assistants) in the presence of a pediatrician. If feasible, parents can, at a later stage of the study and after receiving practical instructions from the study team, be given the option to take the swabs themselves at home on the scheduled days and bring the samples to the daycare centre.

Only the initial saliva samples will be taken by medically trained staff within the premises of the daycare centre. During this initial sampling, parents will be instructed how to take the samples themselves. All further saliva samples will be taken by the parents at home in the morning and handed in at the daycare centre. This procedure prevents any potential spread of droplets and aerosols during sampling in the daycare centre. Only children at least 2 years of age and therefore able to give saliva samples will be included in the intensive monitoring programme based on saliva sampling.

Oropharyngeal swabs will be taken in the COVID-19 testing centre at the University Hospital Würzburg, building D20.

Processing, analysis and diagnostic interpretation of swabs/saliva samples will be carried out at the Institute of Hygiene and Microbiology and/or the Institute of Virology at the University of Würzburg. This ensures that the SARS-CoV-2 test result and diagnosis will be available and passed on to the participant within 24 hours of sample collection. In the event of a positive test result, the participant will be notified directly by telephone and the local health authorities will be informed in accordance with §7 Infection Protection Act (“Infektionsschutzgesetz“) (4.3.1.8).

Remaining respiratory sample material will be stored for additional analyses which shall be carried out at a later date for logistical reasons. In these additional analyses, expected to be performed in summer 2021, multiplex PCR will be used to test for other common respiratory viruses (e.g. influenza virus, RSV, rhinovirus, other coronaviruses, adenovirus). As these additional analyses will be carried out some months after completion of the monitoring programme in the daycare centers, participants (parents/guardians) will not receive additional notification of the results of these tests.

4.3.1.7 Notification of positive SARS-CoV-2 results

If symptomatic or asymptomatic children, household members or childcare workers are tested positive for SARS-CoV-2 in the diagnostic laboratory (Institute of Virology / Institute of Hygiene and Microbiology), the local health authorities will be notified in accordance with §7 Infection Protection Act (“Infektionsschutzgesetz“).

In addition, in the event of a positive SARS-CoV-2 test result the participants directly concerned will be informed by one of the physicians at the central study office and will receive standardised written information.

4.3.1.8 Case Module

In the event that a child, household member or childcare worker is tested positive for SARS-CoV-2, the diagnostic laboratory will notify the local health authorities in accordance with §7 Infection Protection Act (“Infektionsschutzgesetz“). Persons tested positive for SARS-CoV-2 will be obliged to follow the instructions of the local health authorities.

The local health authorities will be responsible for deciding whether or not the daycare centre attended by the SARS-CoV-2 positive child / where the SARS-CoV-2 positive childcare worker works can remain open.

Re-admittance to the daycare centre will be subject to the instructions issued by the local health authorities.

Household members of participants tested positive for SARS-CoV-2 will be tested according to the instructions of the local health authorities. Serology for SARS-Cov-2 antibodies will be carried out 2 weeks after the end of the quarantine period imposed by the local health authorities if written informed consent was obtained for venous blood sampling within the study.

Unless the local health authorities issue instructions to the contrary, the daycare unit (the entire daycare centre or the relevant group) of the SARS-CoV-2 positive child/childcare worker will be tested for SARS-CoV-2 twice a week for a duration of 2 weeks after the date of diagnosis.

4.3.2 Psychosocial Dimension (questionnaire)

Successful implementation of the intended monitoring measures is highly dependent upon the acceptance by parents, children and daycare centers. As yet, no systematic data is available on the extent of willingness to participate in such preventive measures in the SARS-CoV-2 pandemic or on the acceptability of such measures. Analysis of the feasibility of the four monitoring protocols must therefore be accompanied by both quantitative and qualitative research elements involving all persons concerned. The feasibility study will furthermore offer the opportunity to gather data on factors potentially affecting acceptance of the monitoring measures in order to identify potential targets for accompanying interventions aimed at increasing the level of acceptance.

The Wü-KiTa-CoV study will gather questionnaire-based data on the acceptance of the respective monitoring protocol by parents/guardians, children and childcare workers three times during the study period (at the beginning of the study period and during and after the implementation of the measures). Families with more than one participating child will be asked to fill in separate questionnaires per child. The following potential predictors of acceptance will be examined:

a) sociodemographic factors (e.g. educational level, place of residence, number and ages of children)

b) Expectations and opinions of the parent/guardian regarding the measures to be introduced

c) Existence and severity of mental health symptoms (anxiety and depression in particular). The assessment of mental health symptoms will be based on questionnaires using validated psychometric instruments/scores (e.g. PHQ4, EQ-5D and a short form of CBCL [23-25]) and a sociodemographic question battery which is also used within the CORONA HEALTH APP Study in cooperation with the Robert-Koch Institute (PI: Rüdiger Pryss, Co-PI: M Romanos).

Qualitative interviews will be conducted in a sample of parents/guardians and with childcare workers from all daycare centers in order to gain further insight into the study participants' expectations, wishes and reservations concerning the measures to be introduced. Follow-up interviews at the end of the intervention programme will seek to establish reasons for acceptance or lack of acceptance in order to identify potential barriers and obstacles to practical implementation and develop ways of minimising these. The same interview partners as in the initial interviews will be invited to these follow-up interviews. Only in the event of their non-participation will new interview partners be recruited. The selection process for inviting participants to qualitative interviews will take sociodemographic characteristics into account as far as possible in order to maximise variation of characteristics with respect to age, sex, educational level, family status and place of residence (purposive sampling). The reasoning for the use of this sampling method is the assumption that the aforementioned sociodemographic characteristics will influence the aspects / topics identified in the interviews.

The interviews will use semi-structured interview guidelines and will be carried out by telephone. Duration of the interviews is estimated at 30 minutes. The interviews will be recorded on tape and subsequently be pseudonymised and transcribed. Coding and analysis of the qualitative interview data will follow a content analysis approach with a mixture of deductive and inductive methods according to Kuckartz [26] using the MAXQDA software. The data obtained in the interviews will be analysed in parallel with the process of conducting interviews, and recruitment of further participants will end once no additional aspects/topics are to be expected (data saturation) [27,28].

Children aged three years and above will be interviewed within the daycare centre three times during the study period by members of the study team who will ask the children one question each regarding their experience of the swab sampling, their motivation to have further swabs taken and their perception of their own cooperation.

4.3.3 Serology

In order to carry out serological testing for SARS-CoV-2 antibodies, finger-prick testing to obtain a few drops of blood from all participants (children, childcare workers) will be used once at the beginning of the study programme and once more at the end of the study. The finger-prick tests will be carried out by trained staff supervised by pediatricians.

Venous blood samples will be taken only in the event of a positive or borderline result in the finger-prick test and only if explicit informed consent for venous blood sampling was obtained (cf. 4.2).

The serological tests will be carried out on site using a point-of-care test and at the Institute of Hygiene and Microbiology and/or the Institute of Virology at the University of Würzburg.

Any sample material remaining after carrying out the tests will be stored for a period of 10 years. Antibody tests for the detection of SARS-CoV-2 antibodies are at present being refined at a high rate of innovation. Storing any remaining sample material will enable renewed testing using more refined techniques in the future.

All study participants (parents/guardians in the case of children) will be notified of the serological test results. No respiratory sample collection and no further blood sampling at the end of the study will be performed in participating children and childcare workers tested positive in the initial serological test.

5 PARTICIPANT SAFETY

All oropharyngeal swabs and blood tests will be taken by medically trained staff who would be able to initiate emergency treatment in the extremely rare event of complications.

The saliva samples and, where applicable, mid-turbinate swabs that can be taken by the parents themselves after receiving practical instructions from the study team are in general to be carried out on the scheduled sampling days before bringing the child to the daycare centre. Before performing sample collection for the first time, parents will receive detailed explanations in the daycare centre. On the first sampling day, sample collection will be carried out within the premises of the daycare centre and will be supervised by medically trained staff. Parents will be provided with written instructions, including an emergency telephone number. In case parents are unable or unwilling to take the mid-turbinate swabs themselves, medical staff will be present in the daycare centre on sampling days.

6 DATA MANAGEMENT

6.1 Source data and source material

Data for the study will be obtained from participant lists, from paper based and electronic questionnaires and from sampling material.

Recruitment/ individual course of study

Initial measurement

The central study office will provide all participating daycare centers with the information material relevant to the assigned study module, the written participant documents (informed consent forms, initial questionnaire) and sealable envelopes pre-addressed to the central study office to be used by the parents/guardians to return the documents.

The participating daycare centers will collect the envelopes with the informed consent forms and the initial questionnaires returned by the parents/guardians and forward all documents to the study team. The central study office will check and archive the documents, create participant lists per daycare centre and assign a unique participant number to each participant (pseudonymisation).

The data pertaining to the study (initial questionnaire and follow-up questionnaires) will be stored in pseudonymised form in the EDC (Electronic Data Capture) system REDCap®.

The EDC system will be hosted on servers belonging to the University of Würzburg and administered by the University of Würzburg's computing centre. The security policy conforms to the University of Würzburg standard. Access to the stored data will be granted via the EDC system's role and rights management system.

Follow-up questionnaires after 1, 6 and 12 weeks

In preparation for the follow-up questionnaires at three pre-defined follow-up times (1, 6 and 12 weeks) implemented in the EDC system REDCap®, participants will be asked to provide their e-mail address in the informed consent form for participation in the study. Participants who provide a valid e-mail address will receive their personal access data (pseudonym and password) by e-mail. With this access data, they can verify their access to the EDC system and subsequently fill in the follow-up questionnaire using eCRF.

Parents will be provided with pre-labelled test tubes and other material for the mid-turbinate swabs and saliva samples taken at home. The test tubes handed in by the parents at the daycare centre will be collected by members of the study team and taken to the diagnostic laboratory.

All other mid-turbinate swabs as well as all oropharyngeal swabs and blood samples will be carried out by medically trained staff, labelled and documented, and the samples will be forwarded to the diagnostic laboratory at the Institute of Hygiene and Microbiology or the Institute of Virology at the University of Würzburg for analysis.

The laboratory samples will be labelled with the name and date of birth of the participant in order to allow for the necessary timely notification of both the participant (parents/guardians) and the local health authorities in the event of a positive test result for SARS-CoV-2.

In addition, the study team members on site at the child care centers will document the attendance status of participating children (present / planned absence (e.g. holiday) / unexpected absence (e.g. due to illness with/without respiratory/gastrointestinal symptoms) and the status regarding successful sample collection (mid-turbinate swab or saliva sample). This documentation will be forwarded to the central study office.

Children aged three years and above will be interviewed within the daycare centre after 2, 6 and 12 weeks by members of the study team using a paper-based questionnaire. The data will be pseudonymised in the central study office, entered into the RedCap database and forwarded to the Institute for Clinical Epidemiology and Biometry for analysis.

Data on the acceptance of the measures by parents and by the staff at the daycare centre will be gathered both with quantitative methods using questionnaires for all participants and with qualitative methods using semi-structured guideline interviews in all groups of participants (after obtaining separate informed consent). The interviews will be carried out by members of the Department for General Medicine in cooperation with the Department of Child and Adolescent Psychiatry, Psychosomatics and Psychotherapy.

In addition, questionnaires will be used to gather basic data on characteristics of the daycare centers (group sizes, number of childcare workers per group) and infection data at the level of the daycare centers (number of cases per participant (child/childcare worker) at the centre). The questionnaires will be sent to the central study office and the data will be forwarded to the Institute for Clinical Epidemiology and Biometry for analysis.

Data on the number of absent days per daycare centre and week will be documented in anonymous form per group by the staff at the daycare centre, sent to the central study office and forwarded to the Institute for Clinical Epidemiology and Biometry for analysis.

On sampling days, the number of staff and the time required for sample collection and documentation will be recorded both for study team members and for staff of the daycare centre. In order to determine the material costs and testing costs, the number and type of samples collected will also be recorded. If sampling is performed at home by the parents, they will be asked to document the time required for sample collection and labelling.

6.2 Study database

Management of participant documents will be performed centrally at the central study office. Only pseudonymised data will be recorded (central study office at the Institute of Hygiene and Microbiology, electronic questionnaires at the Institute of Epidemiology and Biometry).

Sample material will be analysed at the Institute of Hygiene and Microbiology and the Institute of Virology and the results will be entered in Excel databases. The results will be transferred to the central study office using a password protected exchange directory restricted to this study and will be forwarded to the Institute for Clinical Epidemiology and Biometry in pseudonymised form for analysis.

For further analyses, the Institute for Epidemiology and Biometry will merge the data gathered in the questionnaires with the laboratory results in pseudonymised form using the participant number.

All data obtained in the process of the study and for analysis purposes will be stored in two separate systems. Every participant will be assigned a unique participant number (pseudonym) and all questionnaires and interviews will use only this pseudonym and no information relating to an identified natural person. The research data can be linked with the information relating to an identified natural person by the de-identification list in conjunction with the pseudonym. During the process of data linkage, the research data will be classed as "information relating to an identifiable natural person" and the applicable data protection regulations will be binding. For these purposes, the central data processing unit (Institute for Clinical Epidemiology and Biometry / ICE-B) will programme a separate data base for the storage of contact details, e-mail addresses and the respective pseudonyms.

6.3 Laboratory methods

6.3.1 Virus detection

Polymerase chain reaction (PCR)

6.3.2 Antibody detection

Enzyme-linked immunosorbent assay (ELISA), point of care test (lateral-flow ELISA)

6.4 Sampling

6.4.1 Mid-turbinate swabs

Mid-turbinate swabs will be performed as mid-turbinate swabs, to be carried out by tilting the child's head back slightly, inserting the swab frontally into a nostril and rotating the swab three times.

6.4.2 Saliva samples

The parents will be provided with pre-labelled test tubes (50 ml) for taking saliva samples. The test tubes will either be filled directly with saliva or by using the following procedure: After filling the test tube with 10ml of still water or tap water, the parents let the child rinse their mouth with the solution and spit it back into the test tube.

6.4.3 Blood tests

Blood samples will be taken using finger-prick testing with single-use safety lancets. After disinfecting and pricking the fingertip, a few drops of blood will be drawn into a capillary tube for performing the point of care test.

In the event of a positive or borderline result (approximately 1% of test results), parents/guardians will be given the option of additional venous blood sampling (1ml, in accordance with WHO guidelines [29]), which will be carried out by a physician under the supervision of a pediatrician if written consent is obtained.

7 BIOMETRICAL ASPECTS

7.1 Endpoints

Primary endpoints for the different surveillance measures

- [Modules 1-3]: Rate of acceptance of the respective surveillance protocol defined as: proportion of children/childcare workers with “successful“ participation in sample collection (mid-turbinate swab or saliva sample) among all children and childcare workers in the daycare centre. Parent/child groups and childcare workers who do not consent to participation in the study will be included in the calculations as “non-successful” participants. Each study participant will be classed as “successful“ with respect to sample collection if at least 60% of all scheduled samples were collected successfully.

  A surveillance protocol will be classed as “successful“ if the rate of acceptance exceeds the following level:

  ≥30% (Module 1: intensive monitoring / mid-turbinate swabs)

  ≥37.5% (Module 2: extensive monitoring / mid-turbinate swabs)

  ≥37.5% (Module 3: intensive monitoring / saliva samples)

- [Module 4]:
  Proportion of cases with successful sample collection (i.e. completion of sample collection and diagnostic analysis – or receipt of test result in the case of external testing – within 72h after first telephone contact with hotline) among all cases of sample collection recommended for symptomatic children, symptomatic childcare workers or symptomatic household members of children/childcare workers

  The surveillance protocol will be classed as “successful“ if the rate of acceptance exceeds the following level:

  ≥70% (Module 4: symptom-based monitoring)

Secondary endpoints for the different surveillance measures

- Comparison of initial rate of consent between the groups [Modules 1-4]
- Comparison of rate of acceptance between the groups [Modules 1-3]

Secondary endpoints regarding acceptance by parent/child groups

- Drop out rate of parents (withdrawal of initial consent), reasons for withdrawal of consent
- Drop out rate of children (proportion of scheduled samples refused), overall and stratified by age groups
- Drop out rate over time (weekly, cumulative)

Secondary endpoints regarding acceptance by childcare workers

- Initial rate of consent of childcare workers (proportion of childcare workers with signed informed consent forms among all childcare workers contacted)

Secondary endpoints regarding acceptance by daycare centers

- Initial rate of consent of contacted daycare centers to study participation (proportion of centers consenting to participation among all daycare centers contacted)

Secondary endpoints regarding acceptance of seroprevalence testing

- Acceptance of (finger-prick) blood sampling stratified by children and childcare workers (proportion of all scheduled finger-prick blood samples performed successfully)

Exploratory analyses of potential predictors of acceptance

- Association between initial consent / drop out regarding respiratory sample collection and:
  - age/ sex of child
  - family/household size
  - employment status, educational level
  - assigned screening module
  - personal assessment of the dangers of a SARS-CoV-2 infection
  - personal opinions on the necessity of social restrictions in response to SARS-CoV-2 infections
  - personal experience of SARS-CoV-2 (e.g. cases of disease or death in family or among friends and acquaintances)
  - personal opinions on child vaccinations

Exploratory Analyses regarding psychosocial effects: Data collection using survey questionnaires related in terms of content to the coronavirus contact tracing app ("Corona-Warn-App") published by the Robert Koch Institute and additional qualitative interviews at the beginning and end of the intervention

- Parents' satisfaction with the screening measure (weeks 1, 6, 12)
- Parents' sense of security due to the screening measure (weeks 1, 6, 12)
- Children's satisfaction (increase/decrease of anxiety, crying, defensive movements, refusal to attend daycare centre) (weeks 1, 6, 12)
- Subjective stress level caused by screening measure (weeks 1, 6, 12)
- Negative effects of screening measure on family climate (weeks 1, 6, 12)
- Organisation of daily routines affected by screening measure (weeks 1, 6 12)
- Occurrence of effects personally perceived as negative consequences of the screening measures (e.g. quarantine period following a positive SARS-CoV-2 test) (weeks 1, 6, 12)
- Mental health symptoms according to validated questionnaires (weeks 1, 6, 12)

Qualitative Interviews: in-depth exploration of study participants' expectations and opinions before and after implementation of the intervention

- Before the intervention: Participants' expectations, wishes and reservations regarding the measures to be introduced
- After the intervention: Personal experience of the measures, reasons for acceptance or lack of acceptance, potential barriers and obstacles to implementation

Children interviews after implementation of the intervention to determine acceptance (children questionnaire)

- Children's acceptance (according to 3 questions); interview and documentation directly after sample collection (after 2, 6 and 12 weeks)

Exploratory Analyses regarding effectiveness of the intervention

- Proportion of all mid-turbinate swabs tested positive for SARS-CoV-2 (PCR)
- Proportion of all saliva samples tested positive for SARS-CoV-2 (PCR)
- Proportion of parents/siblings/others among all persons tested for SARS-CoV-2 (PCR) [Module 4]
- Proportion of absent days due to respiratory symptoms
- Proportion of absent days due to SARS-CoV2 infection of child and/or SARS-Cov-2 infection of a family member/other contact person (quarantine)
- Closures of daycare groups / daycare centers by the health authorities due to positive SARS-CoV-2 results within the research study
- Closures of daycare groups / daycare centers by the health authorities due to other causes
- Proportion of children and childcare workers with positive serological test result (antibodies) before and after the observation period of 12 weeks

Exploratory Analyses regarding cost expenditure incurred by the intervention

- Average costs per sample collection and index child, per week
- Average daily staff time required for study purposes per week (daily documentation of time estimated by physicians, helpers, childcare workers, parents (weeks 1, 6, 12))
- Average costs per detected case of SARS-CoV-2 infection (number and costs of tests carried out for the detection of one SARS-Cov-2 infection)
- Average number of absent days of children with potential COVID-19 symptoms (respiratory/ gastrointestinal symptoms)

An additional evaluation of acceptance will be carried out at the end of the study period based on the results of qualitative, semi-structured interviews in a subsample of participants.

Exploratory Analyses regarding the proportion of other viral causes of respiratory symptoms

- Detectable proportion of other respiratory viruses (e.g. rhinovirus, adenovirus, etc.) in respiratory secretions of children attending daycare centers during the SARS-CoV-2 pandemic

7.2 Data analysis methods

The primary endpoints will be estimated as 95% confidence intervals using the Wilson score method. Due to the purely exploratory nature of all analyses and the fact that the four modules will be implemented in separate daycare centers, the significance level will not be adjusted for multiple testing and all analysis results will be interpreted with the necessary caution. The significance level will be set to 5% for all analyses. In a first step, descriptive analyses

(frequency (percent), mean (SD) or median (IQR)) will be reported for all endpoints. The analysis of the secondary endpoints drop out rate, initial rate of consent, acceptance of blood sampling (stratified by childcare workers and parents), proportion of positive serological tests and proportion of positive PCR tests will, in a first step, compare all four groups using test procedures such as the Chi-squared-test, ANOVA or the Kruskal Wallis test as appropriate. Subsequently, pairwise comparisons will be carried out using appropriate post-hoc tests. The rate of acceptance will first be compared between all modules 1 to 3 using the chi-squared test. Analysis of the development of the psychosocial factors over time will be stratified by monitoring module and will use tests for repeated measurements such as the McNemar test, Repeated Measurements ANOVA and the Friedman test. In addition, baseline values and the follow-up values after 12 weeks will be compared between modules using appropriate tests such as the chi-squared test and ANOVA or non-parametric tests followed by post-hoc tests. Potential predictors of the rate of acceptance will be analysed (overall for all modules combined and stratified by module) in univariable analyses using the chi-squared test and, if a sufficient sample size is obtained, additionally using multivariable logistic regression. The analysis of costs, the proportion of absent days and data on the closure of daycare groups will be restricted to descriptive methods. The development of seroprevalence data over time will be reported using descriptive methods, possibly including graphical displays. The results of the children interviews will be reported descriptively using frequencies. If possible, an ICC value (intraclass correlation) for the daycare centers will be calculated as a basis for sample size estimation for a future cluster randomised study. All analyses will be performed in SAS, R or SPSS.

7.3 Populations for analysis

The main analysis will include the data of all participants.

7.4 Timing of analysis

The study data will not be analysed until data collection has been completed in all study centers. No interim analyses will be carried out.

7.5 Sample size discussion and power analysis

Due to the study design as a feasibility study, the primary endpoint for modules 1-3 is the rate of acceptance of the respective surveillance protocol, defined as the proportion of children and childcare workers with successful participation in sample collection (mid-turbinate swab or saliva sample) among all children and childcare workers in the daycare centre. Parent/child groups and childcare workers who do not consent to participation in the study will be included in the calculations as “non-successful” participants. Each study participant will be classed as “successful“ with respect to sampling if at least 60% of all scheduled samples were collected successfully. All persons with a positive serological test for SARS-CoV-2 will be classed as “successful” with regard to the primary endpoint as no further sample collection (mid-turbinate swabs or saliva samples) is to be carried out in these cases according to the surveillance protocol.

As the maximum achievable sample size will depend on the number of children and number of childcare workers per centre, it is only possible to state a minimum sample size per module. The estimates of precision will be based on this minimum sample size. If the sample size is greater than expected, the width of the precision estimate will be reduced, allowing a more precise estimation.

The inclusion criteria result in the following minimum sample sizes (number of children and childcare workers) per module:

Module 1 (intensive monitoring / mid-turbinate swabs): 105

Module 2 (extensive monitoring / mid-turbinate swabs): 105

Module 3 (intensive monitoring / saliva samples): 185

Module 4: 275

We assume that the rate of acceptance will depend on the frequency of sampling and the type of sample collection. Based on this assumption, we expect a lower rate of acceptance in module 1 (mid-turbinate swabs twice a week) than in module 2 or 3. We therefore assume the following rates of acceptance:

Module 1 (intensive monitoring / mid-turbinate swabs): 30%

Module 2 (extensive monitoring / mid-turbinate swabs): 37.5%

Module 3 (intensive monitoring / saliva samples): 37.5%

Reasoning for sample size in module 1 (intensive monitoring / mid-turbinate swabs):

A minimum number of 100 children and 5 childcare workers is sufficient to estimate a rate of acceptance of 30% with a precision (half the width of the 95%-CI using the Wilson score method) of max. 9.1%.

Reasoning for sample size in module 2 (extensive monitoring / mid-turbinate swabs):

A minimum number of 100 children and 5 childcare workers is sufficient to estimate a rate of acceptance of 37.5% with a precision (half the width of the 95%-CI using Wilson score method) of max. 8.7%.

Reasoning for sample size in module 3 (intensive monitoring / saliva samples):

A minimum number of 175 children and 10 childcare workers is sufficient to estimate a rate of acceptance of 37.5% with a precision (half the width of the 95%-CI using the Wilson score method) of max. 6.9%.

Module 4:

The primary endpoint for module 4 is the proportion of cases with successful sample collection (i.e. completion of sample collection and diagnostic analysis – or receipt of test result in the case of external testing – within 72h after first telephone contact with hotline) among all cases relevant for sample collection. Testing will be carried out for all symptomatic parents, symptomatic children or childcare workers and further symptomatic household members after obtaining informed consent. The potential number of tests recommended will depend on the number of children and childcare workers in the module, the household size per family and the

proportion with acute respiratory illness (ARI). The (ARI)-physician consultation incidence in Germany varies between 400 and 2,500 per 100,000 inhabitants per week [30]. We assume that the proportion with ARI in our study will correspond to the ARI physician consultation incidence at times of high prevalence as we reckon with a large number of unrecorded cases and the research study will be carried out during a period of high prevalence. We furthermore assume that module 4 will include 250 parent/child groups plus 25 childcare workers. According to data of the German Federal Statistical Office for 2018, the average family size is 3.5 persons [31]. Based on this data, the following expected number of recommended tests is derived:

number of recommended tests = household size * (children+childcare workers) * proportion with acute respiratory illness per week * observation period in weeks

=3.5*275*0.025 *12= 279

We assume that 70% of all sample collections planned will be successful. A number of 279 recommended tests will be sufficient to estimate a 70% proportion of successful sample collections with a precision (half the width of the 95%-CI using the Wilson score method) of 5.4%.

Sample size for the qualitative interviews:

For the interviews with parents/guardians, we expect a number of approximately 10 participants per study arm, who shall be recruited from all participating daycare centers (module 1/2, module 3, module 4: 25-30). Overall, we expect 50-60 interviews to be carried out with parents/guardians (counting interviews at the beginning and at the intervention). For the interviews with childcare workers, candidates will be recruited from all participating daycare centers and we expect approximately 20 participants for the first round of interviews and 20 for the final interviews. The total number of interviews is therefore expected to lie in the range of 80-100.

8 QUALITY ASSURANCE AND QUALITY CONTROL

All data collection will be performed only by authorised study personnel after receiving study-specific training and will be carried out in accordance with the standard operating procedures specified for this study and all applicable data protection regulations.

Quality assurance and quality control

Quality assurance for the data entry of paper questionnaires will require either double data entry with subsequent checks for discrepancies or single entry with subsequent control checks in a 10% sample of data according to a pre-defined procedure. Automated plausibility checks of the raw data will be carried out before beginning the electronic processing of the data. The programming of electronic questionnaires will include plausibility checks and checks for missing values in core data directly during data entry. Automatic reminders will be generated if electronic questionnaires are not submitted.

All statistical analyses will be carried out by appropriately qualified staff of the University / University Hospital according to a pre-defined statistical analysis plan.

Sample handling, processing and diagnostic analysis of laboratory samples will be subject to the relevant regulations of the institutions in charge.

The study results will be submitted for publication in a peer-reviewed medical journal (external review).

Data protection

All study data will be treated strictly confidentially and will be accessible only for staff of the University/University Hospital Würzburg directly involved in the study. Appropriate measures for ensuring adherence to data protection principles at participating external institutions will be installed.

For data protection reasons, only pseudonymised data (based on the assignment of unique participant numbers) will be recorded and processed electronically (with the exception of laboratory samples up to the time of diagnosis). Neither questionnaires nor the recordings and documentation of semi-structured interviews will gather any data that could be used for directly identifying individuals (e.g. name, date of birth, address).

To allow laboratory results to be linked with questionnaire data in pseudonymised form a password protected exchange directory will be installed on the (firewall-protected) server of the University/University Hospital, which will be accessible only for directly involved study personnel.

All data obtained in the process of the study and for analysis purposes will be stored in two separate systems. Every participant will be assigned a unique participant number (pseudonym) and all questionnaires and interviews will use only this pseudonym and no information relating to an identified natural person. The research data can be linked with the information relating to an identified natural person by the de-identification list in conjunction with the pseudonym. During the process of data linkage, the research data will be classed as “information relating to an identifiable natural person” and the applicable data protection regulations will be binding. For these purposes, the central data processing unit (Institute for Clinical Epidemiology and

Biometry / ICE-B) will programme a separate data base for the storage of contact details, e-mail addresses and the respective pseudonyms. This data base will be stored in a network folder with restricted access at the "Servicezentrum Medizin-Informatik (SMI)" (service centre medical informatics) of the University Hospital Würzburg (UKW). The data stored here will not be linked with the research data at any time. All personal data in this data base will be accessible only to those members of the study team who are responsible for sending e-mails and are bound by medical confidentiality rules and/or by the obligation to observe data privacy in accordance with the EU GDPR and regional legislation. The data will in no event be passed on to third parties. After completion of the study, all personal data will be destroyed upon expiry of the prescribed period.

8.1 Date flow chart / responsibilities

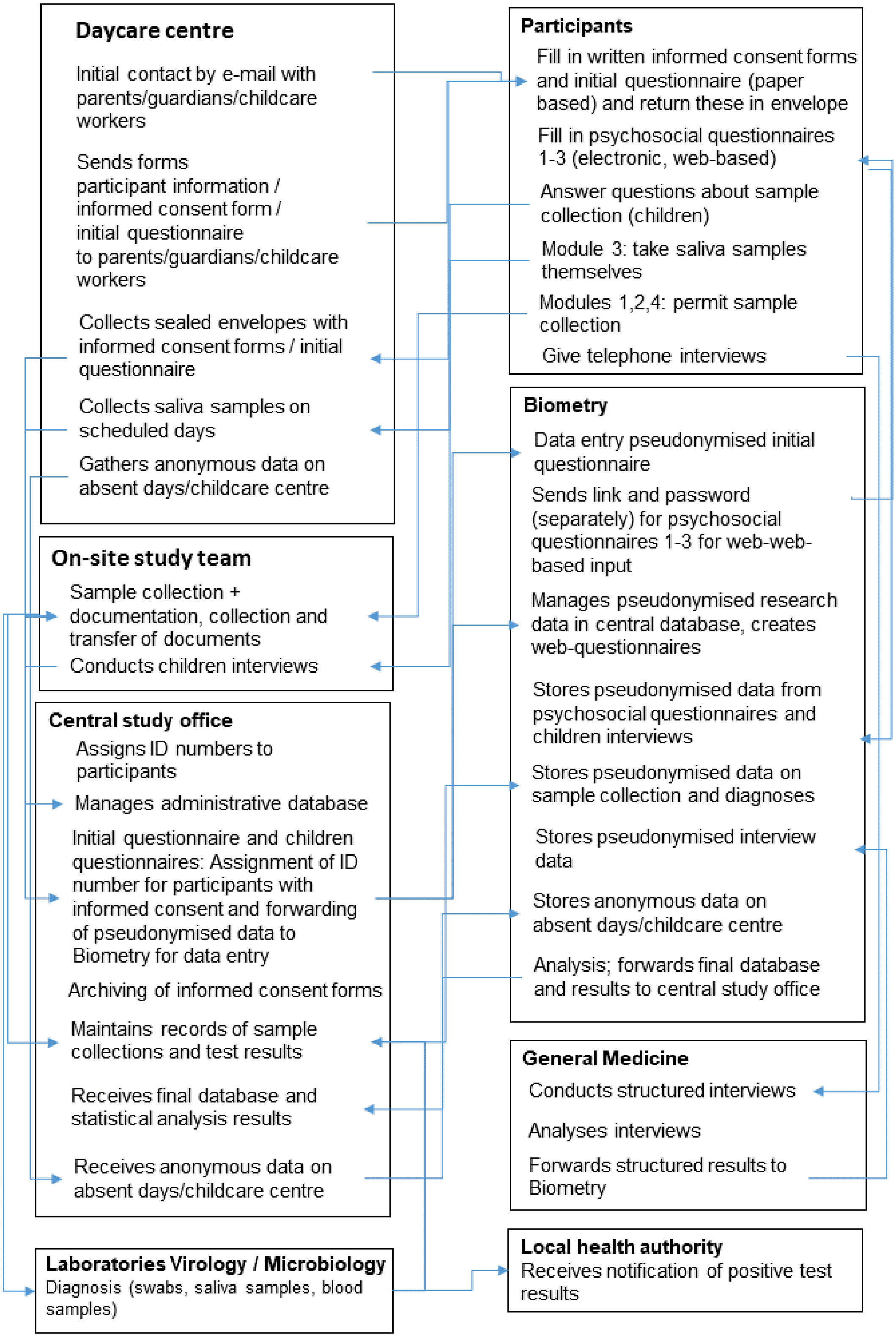

9 PUBLICATION OF RESULTS

After completion of the study, timely publication in a medical journal will be sought. Preliminary results will not be passed on to the media.

10 INSURANCE COVER FOR PARTICIPANTS

The sample collection procedures carried out during the study (mid-turbinate swabs, saliva samples, blood samples) are to be classed as study related, not medically indicated "interventions" with a low risk profile. Before the start of sample collection, appropriate insurance cover, i.e. insurance cover for participants of clinical trials not subject to mandatory insurance coverage ("Probandenversicherung für nicht der Versicherungspflicht (nach AMG/MPG) unterliegende klinische Prüfungen"), will be obtained, based on the existing framework contract between the legal department of the University Hospital of Würzburg (UKW) and the insurer HDI Gerling. The sum insured will most likely be set at max. 250,000 EUR per participant, with a maximum limit of 5,000,000 EUR for all insured events in the study (cf. general conditions of insurance for clinical trials not subject to mandatory insurance coverage / annual contract ("Allgemeine Versicherungsbedingungen für nicht der Versicherungspflicht unterliegende klinische Prüfungen / Jahresvertrag"), AVB-Prob/NV-JV, Form. 404 U205).

11 ETHICAL CONSIDERATIONS

11.1 Ethics committee approval

Before the beginning of the study, ethics committee approval will be sought at the ethics committee of the medical faculty at the University of Würzburg. Implementation of the study will not begin before ethics committee approval has been obtained.

11.2 Participant information and informed consent to sample collection

Participants will receive information on the study, including details on the voluntary nature of participation in the study, on the possibility to withdraw consent (at any time) and on potential risks, in written participant information sheets. In addition, participants will receive information in person prior to any sample collection.

Written informed consent will be obtained from both parents/guardians (for participating children), from childcare workers and from other participants (module 4) in an informed consent form.

Informed consent will be obtained separately for:

- respiratory sample collection
- blood sampling

If required, additional informed consent forms will be provided in English, Turkish, Arabic and Russian.

11.3 Informed consent to study participation

Written informed consent will be obtained from both parents/guardians (for participating children), from childcare workers and from other participants (module 4) in an informed consent form after providing participants with relevant information in person and in writing.

Informed consent will be obtained separately for:

- initial questionnaire
- further questionnaires (e.g. psychosocial questionnaires in the course of the study, children interviews)
- semi-structured interviews (sample of participants)

If required, additional informed consent forms will be provided in English, Turkish, Arabic and Russian.

11.4 Data use, storage and disclosure

All participant data will be collected and used only for the purposes stated in the study protocol. All data collection and data administration tasks will be carried out only by authorised study personnel in accordance with all applicable data protection regulations.

The transfer of study data and samples from participating daycare centers to the central study office will be carried out only by authorised study personnel in accordance with SOPs specified in advance.

Only pseudonymised data will be recorded and stored electronically. In order to ensure data security, all storage and processing of data will be performed using the technical facilities of the University/University Hospital only, on password protected devices and within the premises of the University/University Hospital.

Any transfer and exchange of data will be limited to personally named members of the study team and staff at institutions of the University of Würzburg and the University Hospital Würzburg (UKW) directly involved in the study, and will be performed using a password protected exchange directory installed specifically for this study. No data will be disclosed to third parties.

Only aggregated data permitting neither direct nor indirect identification of individual persons/households will be published.

All study documents will be stored and archived in accordance with legal provisions and will be destroyed upon expiry of the prescribed period.

12 REFERENCES

1. Bertoncelli D, Guidarini M, Della Greca A, et al. COVID19: potential cardiovascular issues in pediatric patients. *Acta bio-medica : Atenei Parmensis.* 2020;91(2):177-183.
2. Robert Koch-Institut. SARS-CoV-2 Steckbrief zur Coronavirus-Krankheit-2019 (COVID-19). 2020; https://www.rki.de/DE/Content/InfAZ/N/Neuartiges_Coronavirus/Steckbrief.html#doc13776792bodyText2. Accessed 22/07/2020.
3. Streng A, Hartmann K, Armann J, Berner R, Liese JG. [COVID-19 in hospitalized children and adolescents]. *Monatsschr Kinderheilkd.* 2020:1-12.
4. World Health Organisation. Pandemie der Coronavirus-Krankheit (COVID-19). 2020; https://www.euro.who.int/de/health-topics/health-emergencies/coronavirus-covid-19/novel-coronavirus-2019-ncov.
5. Deutsche Akademie für Kinder- und Jugendmedizin. Stellungnahme der Deutschen Akademie für Kinder- und Jugendmedizin e.V. zu weiteren Einschränkungen der Lebensbedingungen von Kindern und Jugendlichen in der Pandemie mit dem neuen Coronavirus (SARS-CoV-2). 2020; https://www.dakj.de/stellungnahmen/stellungnahme-der-deutschen-akademie-fuer-kinder-und-jugendmedizin-e-v-zu-weiteren-einschraenkungen-der-lebensbedingungen-von-kindern-und-jugendlichen-in-der-pandemie-mit-dem-neuen-coronavirus-sar/. Accessed 07/18/2020.
6. Schober T, Rack-Hoch A, Kern A, von Both U, Hübner J. Coronakrise: Kinder haben das Recht auf Bildung. *Dtsch Arztebl International.* 2020;117(19):990-994.
7. Panahi L, Amiri M, Pouy S. Clinical Characteristics of COVID-19 Infection in Newborns and Pediatrics: A Systematic Review. *Archives of academic emergency medicine.* 2020;8(1):e50.
8. Ludvigsson JF. Systematic review of COVID-19 in children shows milder cases and a better prognosis than adults. *Acta Paediatr.* 2020;109(6):1088-1095.
9. Alunno A, Carubbi F, Rodríguez-Carrio J. Storm, typhoon, cyclone or hurricane in patients with COVID-19? Beware of the same storm that has a different origin. *RMD open.* 2020;6(1).
10. Verdoni L, Mazza A, Gervasoni A, et al. An outbreak of severe Kawasaki-like disease at the Italian epicentre of the SARS-CoV-2 epidemic: an observational cohort study. *Lancet.* 2020;395(10239):1771-1778.
11. Garazzino S, Montagnani C, Donà D, et al. Multicentre Italian study of SARS-CoV-2 infection in children and adolescents, preliminary data as at 10 April 2020. *Euro surveillance : bulletin Europeen sur les maladies transmissibles = European communicable disease bulletin.* 2020;25(18).
12. Robert Koch Institut. COVID-19: Fallzahlen in Deutschland und weltweit. 2020; https://www.rki.de/DE/Content/InfAZ/N/Neuartiges_Coronavirus/Fallzahlen.html. Accessed 22/07/2020.
13. Danis K, Epaulard O, Bénet T, et al. Cluster of coronavirus disease 2019 (Covid-19) in the French Alps, 2020. *Clin Infect Dis.* 2020.
14. National Centre for Immunisation Research and Surveillance (NCIRS). COVID-19 in schools – the experience in NSW. 2020; http:// ncirs. org. au/ sites/default/ files/ 2020- 04/ NCIRS% 20NSW% 20Schools%20COVID_ Summary_ FINAL% 20public_ 26% 20April%202020. pdf.
15. Gudbjartsson DF, Helgason A, Jonsson H, et al. Spread of SARS-CoV-2 in the Icelandic Population. *New England Journal of Medicine.* 2020.
16. Lavezzo E, Franchin E, Ciavarella C, et al. Suppression of COVID-19 outbreak in the municipality of Vo, Italy. *medRxiv.* 2020.
17. Mizumoto K, Omori R, Nishiura H. Age specificity of cases and attack rate of novel coronavirus disease (COVID-19). *medRxiv.* 2020.
18. Viner RM, Russell SJ, Croker H, et al. School closure and management practices during coronavirus outbreaks including COVID-19: a rapid systematic review. *The Lancet Child & adolescent health.* 2020;4(5):397-404.

19. Bi Q, Wu Y, Mei S, et al. Epidemiology and transmission of COVID-19 in 391 cases and 1286 of their close contacts in Shenzhen, China: a retrospective cohort study. *The Lancet Infectious diseases.* 2020.
20. Fontanet A, Tondeur L, Madec Y, et al. Cluster of COVID-19 in northern France: A retrospective closed cohort study. *medRxiv.* 2020.
21. Terry C. Jones BM, Talitha Veith, Marta Zuchowski, Jörg Hofmann, Angela Stein, Anke Edelmann, Victor Max Corman, Christian Drosten. An analysis of SARS-CoV-2 viral load by patient age. 2020; .
22. Brauner JM, Mindermann S, Sharma M, et al. The effectiveness and perceived burden of nonpharmaceutical interventions against COVID-19 transmission: a modelling study with 41 countries. *medRxiv.* 2020.
23. Achenbach TM. *The Achenbach system of empirically based assessment (ASEBA): Development, findings, theory, and applications.* University of Vermont, Research Center for Children, Youth, & Families; 2009.
24. EuroQol. EuroQol--a new facility for the measurement of health-related quality of life. *Health policy (Amsterdam, Netherlands).* 1990;16(3):199-208.
25. Löwe B, Wahl I, Rose M, et al. A 4-item measure of depression and anxiety: validation and standardization of the Patient Health Questionnaire-4 (PHQ-4) in the general population. *Journal of affective disorders.* 2010;122(1-2):86-95.
26. Kuckartz U. *Qualitative Inhaltsanalyse: Methoden, Praxis, Computerunterstützung.* Weinheim: Beltz-Juventa; 2012.
27. Moser A, Korstjens I. Series: Practical guidance to qualitative research. Part 3: Sampling, data collection and analysis. *European Journal of General Practice.* 2018;24(1):9-18.
28. Guest G, Bunce A, Johnson L. How Many Interviews Are Enough?: An Experiment with Data Saturation and Variability. *Field Methods.* 2006;18(1):59-82.
29. World Health Organization. *WHO guidelines on drawing blood: best practices in phlebotomy.* World Health Organization; 2010.
30. Köpke K, Prahm K, Buda S, Haas W. Evaluation einer ICD-10-basierten elektronischen Surveillance akuter respiratorischer Erkrankungen (SEEDARE) in Deutschland. *Bundesgesundheitsblatt - Gesundheitsforschung - Gesundheitsschutz.* 2016;59(11):1484-1491.
31. Statistische Bundesamt. Familien und Familienmitglieder nach Bundesländern. 2020; .

13 APPENDIX

13.1 Participant information sheets

- *Module 1: Information sheets for parents/guardians and childcare workers*
- *Module 2: Information sheets for parents/guardians and childcare workers*
- *Module 3: Information sheets for parents/guardians and childcare workers*
- *Module 4: Information sheets for household members of children and childcare workers at the daycare centre*

13.2 Informed consent forms

- *Module 1: Informed consent forms for parents/guardians and childcare workers*
- *Module 2: Informed consent forms for parents/guardians and childcare workers*
- *Module 3: Informed consent forms for parents/guardians and childcare workers*
- *Module 4: Informed consent forms for household members of children and childcare workers at the daycare centre*

13.3 Data collection instruments

- *Initial questionnaire (basic questionnaire)*
- *Psychosocial questionnaires (weeks 1, 6, 12)*
- *Children questionnaires (weeks 2, 6, 12)*
- *Questionnaire for symptomatic persons*
- *Structured interviews (using a guideline)*
- *Documentation of sample collection intended / performed*
- *Documentation of cost factors (material costs, personnel and time requirements)*
- *Questionnaire on characteristics of the daycare centre (e.g. number of children, group sizes,* hygiene measures specific to SARS-CoV-2*; number of absent days per week due to illness of children and childcare workers)*

13.4 Additional information material

- If relevant: announcements on notice boards in the daycare centers
- If relevant: information leaflets for parents
