# Supplementary material for: Feasibility of SARS-CoV-2 Surveillance Testing Among Children and Childcare Workers at German Day Care Centers: A Nonrandomized Controlled Trial
Source: JAMA Netw Open. 2022 Jan 4;5(1):e2142057. doi: 10.1001/jamanetworkopen.2021.42057 (PMC8728621; doi:10.1001/jamanetworkopen.2021.42057)
Supplement: Supplement 2. — eAppendix 1. Supplemental Methods eAppendix 2. Supplemental Results eTable 1. Characteristics and Attitudes of Participating Parents eTable 2. Characteristics and Attitudes of Participating CCW eTable 3. Factors Associated With and Reasons for Declined Participation in Parents eTable 4. Dropout Rates in Modules 1-3 and Unsuccessful Testing in Module 4 eTable 5. Number of Samples Tested for SARS-CoV-2 in DCC With Continuous Surveillance (Modules 1-3) eTable 6. Model Parameters Used for All Simulations eTable 7. Varied Parameters and Processes in the Model eFigure 1. Flowcharts of Wue-KiTa-CoV Study eFigure 2. General and Specific Measures Implemented Against SARS-CoV-2 eFigure 3. Final Assessment of Study Measures by Parents and CCWs eFigure 4. Events Associated With Potential Introduction of SARS-CoV-2 Into a DCC eFigure 5. Modeling Results Scenario 1 eFigure 6. Simulation Results of the Virtual Infection Spread Model for Scenario 2 eFigure 7. Modeling Results Scenario 3 eFigure 8. Modeling: Effects of Longer Time to Result on Scenario 1 eFigure 9. Modeling: Effects of Longer Time to Result on Scenario 2 eFigure 10. Modeling: Effects of Longer Time to Result on Scenario 3 eReferences [file jamanetwopen-e2142057-s002.pdf]

Supplemental Online Content

Forster J, Streng A, Rudolph P, et al; Wü-KiTa-CoV-Study Group. Feasibility of SARS-CoV-2 surveillance testing among children and childcare workers at German day care centers: a nonrandomized controlled trial. *JAMA Netw Open*. 2022;5(1):e2142057. doi:10.1001/jamanetworkopen.2021.42057

eAppendix 1. Supplemental Methods

eAppendix 2. Supplemental Results

eTable 1. Characteristics and Attitudes of Participating Parents

eTable 2. Characteristics and Attitudes of Participating CCW

eTable 3. Factors Associated With and Reasons for Declined Participation in Parents

eTable 4. Dropout Rates in Modules 1-3 and Unsuccessful Testing in Module 4

eTable 5. Number of Samples Tested for SARS-CoV-2 in DCC With Continuous Surveillance (Modules 1-3)

eTable 6. Model Parameters Used for All Simulations

eTable 7. Varied Parameters and Processes in the Model

eFigure 1. Flowcharts of Wue-KiTa-CoV Study

eFigure 2. General and Specific Measures Implemented Against SARS-CoV-2

eFigure 3. Final Assessment of Study Measures by Parents and CCWs

eFigure 4. Events Associated With Potential Introduction of SARS-CoV-2 Into a DCC

eFigure 5. Modeling Results Scenario 1

eFigure 6. Simulation Results of the Virtual Infection Spread Model for Scenario 2

eFigure 7. Modeling Results Scenario 3

eFigure 8. Modeling: Effects of Longer Time to Result on Scenario 1

eFigure 9. Modeling: Effects of Longer Time to Result on Scenario 2

eFigure 10. Modeling: Effects of Longer Time to Result on Scenario 3

eReferences

This supplemental material has been provided by the authors to give readers additional information about their work.

© 2022 Forster J et al. *JAMA Network Open.*

eAppendix 1. Supplemental Methods

SARS-CoV-2 PCR and antibody testing

SARS-CoV-2 nucleic acid testing was performed using CE certified tests. For the detection of SARS-CoV-2 IgG antibodies, participants were screened by point-of-care testing (Panbio COVID-19 IgG/IgM Rapid Test, Abbott, North Chicago, USA) after fingertip prick. Positive results were verified from serum samples using SERION ELISA agile SARS-CoV-2 (Institut Virion/Serion, Wuerzburg, Germany, target: SARS-CoV-2 spike protein) and Elecsys Anti-SARS-CoV-2 (Hoffmann-La Roche AG, Basel, Switzerland, target: SARS-CoV-2 nucleocapsid) [2]. Only IgG positive samples verified by ELISAs were considered positive.

Questionnaires and interviews

Questionnaires

To identify children with chronic or special health care needs we used the Children with Special Health Care Needs-(CSHCN-)Screener. [3] The CSHCN Screener is a set of five questions to be self-administered as part of a parent/caretaker survey. The definition of a special care need (CSHCN positive screening) was based on the affirmative answer to at least one of the five main questions, including all associated sub-questions.

Key aspects of general health were measured in parents and CCW by self-report with the Minimum European Health Module (MEHM, [4]. The MEHM consists of three global questions concerning three health domains: self-perceived health, chronic conditions and long-term activity limitation.

In addition, internal symptoms (anxiety, depression) were assessed in parents, CCW and children. For children, the subscale "anxious/depressed" of the German version of the Child Behaviour Checklist 1.5-5 [5] was administered via parent-report. Symptomatology in parents and CCW was measured via self-report by the Patient Health Questionnaire-4 (PHQ-4, [6]. Its

© 2022 Forster J et al. *JAMA Network Open.*

four items are drawn from the first two items of the 'Generalized Anxiety Disorder–7 scale' (GAD–7) and the 'Patient Health Questionnaire-8' (PHQ-8).

For descriptive purposes, for both scales we set the cut-off at the 90th percentile using the original US-American norms of the CBCL 1.5-5 for both sexes and German sex-specific norms for 25 to 34 year olds of the PHQ-4. [7]

Qualitative Interviews

Semi-structured interviews were held with a subsample of parents of the children and teachers from the day care center (DCC) to explore the expectations, demands and concerns regarding the four tested modules. These data will be analysed separately.

Data analysis for the feasibility study

All variables were presented as frequencies.

The sample size was given by the number of children and CCW. Before the start of study, we assumed in module 1 a minimum sample size of 100 children and 5 CCW, which is sufficient to reach an acceptance of 30% with a precision of 9.1%, and in module 2 a minimum sample size of 100 children and 5 CCW, which is sufficient to reach an acceptance of 37.5% with a precision of 8.7%, and in module 3 a minimum sample size of 175 children and 10 CCWs, which is sufficient to reach an acceptance of 37.5% with a precision of 6.9%.

In module 4, the potential number of tests was dependent on the number of children and CCW in the participating module and the household size per participating family in the module plus the proportion with acute respiratory illness (ARI). We assumed an average family size of 3.5 persons, a proportion of 0.025 ARI per week and a sample size of 275 (250 children, 25 caregivers). [1,8]

Based on this data, the following expected number of recommended tests is derived:

Number of recommended tests = household size * (children + CCW) * proportion with ARI per week * observation period in weeks =3.5*275*0.025 *12= 279

We assume that 70% of all sample collections planned will be successful. A number of 279 recommended tests will be sufficient to estimate a 70% proportion of successful sample collections with a precision (half the width of the 95%-CI using the Wilson score method) of 5.4%.

The primary endpoints per module were estimated as 95%-confidence intervals using the Wilson score method. The significance level was set to 5% for all analysis. No adjustment for multiple testing was done, as all analysis were explorative. Descriptive statistics (frequencies (percent), median (IQR)) of baseline and follow-up data are displayed stratified by children/parents and CCW. Analysis were conducted separately for children/parents and CCW. According to the distribution of the variables, differences between two groups (participations vs. no participations, drop-out vs. no drop-outs) were tested using the $\chi2$ -test, Fisher Test, Student's t-test or Mann-Whitney U-Test.

To determine the influence of the type of respiratory surveillance in participating in the surveillance, the attitude of the parents towards SARS-CoV-2 variables and sociodemographic factors such as age, sex and school education of their children univariable and multivariable logistic regression analysis was performed for parents. The question "What are your views on the planned measures for SARS-CoV-2 testing at your day care center?" was excluded from the logistic regression, due to collinearity with other variables. Due to the large sample size, no variable selection was performed. For CCW the sample size was too small to perform a logistic regression for the participation rate. Development in psychosocial factors and attitudes towards SARS-CoV-2 over time were analysed using McNemar-Bowker Test, Cochrane Test or Friedmann Test as appropriately. Changes over time were calculated combined and stratified by study module (1-3 vs 4). Due to the small number of drop-outs no multivariable analysis for drop-outs was conducted. All analyses were performed in SAS Version 9.4 or SPSS Version 26.

In case more than one child of family participated in the study, the parent related questions were counted only once. If the questionnaire was filled out twice, randomly one was chosen.

© 2022 Forster J et al. *JAMA Network Open.*

Mathematical Model

To model the spread of infection in DCC, we considered a typical DCC consisting of N children groups $C = (C_1, \dots, C_N)$ and their corresponding CCW $T = (T_1, \dots, T_N)$ as well as the head of the DCC H. Furthermore, children groups consist of subgroups of children participating or not participating in regular testing, respectively. All individuals within the same group can interact with each other and, thus, can infect each other. Furthermore, infection transmission is also possible between individuals from different groups: Firstly, children and CCW from different groups can infect each other via the common usage of *e.g.* bathrooms. Secondly, children and the corresponding CCW can infect each other as they stay in the same room. Thirdly, also CCW from different groups and CCW and the head of the DCC can infect each other, as we assume daily meetings of CCW among each other as well as with the head for a certain duration each day in respective rooms. The group structure of this virtual DCC is depicted in the main manuscript (Figure 3A). The applied parameters are shown in eTable 6. Since the number of individuals in DCC is generally low and, thus, stochastic variation is high, we used a state-based model (SBM) to account for the stochasticity in the system. The SBM as depicted in the main manuscript (Figure 3B) shows the model states and the state transitions defined by the corresponding transition rates. For each of the aforementioned groups, this SBM is realized with group specific rates and parameters as summarized above. Each individual in the virtual DCC belongs to one model state: All individuals that can be infected are in the state *Susceptible*. They can become infected with rates $i_{si,intern}$ and $i_{si,intra}$ that account for inter- and intra-group infection, respectively. In order to calculate infection risks, we simulate for each infectious individual a unique viral load kinetics based on the piecewise linear viral load model which is used in Jones *et al.* [9] for estimating viral peaks from patient data. Under consideration of the viral load at a certain time point as well as the room size, emission, respiratory rate and contact duration for different interactions, we calculate the infection probability using the aerosol transmission and infection risk calculator in indoor environments by Lelieveld *et al.* [10] Infected individuals are either in the state ‘symptomatic infected’ or ‘asymptomatic infected’ depending on a parameter that defines the ratio of symptomatic over asymptomatic individuals.

© 2022 Forster J et al. *JAMA Network Open.*

Furthermore, individuals can obtain the states *Quarantined* or *Isolated* and recovered individuals obtain the state *Immune* and are henceforth considered to be not susceptible to infection anymore. This is an assumption we can make since we only simulate the infections spread during the first 30 days after the introduction of an index case. Infected individuals that are tested positive will always be isolated. However, transition to the state *Quarantined* depends on the implemented policy. We simulated two different quarantine policies: Firstly, quarantine policies implemented during the duration of the study, *i.e.* all group members in the same children group and the corresponding CCW will be quarantined and are only allowed to go back to the DCC after they had one negative test result after seven days or stayed in quarantine for 14 days. In the following we will refer to this policy as “regular quarantine policy”. Secondly, only positive tested individuals are isolated and all other children and CCW will remain in the DCC to maintain child care to which we will refer in the following as “limited quarantine policy”.

Besides, we used this model to predict infection spread within the DCC for various scenarios, where we varied the test participation of the children from 0% to 100%, the test frequency (No testing, 1 weekly, 5 days/week) as well as the respective test days. All different scenarios we simulated are shown in eTable 7.

Each of these scenarios was simulated 40,000 times. Furthermore, we considered three different starting conditions for the entry of the infection into the DCC: (i) the worst-case scenario with an infected asymptomatic child that does not participate in regular testing, (ii) a random scenario with an infected child that is randomly symptomatic or asymptomatic and randomly takes part in regular testing and (iii) a random scenario with an infected CCW that is randomly symptomatic or asymptomatic.

In the SBM simulations, testing of individuals is performed by PCR-testing with test quality criteria as given in eTable 6. Simulations are performed for a time duration of 30 days with a time step of one day. Thus, infection spread is simulated on a daily basis and tests are always

performed in the morning, such that test results will be available in the evening of the same day, *i.e.* these processes are realized in a fixed order in the model implementation.

Data analysis for the mathematical model

To evaluate infection spread in each simulated scenario, we calculated the average number of secondary infections (ASI) that occurred over all simulations in one scenario. Furthermore, to compare both quarantine policies we computed the probability that in the “limited quarantine scenario” more secondary infections occur than in the “regular quarantine scenario”. Under the null hypothesis that this quarantine policy does not affect the number of secondary infections, the distribution of the number of secondary infections is identical and, thus, both quarantine policies are equally good. To determine significance of the obtained probability, we computed the p-value by using a permutation testing framework, where the data from both quarantine policies are merged and 1000 new distributions are randomly drawn from the data. The p-value is then the probability that our original obtained probability is a realization of one probability obtained by the permutation testing framework. The significance is then indicated by comparing the p-value with four significance levels (**** $p<0.001$, ***$p<0.005$, **$p<0.01$, *$p<0.05$).

© 2022 Forster J et al. *JAMA Network Open.*

eAppendix 2. Supplemental Results

Additional SARS-CoV-2 testing during lockdown and in symptomatic participants in module 1-3

During the lockdown, asymptomatic testing was continued for children and CCW of module 1-3 present in DCC and 158/165 mid-turbinate swabs and 594/311 saliva samples from children/CCW were tested. SARS-CoV-2 was detected in one saliva sample of a four-year-old child (eTable 5).

Additionally to asymptomatic testing, 9 oropharyngeal swabs of 8 symptomatic participants of module 1-3 (two children, one CCW, five household members) were tested during the 12-weeks study period and 18 oropharyngeal swabs of 18 participants (four children, three CCW, 11 household members) during the lockdown. No additional SARS-CoV-2 infection was detected.

In module 4, 86 oropharyngeal swabs of 75 symptomatic participants (23 children, 13 CCW and 39 household members) were tested during the lockdown and one SARS-CoV-2 infection was detected.

Incidents that could potentially result in an introduction and outbreak of SARS-CoV-2 inside a participating DCC, were recorded as events (eFig. 4). In addition to all infections detected by study procedures, cases identified by contact tracing of participants or related to children or CCW of the participating DCCs are illustrated. Six events occurred in the 12-week study period, and six additional events during lockdown. One event (E6) was detected by continuous testing and did not result in intra-DCC transmission. Three events (E1, E4, E7) were identified by symptomatic testing and seven events (E2, E3, E5, E8, E9, E10, E11) by contact tracing. In one event (E12), the index case was a child not participating in the study and no information on the indication to test was available. Two of the events, both in module 4, resulted in outbreaks in the DDC: In E1, after introduction of SARS-CoV-2 11 secondary cases (seven children, four CCW) were detected by contact tracing inside the DCC. The affected DCC was

© 2022 Forster J et al. *JAMA Network Open.*

closed by public health authorities for 14 days. In E10, SARS-CoV-2 was detected in a symptomatic CCW not participating in the study. Among 20 children and seven CCW in the group, SARS-CoV-2 was transmitted to one child and its father.

© 2022 Forster J et al. *JAMA Network Open.*

eTable 1. Characteristics and Attitudes of Participating Parents

eTable 1a: Sociodemographic characteristics of children and parents from daycare centers screened for their willingness to participate in respiratory surveillance for SARS-CoV-2 (WueKiTaCoV Study, Germany, 2020-2021)

	N	All	N	Parents agreed to respiratory surveillance	N	Parents did not agree to respiratory surveillance	p-value*
**Children**	**635**		**440**		**195**		
Age, median (IQR)	581	3 (2-4)	442 [1]	3 (2-4)	139	3 (2-4)	0.4733
Female, n (%)	578	282 (48.8)	440	214 (48.6)	138	68 (49.3)	0.8958
Comorbidities (any), n (%)	564	51 (9)	431	35 (8.1)	133	16 (12)	0.1694
Pulmonary, n (%)	51	14 (27.5)	35	11 (31.4)	16	3 (18.8)	0.3465
Cardiovascular disease, n (%)	51	8 (15.7)	35	5 (14.3)	16	3 (18.8)	0.6842
Nervous system, n (%)	51	1 (2)	35		16	1 (6.3)	NA
Allergies, n (%)	51	20 (39.2)	35	15 (42.9)	16	5 (31.3)	0.4308
Other, n (%)	51	13 (25.5)	35	8 (22.9)	16	5 (31.3)	0.5234
German nationality, n (%)	562	538 (95.7)	429	412 (96.0)	133	126 (94.7)	0.5169
**Parents [2]**	**570**		**379**		**191**		
Age, median (IQR)	503	36 (33-40)	376	37 (33-40)	127	35 (31-39)	0.0058
Female, n (%)	507	382 (75.3)	377	276 (73.2)	130	106 (81.5)	0.0574
German nationality, n (%)	503	447 (88.9)	376	339 (90.2)	127	108 (85)	0.1127
School education, n (%)	503		373		130		<0.0001
None		1 (0.2)		1 (0.3)			
Secondary Comprehensive		29 (5.8)		10 (2.7)		19 (14.6)	
Secondary		89 (17.7)		54 (14.5)		35 (26.9)	
Grammar		377 (74.9)		303 (81.2)		74 (57.0)	
Other		7 (1.4)		5 (1.3)		2 (1.5)	
Working status, n (%)	501		371		130		0.2296
Employed / full-time		145 (28.9)		111 (29.9)		34 (26.2)	
Employed / part-time		254 (50.7)		191 (51.5)		63 (48.5)	
Self-employed		27 (5.4)		15 (4)		12 (9.2)	
Unemployed		68 (13.6)		50 (13.2)		19 (14.6)	
Other		7 (1.4)		5 (1.3)		2 (1.5)	
All adults of the household are employed, n (%)	480	386 (80.4)	358	286 (79.9)	122	100 (82)	0.6173
Importance of childcare access in daily life, n (%)	505		373		132		0.0493
Not important at all		6 (1.2)		2 (0.5)		4 (3)	
Slightly important		12 (2.4)		8 (2.1)		4 (3)	
Important		41 (8.1)		28 (7.5)		13 (9.8)	
Fairly important		104 (20.6)		85 (22.8)		19 (14.4)	
Very important		342 (67.7)		250 (67)		92 (69.7)	
Number of persons per household, median (IQR)							
All	504	4 (3-4)	372	4 (3-4)	132	4 (3-4)	0.4165
Adults	503	2 (2-2)	371	2 (2-2)	132	2 (2-2)	0.4758
Children	506	2 (1-2)	373	2 (1-2)	133	2 (1-2)	0.2960
Size of home, n (%)	506		375		131		0.0883
< 60 m²		9 (1.8)		4 (1.1)		5 (3.8)	
60-80 m²		93 (18.4)		62 (16.5)		31 (23.7)	
81-100 m²		134 (26.5)		102 (27.2)		32 (24.4)	
101-120 m²		103 (20.4)		78 (20.8)		25 (19.1)	
>120 m²		167 (33.0)		129 (34.4)		38 (29)	
Home features	508		376		132		0.9001
No garden or balcony		26 (5.1)		19 (5.1)		7 (5.3)	
Balcony / terrace		218 (42.9)		163 (43.4)		55 (41.7)	
Garden		82 (16.1)		58 (15.4)		24 (18.2)	
Garden plus balcony / terrace		182 (35.8)		136 (36.2)		46 (24.8)	

[1] Age was available from two additional children without detailed screening questionnaire [2] Questionnaire for one parent per household *Chi-square test

© 2022 Forster J et al. *JAMA Network Open.*

eTable 1b. Attitudes of parents from daycare centers screened for their willingness to participate in respiratory surveillance for SARS-CoV-2 (WueKiTaCoV Study, Germany, 2020-2021)

	N	All	N	Parents agreed to respiratory surveillance	N	Parents did not agree to respiratory surveillance	p-value*
How dangerous do you think SARS-COV-2 is for you/your family? n (%)	506		373		133		0.0002
Not dangerous		21 (4.1)		7 (1.9)		14 (10.5)	
Somewhat dangerous		97 (19.1)		80 (21.4)		17 (12.8)	
Dangerous		222 (43.9)		167 (44.8)		55 (41.4)	
Fairly dangerous		121 (23.9)		88 (23.6)		33 (24.8)	
Very dangerous		45 (8.9)		31 (8.2)		14 (10.5)	
How dangerous do you think SARS-COV-2 is for the society? n (%)	507		374		133		<0.0001
Not dangerous		10 (2)		1 (0.3)		9 (6.8)	
Somewhat dangerous		13 (2.6)		6 (1.6)		7 (5.3)	
Dangerous		118 (23.3)		83 (22.2)		35 (26.3)	
Fairly dangerous		230 (45.4)		181 (48.4)		49 (36.8)	
Very dangerous		136 (26.8)		103 (27.5)		33 (24.8)	
I personally know someone (family / friends / acquaintances) who tested positive / was ill / died due to SARS-COV-2; n (%)	509	197 (38.7)	377	156 (41.4)	132	41 (31.1)	0.0371
How important do you think restrictive measures to control SARS-CoV-2 are? n (%)	506		375		131		<0.0001
Not important at all		10 (2)		3 (0.8)		7 (5.3)	
Slightly important		13 (2.6)		7 (1.9)		6 (4.6)	
Important		59 (11.7)		34 (9.1)		25 (19.1)	
Fairly important		154 (30.4)		112 (29.9)		42 (32.1)	
Very important		270 (53.4)		219 (58.4)		51 (38.9)	
What are your views on the planned measures for SARS-CoV-2 testing at your daycare center? n (%)	500		372		128		<0.0001
Very critical		37 (7.4)		3 (0.8)		34 (26.6)	
Somewhat critical		32 (6.4)		8 (2.2)		24 (18.8)	
Neutral		93 (18.6)		52 (14)		41 (32)	
Openminded		132 (26.4)		116 (31.2)		16 (12.5)	
Very openminded		206 (41.2)		193 (51.9)		13 (10.2)	
How much do you currently feel limited in your personal life by SARS-CoV-2? n (%)	509		377		132		<0.0001
Not limited at all		17 (3.3)		11 (2.9)		6 (4.5)	
Slightly limited		104 (20.4)		86 (22.8)		18 (13.6)	
Limited		203 (39.9)		162 (43)		41 (31.1)	
Fairly limited		142 (27.9)		98 (26)		44 (33.3)	
Very limited		43 (8.4)		20 (5.3)		23 (17.4)	
Assuming a vaccine is discovered, would you be willing to be vaccinated against SARS-CoV-2? n (%)	509		376		133		<0.0001
Yes		256 (50.3)		220 (58.5)		36 (27.1)	
No		60 (11.8)		23 (6.1)		37 (27.8)	
Don't know		193 (37.9)		133 (35.4)		60 (45.1)	
How important do you consider vaccinations in general? n (%)	510		377		133		<0.0001
Not important at all		12 (2.4)		2 (0.5)		10 (7.5)	
Slightly important		6 (1.2)		2 (0.5)		4 (3)	
Important		37 (7.3)		14 (3.7)		23 (17.3)	
Fairly important		83 (16.3)		58 (15.4)		25 (18.8)	
Very important		372 (72.9)		301 (79.8)		71 (53.4)	

*Chi-square test

© 2022 Forster J et al. *JAMA Network Open.*

eTable 2. Characteristics and Attitudes of Participating CCW

eTable 2a. Socio-demographic characteristics of CCW from daycare centers screened for their willingness to participate in respiratory surveillance for SARS-CoV-2 (WueKiTaCoV Study, Germany, 2020-2021)

	N	All	N	CCW agreed to respiratory surveillance	N	CCW did not agree to respiratory surveillance
**CCW**	**157**		**150**		**7**	
Age, median (IQR)	147	29 (25-44)	142	29 (25-44)	5	34 (21-45)
Female, n (%)	147	134 (91.2)	142	129 (90.8)	5	5 (100)
German nationality, n (%)	132	131 (99.2)	128	127 (99.2)	4	4 (100)
School education, n (%)	134		130		4	
Secondary Comprehensive		14 (10.4)		13 (10)		1 (25)
Secondary		51 (38.1)		49 (37.7)		2 (50)
Grammar		68 (50.1)		67 (51.5)		1 (25)
Other		1 (0.7)		1 (0.8)		0 (-)
Working status, n (%)	139		134		5	
Employed / full-time		93 (66.9)		89 (66.4)		4 (80)
Employed / part-time		43 (30.9)		43 (32.1)		0 (-)
Self-employed		1 (0.7)		0 (-)		1 (20)
Unemployed		1 (0.7)		1 (0.7)		0 (-)
Other		1 (0.7)		1 (0.7)		0 (-)
Importance of childcare access in daily life, n (%)	56		53		3	
Not important at all		27 (48.2)		25 (47.2)		2 (66.7)
Slightly important		0 (-)		0 (-)		0 (-)
Important		8 (14.3)		7 (13.2)		1 (33.3)
Fairly important		2 (3.6)		2 (3.8)		0 (-)
Very important		19 (33.9)		19 (35.8)		0 (-)
Number of persons per household, median (IQR)						
All	148	2 (2-4)	142	2 (2-3)	6	2.5 (2-5)
Adults	145	2 (2-2)	140	2 (2-2)	5	2 (1-2)
Children	145	0 (0-1)	140	0 (0-1)	5	1 (1-2)
Size of home, n (%)	148		143		5	
< 60 m²		18 (12.2)		17 (11.9)		1 (20)
60-80 m²		34 (23.0)		33 (23.1)		1 (20)
81-100 m²		37 (25.0)		35 (24.5)		2 (40)
101-120 m²		19 (12.8)		19 (13.3)		0 (-)
>120 m²		40 (27.0)		39 (27.3)		1 (20)
Home features	149		143		6	
No garden or balcony		23 (15.4)		23 (16.1)		0 (-)
Balcony / terrace		50 (33.6)		46 (32.2)		4 (66.7)
Garden		20 (13.4)		19 (13.3)		1 (16.7)
Garden plus balcony / terrace		56 (37.6)		55 (38.5)		1 (16.7)

© 2022 Forster J et al. *JAMA Network Open.*

eTable 2b. Attitudes of CCW from daycare centers screened for their willingness to participate in respiratory surveillance for SARS-CoV-2 (WueKiTaCoV Study, Germany, 2020-2021)

	N	All	N	CCW agreed to respiratory surveillance	N	CCW did not agree to respiratory surveillance
How dangerous do you think SARS-COV-2 is for you/your family? n (%)	147		141		6	
Not dangerous		7 (4.8)		6 (4.3)		1 (16.7)
Somewhat dangerous		16 (10.9)		14 (9.9)		2 (33.3)
Dangerous		64 (43.5)		62 (44.0)		2 (33.3)
Fairly dangerous		44 (29.9)		43 (30.5)		1 (16.7)
Very dangerous		16 (10.9)		16 (11.3)		0 (-)
How dangerous do you think SARS-COV-2 is for the society? n (%)	149		143		6	
Not dangerous		3 (2)		2 (1.4)		1 (16.7)
Somewhat dangerous		5 (4.3)		3 (2.1)		2 (33.3)
Dangerous		51 (34.2)		49 (34.3)		2 (33.3)
Fairly dangerous		60 (40.3)		59 (41.3)		1 (16.7)
Very dangerous		30 (20.1)		30 (21)		0 (-)
I personally know someone (family / friends / acquaintances) who tested positive / was ill / died due to SARS-COV-2; n (%)	149	48 (32.2)	143	46 (32.2)	6	2 (33.3)
How important do you think restrictive measures to control SARS-CoV-2 are? n (%)	148		142		6	
Not important at all		1 (0.7)		1 (0.7)		0 (-)
Slightly important		8 (5.4)		5 (3.5)		3 (50)
Important		31 (20.9)		31 (21.8)		0 (-)
Fairly important		40 (27)		37 (26.1)		3 (50)
Very important		68 (45.9)		68 (47.9)		0 (-)
What are your views on the planned measures for SARS-CoV-2 testing at your daycare center? n (%)	147		141		6	
Very critical		1 (0.7)		0 (-)		1 (16.7)
Somewhat critical		8 (5.4)		5 (3.5)		3 (50)
Neutral		18 (12.2)		18 (12.8)		0 (-)
Openminded		44 (29.9)		43 (30.5)		1 (16.7)
Very openminded		76 (51.7)		75 (53.2)		1 (16.7)
How much do you currently feel limited in your personal life by SARS-CoV-2? n (%)	149		143		6	
Not limited at all		1 (0.7)		1 (0.7)		0 (-)
Slightly limited		28 (18.8)		27 (18.9)		1 (16.7)
Limited		67 (45.0)		66 (46.2)		1 (16.7)
Fairly limited		34 (22.8)		32 (22.4)		2 (33.3)
Very limited		19 (12.8)		17 (11.9)		2 (33.3)
Assuming a vaccine is discovered, would you be willing to be vaccinated against SARS-CoV-2? n (%)	149		143		6	
Yes		53 (35.6)		52 (36.4)		1 (16.7)
No		26 (17.4)		23 (16.1)		3 (50)
Don’t know		79 (47.0)		68 (47.6)		2 (33.3)
How important do you consider vaccinations in general? n (%)	145		140		5	
Not important at all		6 (4.1)		5 (3.6)		1 (20)
Slightly important		9 (6.2)		8 (5.7)		1 (20)
Important		36 (24.8)		33 (23.6)		3 (60)
Fairly important		39 (26.9)		39 (27.9)		0 (-)
Very important		55 (37.9)		55 (39.9)		0 (-)

© 2022 Forster J et al. *JAMA Network Open.*

eTable 3: Factors Associated With and Reasons for Declined Participation in Parents

eTable 3a. Factors associated with parents' willingness to participate in respiratory surveillance procedures of children in daycare centers for SARS-CoV-2 in univariable and in multivariable* logistic regression analyses. Only one parent per household filled in the questionnaire. OR: odds ratio, CI: confidence interval, 1.00: reference category.

Variable	Univariable OR (95%-CI)	p-value	Mulitvariable OR (95%-CI)	p-value
Proposed study module (defined by type of respiratory sampling)		<0.0001		<0.0001
1 (2x nasal swab/week)	0.14 (0.07-0.29)		0.11 (0.05-0.27)	
2 (1x nasal swab/week)	0.17 (0.08-0.38)		0.18 (0.07-0.46)	
3 (2x sputum/week)	1.00		1.00	
4 (pharyngeal swab in case of symptoms)	0.45 (0.23-0.86)		0.36 (0.16-0.77)	
Age of parent (years)		0.1175		0.6879
<30	0.46 (0.21 – 1.01)		0.64 (0.24-1.68)	
30-34	0.72 (0.41-1.26)		0.72 (0.37-1.41)	
35-39	1.04 (0.60 – 1.79)		0.90 (0.47-1.73)	
≥40	1.00		1.00	
Parent female vs. male	0.67 (0.41 -1.12)	0.1249	0.96 (0.52-1.77)	0.8978
School education		<0.0001		0.0100
None, Secondary Comprehensive, or Other	1.00		1.00	
Secondary	2.10 (0.93 -4.72)		1.9 (0.72-5.05)	
Grammar	6.19 (2.98-12.86)		3.46 (1.40 -8.56)	
SARS-COV-2 considered dangerous for parent/family (not/somewhat/dangerous vs fairly/very dangerous)	1.19 (0.77 – 1.83)	0.4320	1.49 (0.86-2.57)	0.1560
SARS-COV-2 considered dangerous for society (not/somewhat/dangerous vs fairly/very dangerous)	0.54 (0.35 – 0.83)	0.0051	0.75 (0.42-1.37)	0.3514
Personal knowledge of SARS-CoV-2 infection/disease/fatality (yes vs no)	1.62 (1.05 -2.50)	0.0283	1.01 (0.60-1.70)	0.9774
Restrictive measures against SARS-CoV-2 considered important (not/slightly/important vs fairly/very important)	0.37 (0.22 -0.61)	0.0001	0.85 (0.43-1.68)	0.6371
Extent of limitation felt in personal life by SARS-CoV-2 (not/slightly/limited vs. fairly/very limited)	2.48 (1.63 – 3.77)	<0.0001	1.78 (1.08-2.94)	0.0237
Willingness to be vaccinated against SARS-CoV-2		<0.0001		0.0001
Yes	8.80 (4.61-16.79)		5.01 (2.25-11.18)	
Don't know	3.05 (1.64-5.68)		2.19 (1.04-4.60)	
No	1.00		1.00	
Vaccinations considered important in general (not/slightly/important vs fairly/very important)	0.13 (0.07 -0.25)	<0.0001	0.31 (0.15-0.63)	0.0014

*Adjusted for study module, age, parent female vs. male, school education, SARS-COV-2 considered dangerous for parent/family, SARS-COV-2 considered dangerous for society, personal knowledge of SARS-CoV-2 infection/disease/fatality, restrictive measures against SARS-CoV-2 considered important, extent of limitation felt in personal life by SARS-CoV-2, willingness to be vaccinated against SARS-CoV-2, vaccinations considered important in general

eTable 3b. Main reasons provided by 171 parents of children in daycare centers for not participating in the respiratory surveillance for SARS-CoV-2

Reason given for refusing participation in the respiratory surveillance	N (%)
Considered participation in study/respiratory surveillance a potentially negative experience for the child	27 (15.8)
Previous negative experience (e.g. child refusal) with respiratory sampling	10 (5.8)
Objected to participate in any studies on / with children	9 (5.3)
Personal situation / circumstances	9 (5.3)
No interest in study participation	6 (3.5)
Objected respiratory testing of asymptomatic children / believed testing is unnecessary	5 (2.9)
Stated that child is / was already tested for SARS-CoV-2	5 (2.9)
Denial / sceptical attitude towards SARS-CoV-2 pandemic	5 (2.9)
Concerns regarding data protection / amount of data collected	4 (2.3)
Considered study information given as insufficient	3 (1.8)
Language barrier	2 (1.2)
Several reasons given	7 (4.1)
No explicit reason given	79 (46.2)
All	171 (100.0)

© 2022 Forster J et al. *JAMA Network Open.*

eTable 4: Dropout Rates in Modules 1-3 and Unsuccessful Testing in Module 4

eTable 4a Drop-out rates of participants (children and CCW in day care centers) from SARS-CoV-2 surveillance by biweekly (module 1) and weekly (module 2) nasal swabs and by saliva sampling (module 3)

	**Module 1**		**Module 2**		**Module 3**	
Drop out n(%) of all surveillance participants (N)	N	n (%)	N	n (%)	N	n (%)
Children	44	11 (25.0)	43	10 (23.3)	120	3 (2.5)
CCW*	18	0 (0.0)	12	1 (8.3)	36	3 (8.3)
**Overall**	**62**	**11 (17.7)**	**55**	**11 (20.0)**	**156**	**6 (3.9)**

One child and 4 CCW who were initially considered eligible and had given informed consent to respiratory sampling were excluded from drop-out analysis, as they could not participate in respiratory sampling for reasons unrelated to the respiratory surveillance measures.

eTable 4b. Rates of unsuccessful testing of participants in SARS-CoV-2 surveillance by respiratory sampling upon symptoms (module 4) over the regular study period of 12 weeks. Successful testing of participants was defined as follows: children and CCW in day care centers and their household members, contacting the study center in case of symptoms, with respiratory sampling result (PCR from oropharyngeal swab) available within 72h after symptom start/call to the study center. Overall, 220 tests from 179 symptomatic participants were scheduled; 9 tests were excluded from analysis due to missing date of test result.

	Module 4<br>Rate of unsuccessful testing	
	n/ N	% (95%-CI)*
Total	12 / 211	5.7 (3.3-9.7)
Testing of participating children plus household members	12[#] / 161[##]	7.5 (4.3-12.6)
Testing of CCW plus household members	0 / 50[###]	0

*95%-CI after Score Wilson; [#]7 tests of children and 5 of household members of children; [##] 92 tests of children, 69 of household members of children; [###] 35 tests of CCW, 15 of household members of CCW

© 2022 Forster J et al. *JAMA Network Open.*

eTable 5. Number of Samples Tested for SARS-CoV-2 in DCC With Continuous Surveillance (Modules 1-3)

**Participants**	**Module 1**		**Module 2**		**Module 3**		**Any sample**	
	No. of tests	No. of positive tests	No. of tests	No. of positive tests	No. of tests	No. of positive tests	No. of tests	No. of positive tests
Children (study period)	712	0	343	0	2387	0	3442	0
Children (lockdown)	123	0	35	0	594	1	752	1
**Children (total)**	**835**	**0**	**378**	**0**	**2981**	**1**	**4194**	**1**
CCW (study period)	347	0	122	0	630	0	1099	0
CCW (lockdown)	118	0	47	0	311	0	476	0
**CCW (total)**	**465**	**0**	**169**	**0**	**941**	**0**	**1575**	**0**
Total No of samples (children + CCW; study period + lockdown)	**1300**	**0**	**547**	**0**	**3922**	**1**	**5769**	**1**

DCC: Day care center(s); CCW: child care worker(s);
No= number

© 2022 Forster J et al. *JAMA Network Open.*

eTable 6. Model Parameters Used for All Simulations

Parameter	Parameter		Values
General model parameters	Number of simulations		40 000
	Simulation time t_{max}		30 days
	Time step Δt		1 day
DCC parameters	Number of groups		4
	Number of children/group		17 – 26
	Number of CCW/group		2 – 4
	Number of heads		2
Infection related parameters	Ratio asymptomatic/symptomatic		20%
	Asymptomatic spreading		42% [11]
	Development of immunity after infection		100%
	Loss of immunity after infection		0%
Viral load parameters [9]	Time between viral peak and symptom onset		~U(1,3)
	Time point viral peak		~N(4.3,0.92)
	Viral peak children		~N(7.44,0.7)
	Viral peak adults		~N(7.94,0.7)
	Slope viral peak until $<10^6$		~N(0.17,0.02)
	Days infectious after viral peak		~U(7,9) [12]
Aerosol transmission and infection risk calculator in indoor environments [10]	Room ventilation		0.35 (passive)
	Total mask efficiency		0.0 (no mask)
	Average class room volume		60 m2 ·3 m = 180 m3
	Shared room volume		60 m2 ·3 m = 180 m3
	CCW common room volume		20 m2 ·3 m = 60 m3
	Head office volume		10 m2 ·3 m = 30 m3
	Infectious episode	children – children	6h
		children – CCW	6h
		CCW – CCW (intra group)	6h
		CCW – CCW (inter group)	1h
		head – CCW (inter group)	0.5h
PCR Test quality criteria	Sensitivity		99.99%
	Limit of detection		10^3 copies/ml
	Specificity		100%
	Result availability time		0.5 d

© 2022 Forster J et al. *JAMA Network Open.*

eTable 7. Varied Parameters and Processes in the Model

<table>
<tr><th>Parameter</th><th colspan="2">Values</th></tr>
<tr><td>Children participation rate</td><td colspan="2">$(0, 0.1, 0.2, \dots, 1.0)$</td></tr>
<tr><td rowspan="6">Test frequency and test days</td><td>Test frequency [1/week]</td><td>Test days</td></tr>
<tr><td>No Testing</td><td>NA</td></tr>
<tr><td>1</td><td>Mo, We, Fr</td></tr>
<tr><td>2</td><td>Mo – We, Mo – Th, Tu – Th, Wed – Fr</td></tr>
<tr><td>3</td><td>Mo – Tu – Fr</td></tr>
<tr><td>5</td><td>Mo – Tu – We – Th – Fr</td></tr>
<tr><td>Starting condition</td><td colspan="2">1. One infected child that is asymptomatic and does not participate in regular testing
2. One infected child that is randomly symptomatic or asymptomatic and randomly does participate in regular testing
3. One infected CCW that is randomly symptomatic or asymptomatic and is participating in regular testing</td></tr>
<tr><td>Quarantine policy</td><td colspan="2">1. Positive tested individual is isolated; all group members of the corresponding children and CCW are quarantined
2. Positive tested individual is isolated; the group stays in the DCC</td></tr>
</table>

© 2022 Forster J et al. *JAMA Network Open.*

eFigure 1. Flowcharts of Wue-KiTa-CoV Study

eFigure 1a

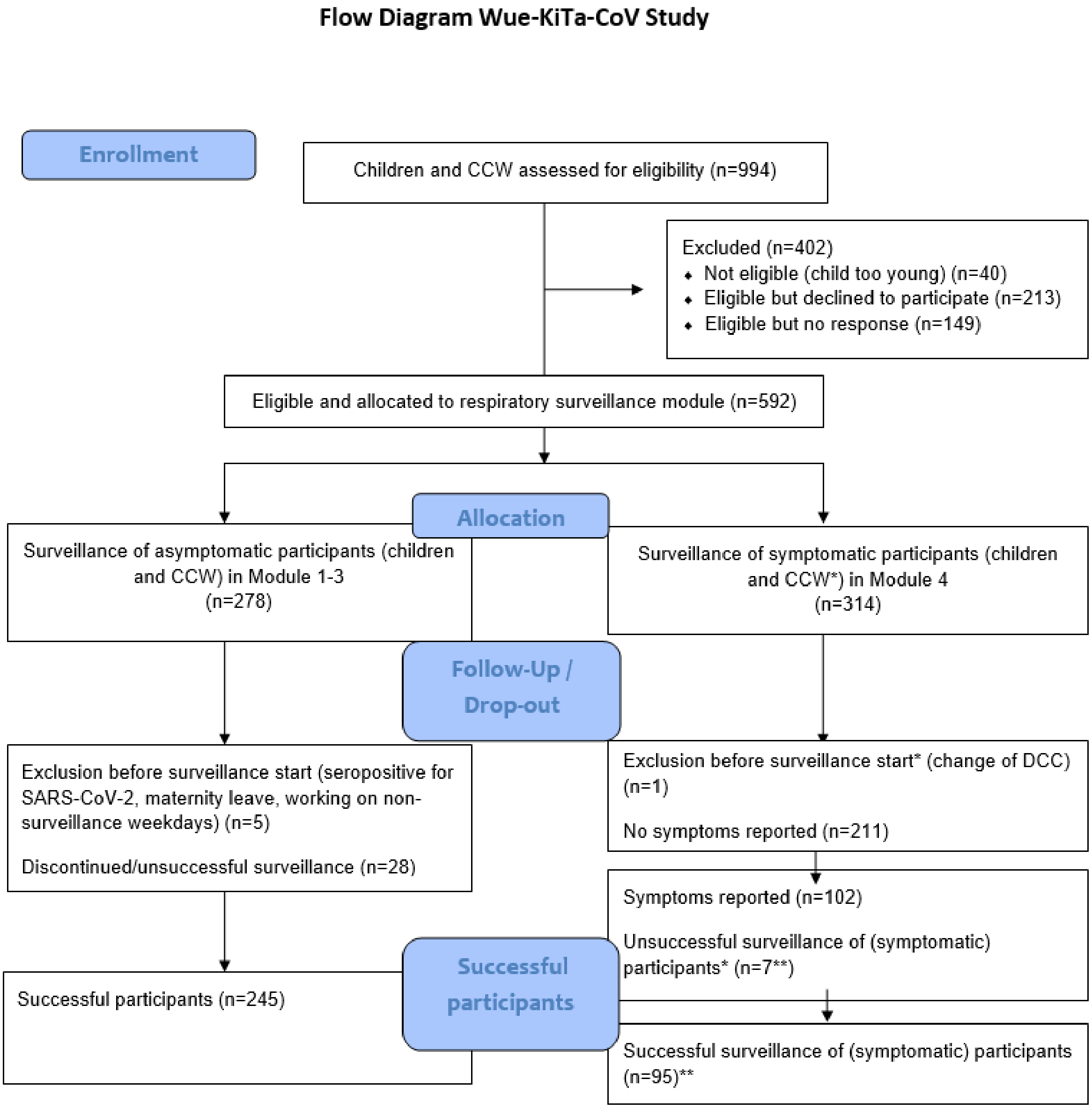

eFigure 1a. Flow chart Wue-KiTa-CoV study – Overview participating children and CCW in modules 1-3 and module 4. All data in the Flow chart refer to the regular 12-weeks surveillance period. *Regarding surveillance of symptomatic participants in Module 4, the Flow chart refers only to recruited children and CCW, not to the symptomatic household members of children/CCW. There were additional 77 symptomatic household members and for 5 household members surveillance was not successful. For details on Module 4 participants, see eFigure 1b.

© 2022 Forster J et al. *JAMA Network Open.*

eFigure 1b

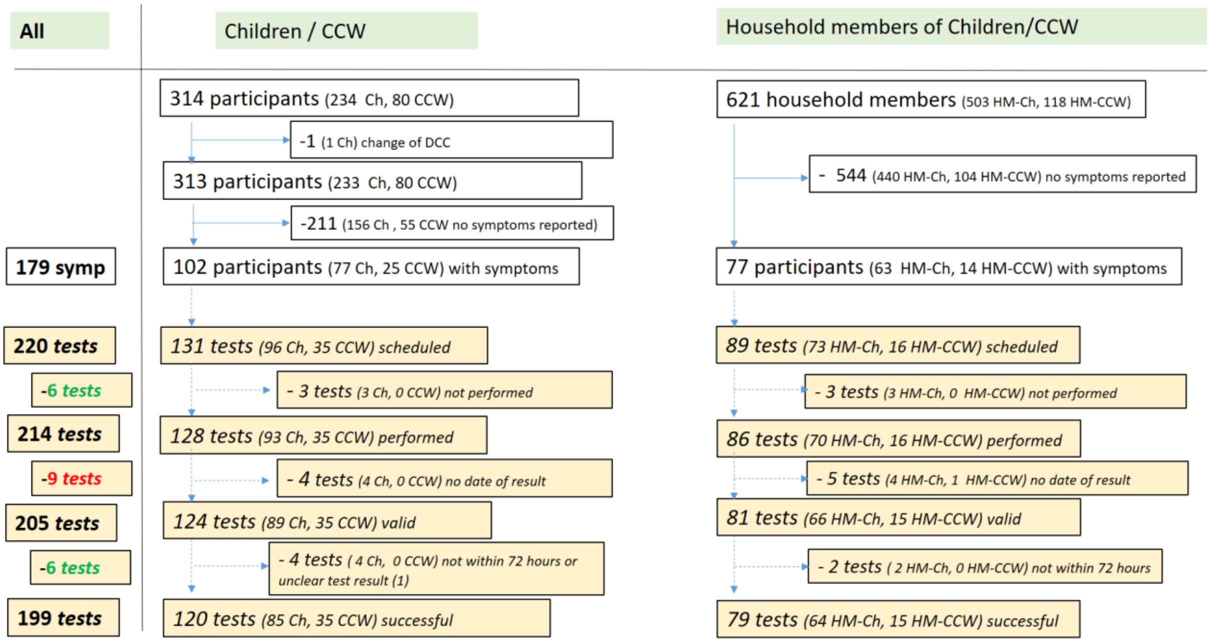

eFigure 1b. Flow chart Wue-KiTa-CoV Study for Module 4 – participants and testings in children, CCW and their household members. All data refer to the regular 12 weeks study observation period. Regarding household members, detailed information on household size were missing from 4 households of children/parents (counted as 1 household member each) and 5 CCW (counted as 0 household members). From 220 tests performed on 179 symptomatic participants, the 9 tests with no date of test result were excluded as 'missing' from primary endpoint analysis. The 6 tests 'not performed' and the 6 tests with the result or not available within 72hours or unclear result were counted as "unsuccessful" regarding primary endpoint analysis. Ch = child/children, CCW=childcare worker, HM-Ch: household member of recruited child, HM-CCW: household member of recruited CCW.

© 2022 Forster J et al. *JAMA Network Open.*

eFigure 2: General and Specific Measures Implemented Against SARS-CoV-2

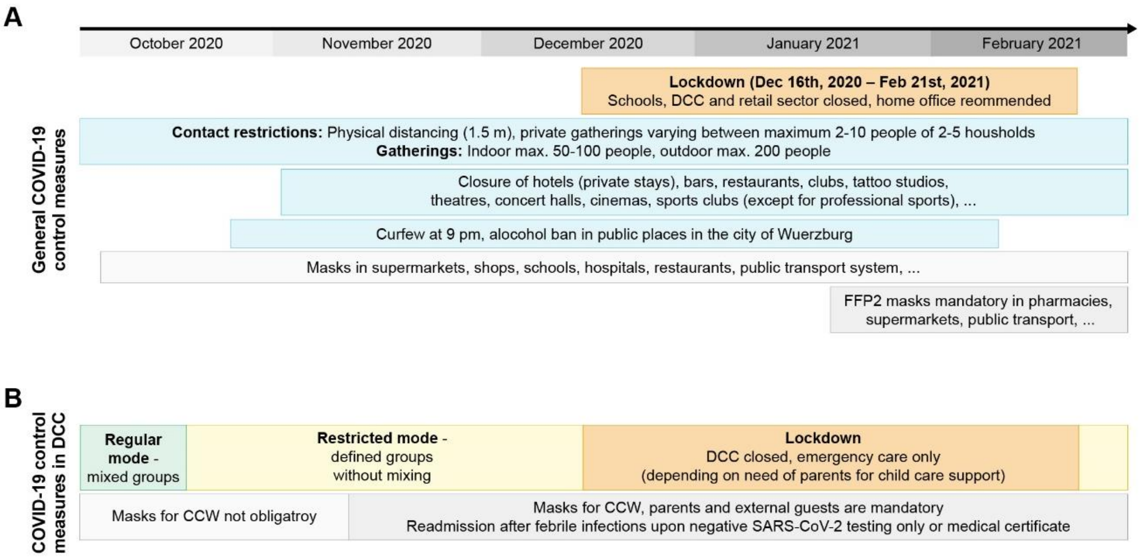

eFigure 2: **A)** General measures implemented against SARS-CoV-2 spread by the federal and local government; **B)** Specific measures for DCC in Wuerzburg

© 2022 Forster J et al. *JAMA Network Open.*

eFigure 3. Final Assessment of Study Measures by Parents and CCWs

Supplement eFigure 3A. Final assessment of parents from daycare centers regarding the study measures judged at the end of the respiratory surveillance ('week12') for SARS-CoV-2, overall and stratified by module 1-3 vs module 4 (WueKiTaCoV Study, Germany, 2020-2021)

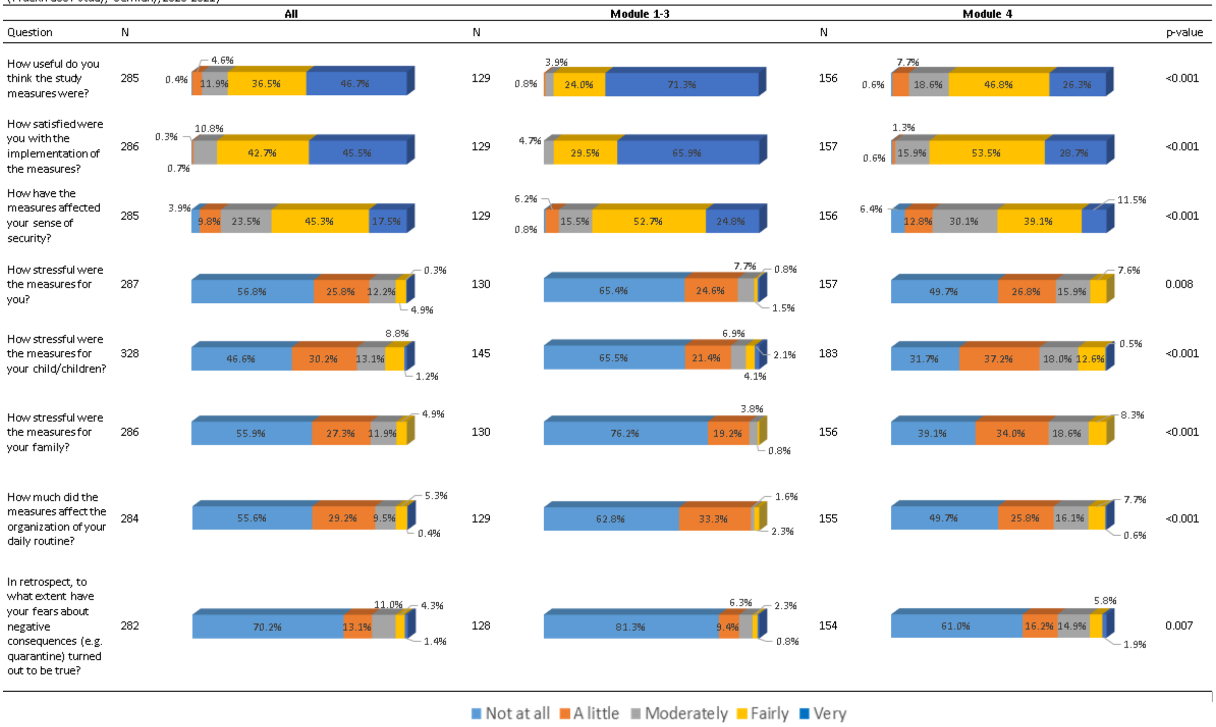

Supplemental Figure eFig 3B. Final assessment of CCW from daycare centers regarding the study measures judged at the end of the respiratory surveillance ('week12') for SARS-CoV-2, overall and stratified by module 1-3 vs module 4 (WueKiTaCoV Study, Germany, 2020-2021)

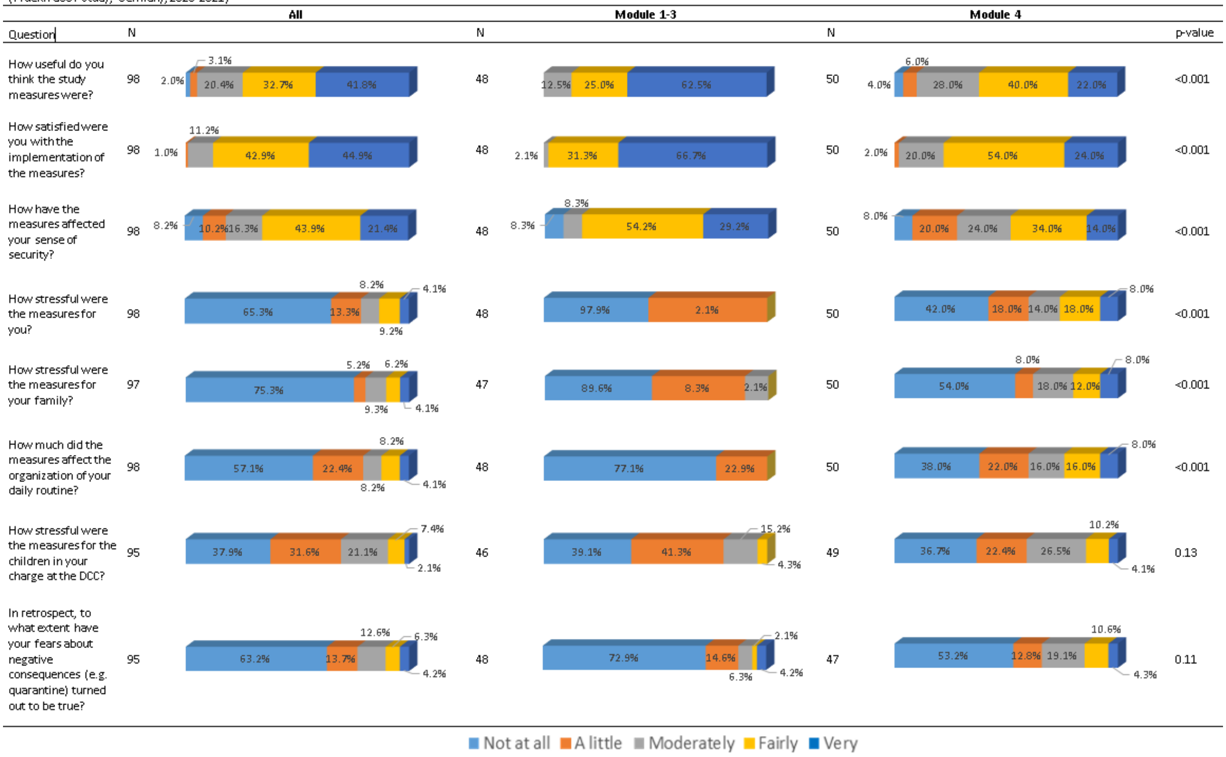

eFigure 3 – Final assessment of study measures by **(A)** parents and **(B)** CCW, judged at the end of the respiratory surveillance period, overall and stratified by module 1-3 vs module 4**.**

© 2022 Forster J et al. *JAMA Network Open.*

eFigure 4. Events Associated With Potential Introduction of SARS-CoV-2 Into a DCC.

Event Number	1	2	3	4	5	6	7	8	9	10	11	12
Module	4	3	4	4	4	3	4	3	1	4	2	4
Indication for Testing<br>A= asymptomatic testing (Modules 1-3)<br>S= symptomatic testing<br>C= contact tracing	S	C	C	S	C	A	S	C	C	C	C	unknown

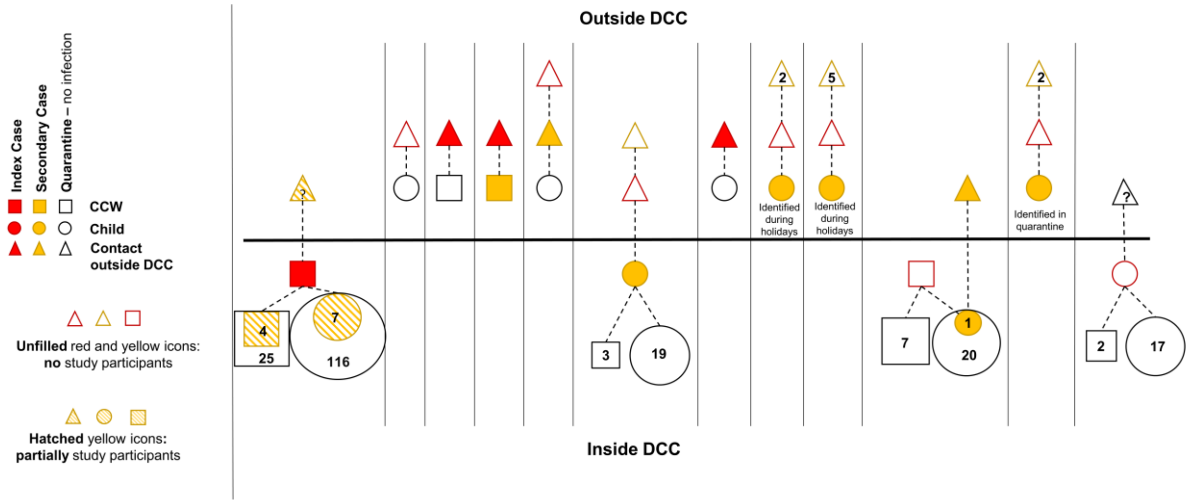

eFigure 4 – Events associated with potential introduction of SARS-CoV-2 into a DCC. Module indicates the participant’s designated module per study protocol. Asymptomatic (A), symptomatic (S) and contact tracing (C) characterizes the individual indication for testing, regardless of the designated module.

© 2022 Forster J et al. *JAMA Network Open.*

eFigure 5. Modeling Results Scenario 1

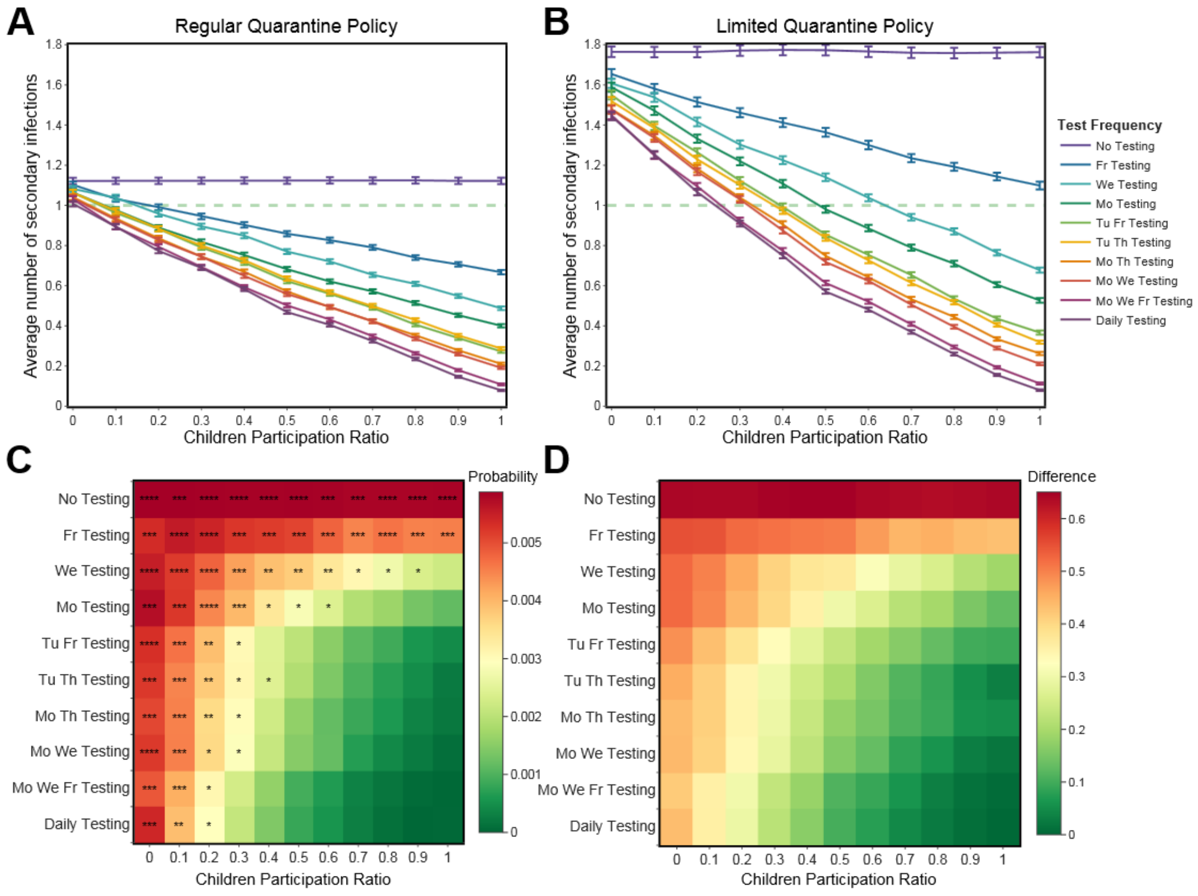

eFigure 5 – Simulation results of the virtual infection spread model for scenario 1. Waiting time until the test result is available is 0.5 days. Average number of secondary infections is shown for various children participation rates and test frequencies for regular **(A)** and limited **(B)** quarantine policies. Error bars indicate the standard deviation of the estimation of the ASI. **(C)** Comparison of the quarantine policies, where colours represent the probability to get more secondary infections and stars indicate, whether results for different quarantine policies are significantly different with significance levels (**** $p < 0.001$, *** $p < 0.005$, ** $p < 0.01$, * $p < 0.05$). **(D)** Difference between mean values of all scenarios for the different quarantine policies. For the test participation of children of 50% and “Mo testing”, “Mo-We testing” and “Mo-We-Fr testing” the ASI will reduce by 44.7 %, 59.48% and 65.38% compared to “No testing” for the limited quarantine policy, respectively.

© 2022 Forster J et al. *JAMA Network Open.*

eFigure 6. Modeling results scenario 2

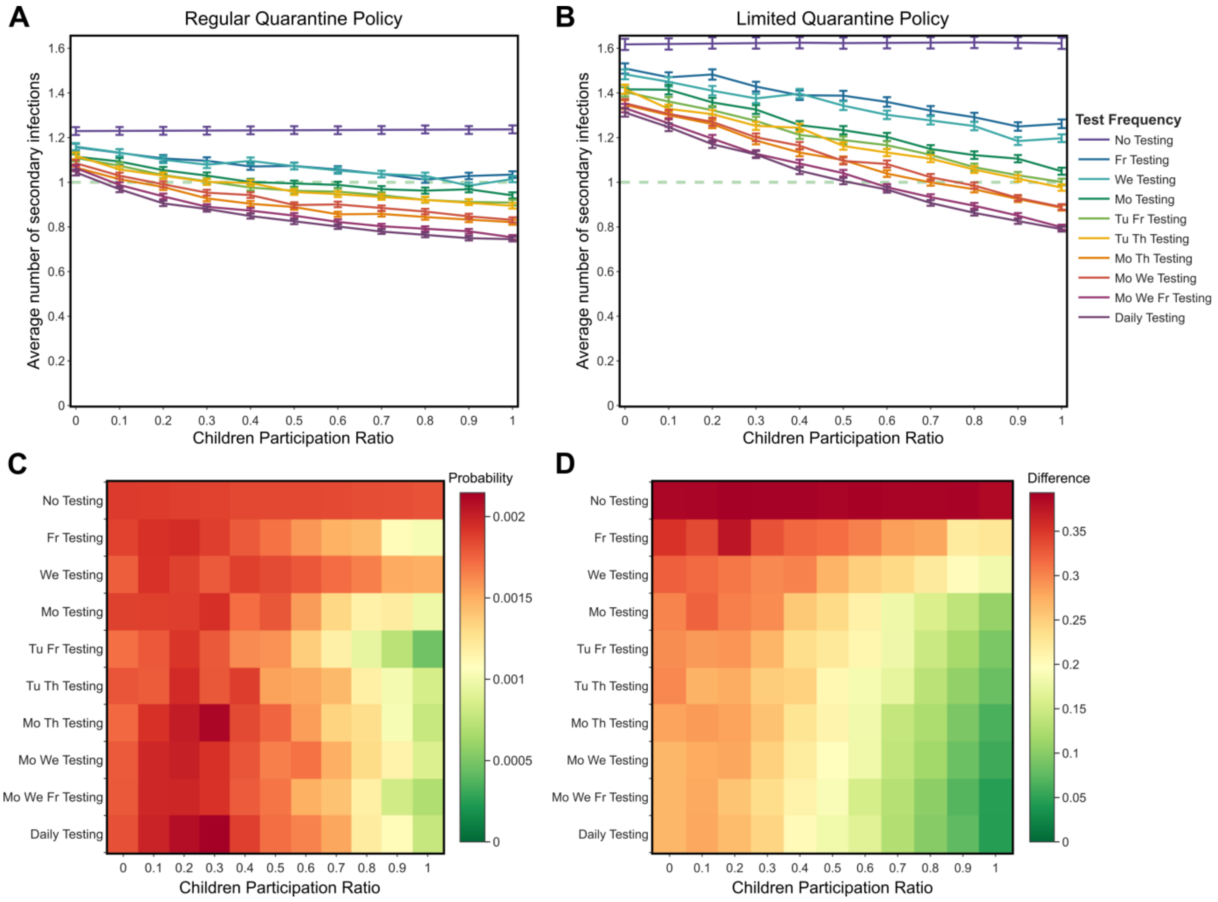

eFigure 6. Simulation Results of the Virtual Infection Spread Model for Scenario 2.

Waiting time until the test result is available is 0.5 days. Average number of secondary infections is shown for various children participation rates and test frequencies for regular **(A)** and limited **(B)** quarantine policies. Error bars indicate the standard deviation of the estimation of the ASI. **(C)** Comparison of the quarantine policies, where colours represent the probability to get more secondary infections and stars indicate, whether results for different quarantine policies are significantly different with significance levels (**** p < 0.001, *** p < 0.005, ** p < 0.01, * p < 0.05). **(D)** Difference between mean values of all scenarios for the different quarantine policies. For all test frequencies >0 and all participation rates >0 the ASI is higher than for scenario 1 as infection can only be detected if at least one secondary infection occurs that either shows symptoms or participates in testing. For the test participation of children of 50% and "Mo testing", "Mo-We testing" and "Mo-We-Fr testing" the ASI will reduce by 19.48%, 27.19% and 31.05% compared to "No testing" for the regular quarantine policy, respectively. Similarly, for the limited quarantine policy the ASI will reduce by 24.03 %, 32.51% and 35.9% respectively.

© 2022 Forster J et al. *JAMA Network Open.*

eFigure 7. Modeling Results Scenario 3

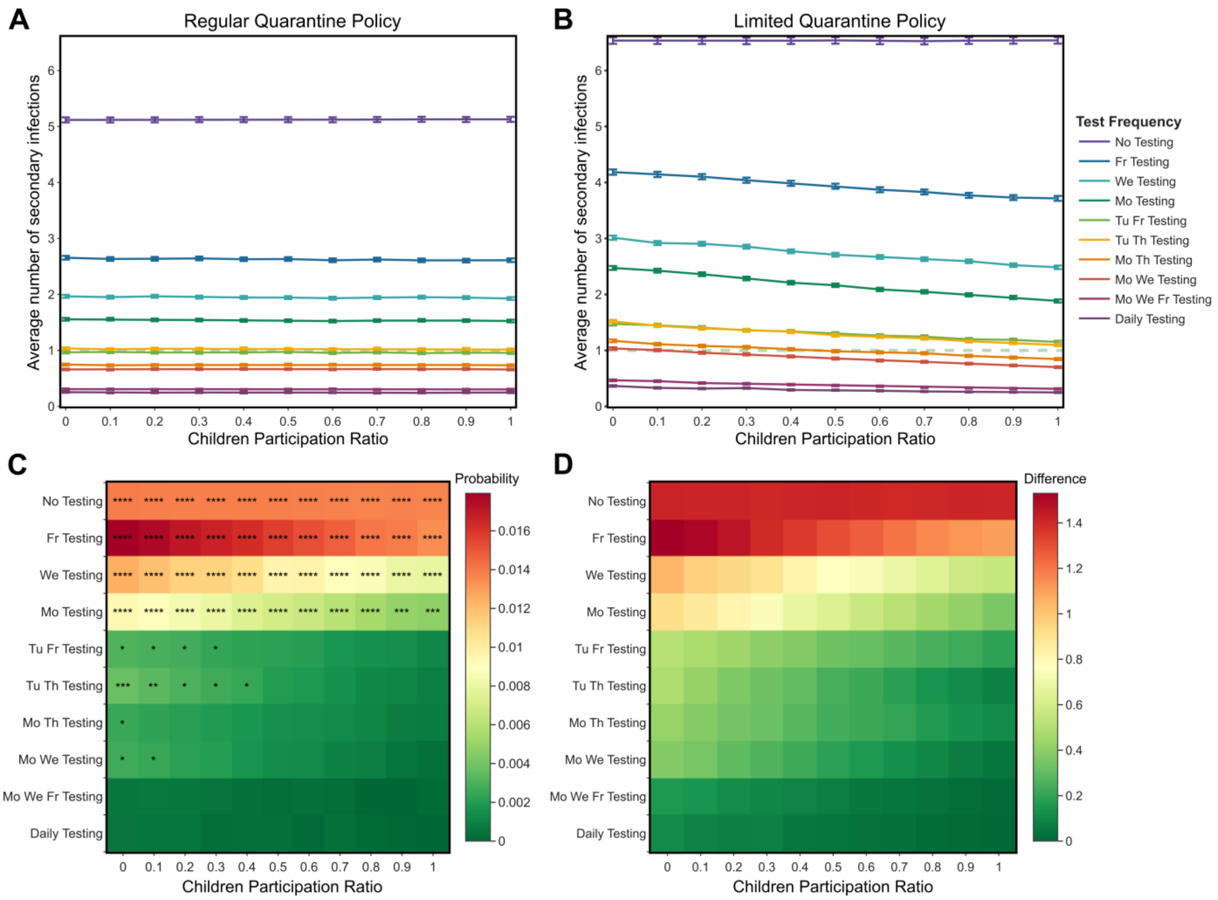

eFigure 7 – Simulation results of the virtual infection spread model for the scenario 3. Waiting time until the test result is available is 0.5 days. Average number of secondary infections is shown for various children participation rates and test frequencies for regular **(A)** and limited **(B)** quarantine policies. Error bars indicate the standard deviation of the estimation of the ASI. **(C)** Comparison of the quarantine policies, where colours represent the probability to get more secondary infections and stars indicate, whether results for different quarantine policies are significantly different with significance levels (**** $p < 0.001$, *** $p < 0.005$, ** $p < 0.01$, * $p < 0.05$). **(D)** Difference between mean values of all scenarios for the different quarantine policies. For the test participation of children of 50% and “Mo testing”, “Mo-We testing” and “Mo-We-Fr testing” the ASI will reduce by 66.9%, 86.9% and 94.3% compared to “No testing” for the limited quarantine policy, respectively.

© 2022 Forster J et al. *JAMA Network Open.*

eFigure 8. Modeling: Effects of Longer Time to Result on Scenario 1

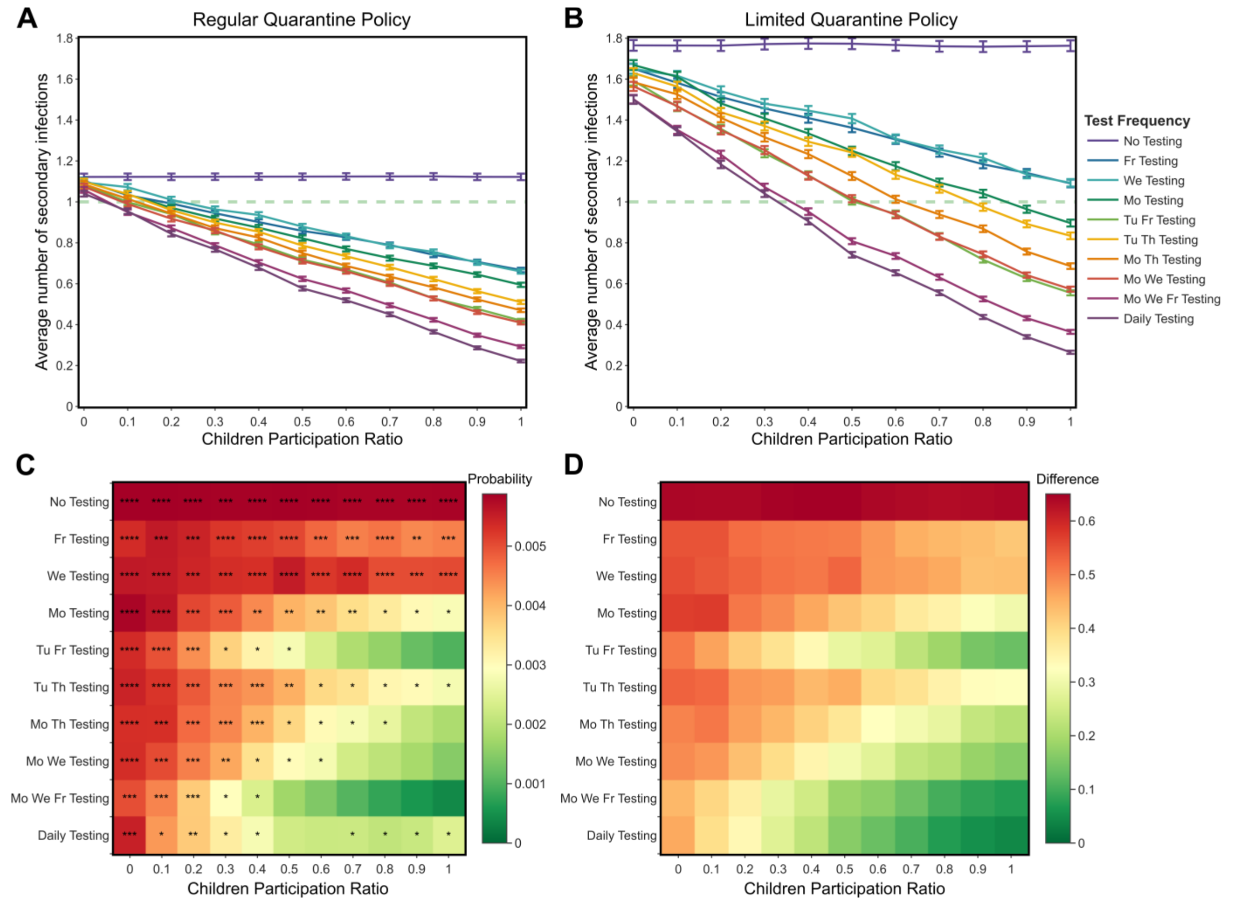

eFigure 8 – Simulation results of the virtual infection spread model for the scenario 1. Waiting time until the test result is available is 1.5 days. Average number of secondary infections is shown for various children participation rates and test frequencies for regular **(A)** and limited **(B)** quarantine policies. Error bars indicate the standard deviation of the estimation of the ASI. **(C)** Comparison of the quarantine policies, where colours represent the probability to get more secondary infections and stars indicate, whether results for different quarantine policies are significantly different with significance levels (**** $p < 0.001$, *** $p < 0.005$, ** $p < 0.01$, * $p < 0.05$). **(D)** Difference between mean values of all scenarios for the different quarantine policies.

© 2022 Forster J et al. *JAMA Network Open.*

eFigure 9. Modeling: Effects of Longer Time to Result on Scenario 2

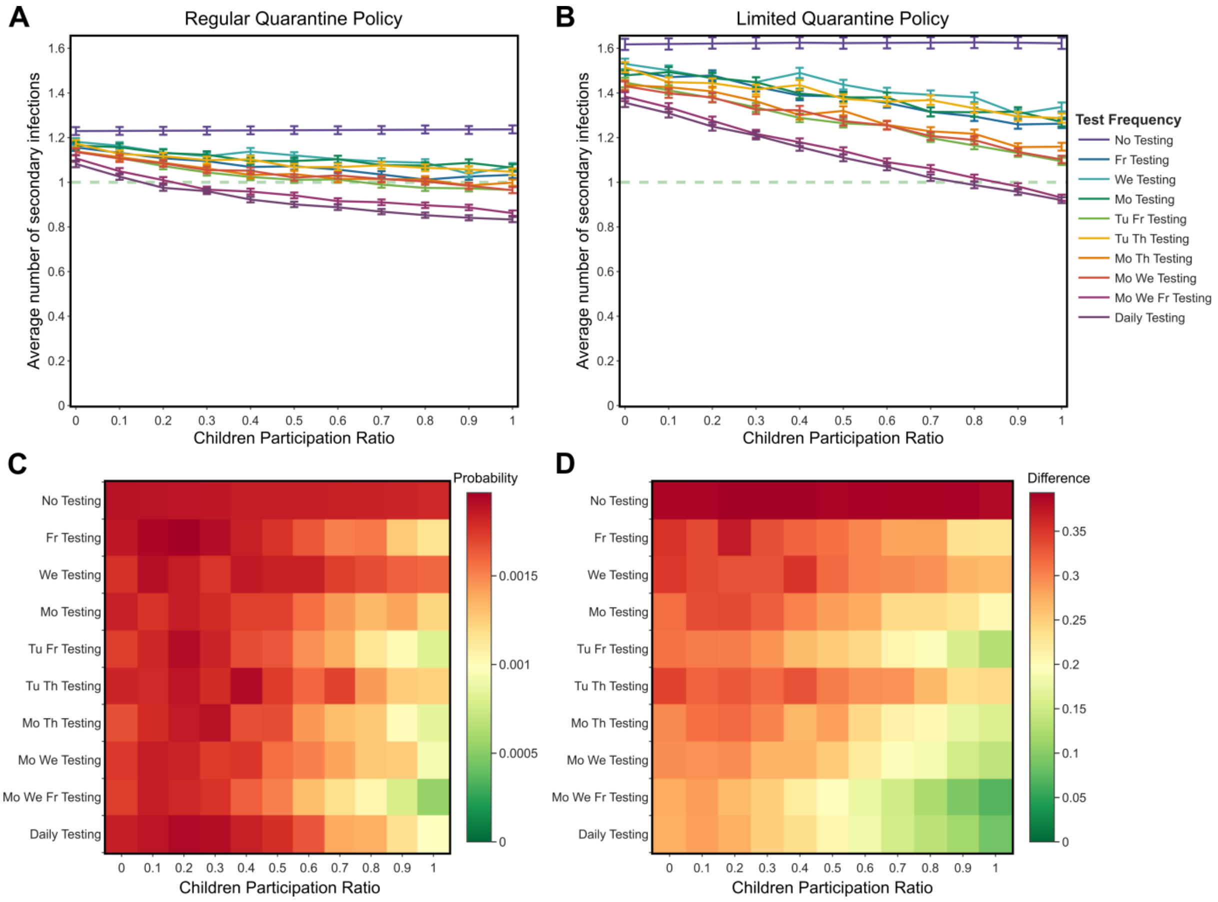

eFigure 9 – Simulation results of the virtual infection spread model for the scenario 2. Waiting time until the test result is available is 1.5 days. Average number of secondary infections is shown for various children participation rates and test frequencies for regular **(A)** and limited **(B)** quarantine policies. Error bars indicate the standard deviation of the estimation of the ASI. **(C)** Comparison of the quarantine policies, where colours represent the probability to get more secondary infections and stars indicate, whether results for different quarantine policies are significantly different with significance levels (**** $p < 0.001$, *** $p < 0.005$, ** $p < 0.01$, * $p < 0.05$). **(D)** Difference between mean values of all scenarios for the different quarantine policies.

© 2022 Forster J et al. *JAMA Network Open.*

eFigure 10. Modeling: Effects of Longer Time to Result on Scenario 3

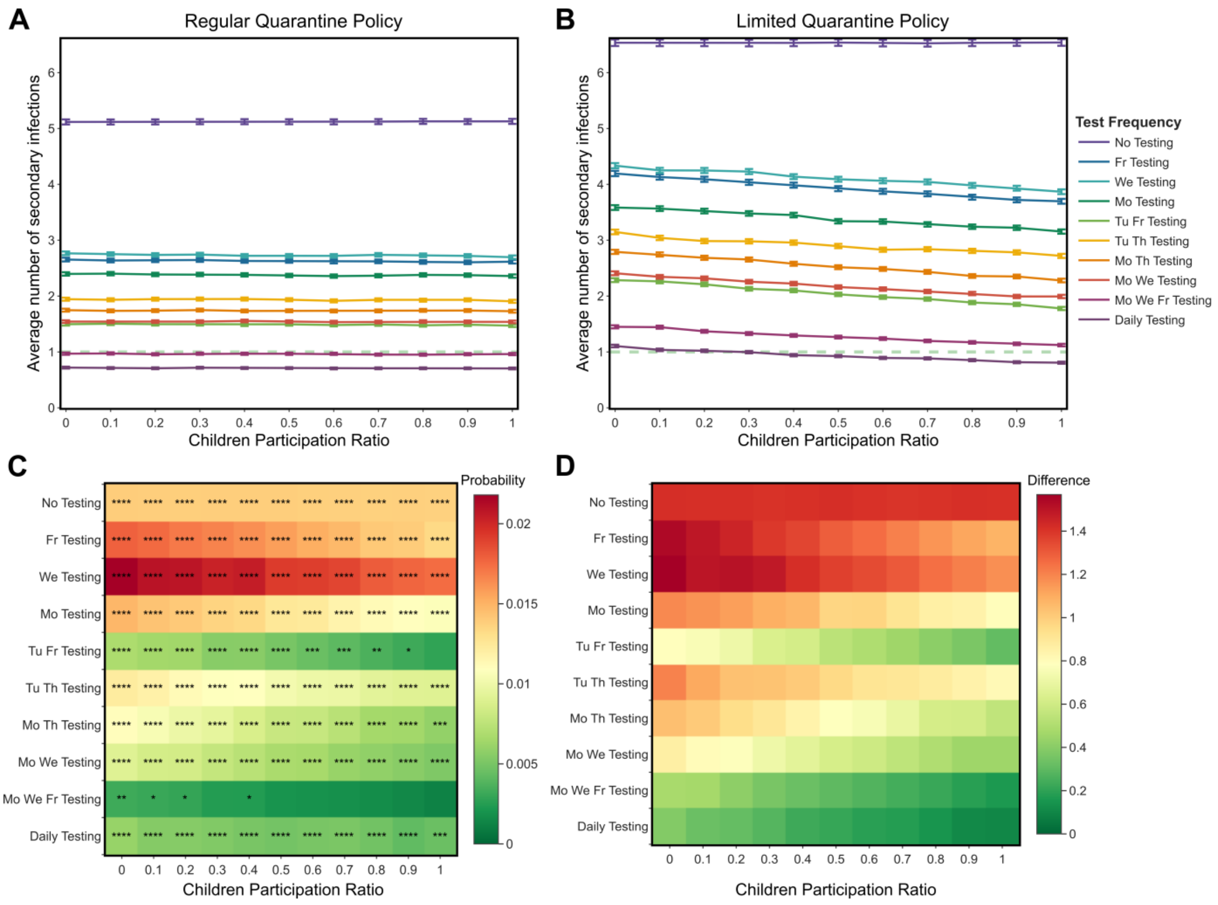

eFigure 10. Simulation results of the virtual infection spread model for the scenario 3. Waiting time until the test result is available is 1.5 days. Average number of secondary infections is shown for various children participation rates and test frequencies for regular **(A)** and limited **(B)** quarantine policies. Error bars indicate the standard deviation of the estimation of the ASI. **(C)** Comparison of the quarantine policies, where colours represent the probability to get more secondary infections and stars indicate, whether results for different quarantine policies are significantly different with significance levels (**** $p < 0.001$, *** $p < 0.005$, ** $p < 0.01$, * $p < 0.05$). **(D)** Difference between mean values of all scenarios for the different quarantine policies.

© 2022 Forster J et al. *JAMA Network Open.*

eReferences

1. Statistisches Bundesamt. Familien und Familienmitglieder nach Bundesländern. 2020; https://www.destatis.de/DE/Themen/Gesellschaft-Umwelt/Bevoelkerung/Haushalte-Familien/Tabellen/2-3-familien-bundeslaender.html Accessed 7/01/2020.
2. Krone M, Gütling J, Wagener J, et al. Performance of three SARS-CoV-2 immunoassays, three rapid lateral flow tests and a novel bead-based affinity surrogate test for the detection of SARS-CoV-2 antibodies in human serum. *Journal of clinical microbiology.* 2021:JCM.00319-00321.
3. Bethell CD, Read D, Stein RE, Blumberg SJ, Wells N, Newacheck PW. Identifying children with special health care needs: development and evaluation of a short screening instrument. *Ambulatory pediatrics : the official journal of the Ambulatory Pediatric Association.* 2002;2(1):38-48.
4. Cox B, Van Oyen H, Cambois E, et al. The reliability of the minimum European health module. *International journal of public health.* 2009;54(2):55-60.
5. Achenbach TM. *The Achenbach system of empirically based assessment (ASEBA): Development, findings, theory, and applications.* University of Vermont, Research Center for Children, Youth, & Families; 2009.
6. Kroenke K, Spitzer RL, Williams JB, Löwe B. An ultra-brief screening scale for anxiety and depression: the PHQ–4. *Psychosomatics.* 2009;50(6):613-621.
7. Löwe B, Wahl I, Rose M, et al. A 4-item measure of depression and anxiety: validation and standardization of the Patient Health Questionnaire-4 (PHQ-4) in the general population. *Journal of affective disorders.* 2010;122(1-2):86-95.
8. Köpke K, Prahm K, Buda S, Haas W. Evaluation einer ICD-10-basierten elektronischen Surveillance akuter respiratorischer Erkrankungen (SEEDARE) in Deutschland. *Bundesgesundheitsblatt - Gesundheitsforschung - Gesundheitsschutz.* 2016;59(11):1484-1491.
9. Jones TC, Biele G, Mühlemann B, et al. Estimating infectiousness throughout SARS-CoV-2 infection course. *Science.* 2021:eabi5273.
10. Lelieveld J, Helleis F, Borrmann S, et al. Model Calculations of Aerosol Transmission and Infection Risk of COVID-19 in Indoor Environments. *International journal of environmental research and public health.* 2020;17(21).
11. Byambasuren O, Cardona M, Bell K, Clark J, McLaws M-L, Glasziou P. Estimating the extent of asymptomatic COVID-19 and its potential for community transmission: systematic review and meta-analysis. *medRxiv* Published 09/13/2020. Accessed 06/06/2021.; https://www.medrxiv.org/content/medrxiv/early/2020/09/13/2020.05.10.20097543.full.pdf.
12. Larremore DB, Wilder B, Lester E, et al. Test sensitivity is secondary to frequency and turnaround time for COVID-19 screening. *Science Advances.* 2021;7(1):eabd5393.

© 2022 Forster J et al. *JAMA Network Open.*
