# Supplementary material for: Feasibility of SARS-CoV-2 Surveillance Testing Among Children and Childcare Workers at German Day Care Centers: A Nonrandomized Controlled Trial
Source: JAMA Netw Open. 2022 Jan 4;5(1):e2142057. doi: 10.1001/jamanetworkopen.2021.42057 (PMC8728621; doi:10.1001/jamanetworkopen.2021.42057)
Supplement: Supplement 3. — Wü-KiTa-CoV-Study Group Members [file jamanetwopen-e2142057-s003.pdf]

Supplemental Online Content: Nonauthor Collaborators

*Indicates required information. Only first name, last name, and suffix will appear in PubMed.

*Group Name(s): The Wü-KiTa-CoV Study Group							
***First Name and Middle Initial(s)**	***Last Name**	***Suffix (eg, Jr, III)**	Academic Degrees	Institution	Location (city, state/province, country)	Role or Contribution, eg, chair, principal investigator	Group (if more than 1 Group listed in the byline) and/or Subgroup (eg, Steering Committee)
Anastasia	Besenfelder			Institute for Hygiene and Microbiology, University of Wuerzburg	Würzburg, Germany	Technical support	
Lena	Buechner			Institute for Hygiene and Microbiology, University of Wuerzburg	Würzburg, Germany	Technical support	
Kathrin	Frei			Institute for Hygiene and Microbiology, University of Wuerzburg	Würzburg, Germany	Technical support	
Theresa	Höferth			Institute for Hygiene and Microbiology, University of Wuerzburg	Würzburg, Germany	Technical support	
Thiên-Trí	Lam		MD	Institute for Hygiene and Microbiology, University of Wuerzburg	Würzburg, Germany	Advice on coordination	
Sabrina	Mündlein			Institute for Hygiene and Microbiology, University of Wuerzburg	Würzburg, Germany	Technical support	
Christoph	Schoen		MD, PhD, MSc	Institute for Hygiene and Microbiology, University of Wuerzburg	Würzburg, Germany	Advice on coordination	
Alexandra	Schubert-Unkmeir		MD	Institute for Hygiene and Microbiology, University of Wuerzburg	Würzburg, Germany	Advice on coordination	
Lars	Wallstabe		PhD	Institute for Hygiene and Microbiology, University of Wuerzburg	Würzburg, Germany	communication with study participants	
Kerstin	Knies		PhD	Institute for Virology, University of Wuerzburg	Würzburg, Germany	Technical support	

Supplemental Online Content: Nonauthor Collaborators

*Indicates required information. Only first name, last name, and suffix will appear in PubMed.

*First Name and Middle Initial(s)	*Last Name	*Suffix (eg, Jr, III)	Academic Degrees	Institution	Location (city, state/province, country)	Role or Contribution, eg, chair, principal investigator	Group (if more than 1 Group listed in the byline) and/or Subgroup (eg, Steering Committee)
Rebecca	Richter			Institute for Virology, University of Wuerzburg	Würzburg, Germany	Technical support	
Abdullah	Almamy		MD	Department of Pediatrics, University Hospital Wuerzburg	Würzburg, Germany	Field activities	
Maxi	Bettkober		MD	Department of Pediatrics, University Hospital Wuerzburg	Würzburg, Germany	Field activities	
Viktoria	Engert		MD	Department of Pediatrics, University Hospital Wuerzburg	Würzburg, Germany	Field activities	
Phillipp	Fecher		MD	Department of Pediatrics, University Hospital Wuerzburg	Würzburg, Germany	Field activities	
Jonas	Fischer		MD	Department of Pediatrics, University Hospital Wuerzburg	Würzburg, Germany	Field activities	
Robin	Khan		MD	Department of Pediatrics, University Hospital Wuerzburg	Würzburg, Germany	Field activities	
Fabian	Kleindiek		MD	Department of Pediatrics, University Hospital Wuerzburg	Würzburg, Germany	Field activities	
Mirja	Müller		MD	Department of Pediatrics, University Hospital Wuerzburg	Würzburg, Germany	Field activities	
Lara	Müller-Scholden		MD	Department of Pediatrics, University Hospital Wuerzburg	Würzburg, Germany	Field activities	
Pauline	Nehm			Department of Pediatrics, University Hospital Wuerzburg	Würzburg, Germany	Field activities	
Jacobe	Rapp		MD	Department of Pediatrics, University Hospital Wuerzburg	Würzburg, Germany	Field activities	
Christina	Pfann		MD	Department of Pediatrics, University Hospital Wuerzburg	Würzburg, Germany	Field activities	
Maria	Riedmeier		MD	Department of Pediatrics, University Hospital Wuerzburg	Würzburg, Germany	Field activities	
George	Robinson		MD	Department of Pediatrics, University Hospital Wuerzburg	Würzburg, Germany	Field activities	

Supplemental Online Content: Nonauthor Collaborators

*Indicates required information. Only first name, last name, and suffix will appear in PubMed.

***First Name and Middle Initial(s)**	***Last Name**	***Suffix (eg, Jr, III)**	Academic Degrees	Institution	Location (city, state/province, country)	Role or Contribution, eg, chair, principal investigator	Group (if more than 1 Group listed in the byline) and/or Subgroup (eg, Steering Committee)
Sarah	Schnee		MD	Department of Pediatrics, University Hospital Wuerzburg	Würzburg, Germany	Field activities	
Anna-Lena	Sieg		MD	Department of Pediatrics, University Hospital Wuerzburg	Würzburg, Germany	Field activities	
Max	Siegl			Department of Pediatrics, University Hospital Wuerzburg	Würzburg, Germany	Field activities	
Fumi	Sugihara		MD	Department of Pediatrics, University Hospital Wuerzburg	Würzburg, Germany	Field activities	
Anne	Thieme		MD	Department of Pediatrics, University Hospital Wuerzburg	Würzburg, Germany	Field activities	
Gvantsa	Uturgaidze		MD	Department of Pediatrics, University Hospital Wuerzburg	Würzburg, Germany	Field activities	
Brigitte	Wehner			Department of Pediatrics, University Hospital Wuerzburg	Würzburg, Germany	Field activities	
