# Supplementary material for: Feasibility of SARS-CoV-2 Surveillance Testing Among Children and Childcare Workers at German Day Care Centers: A Nonrandomized Controlled Trial
Source: JAMA Netw Open. 2022 Jan 4;5(1):e2142057. doi: 10.1001/jamanetworkopen.2021.42057 (PMC8728621; doi:10.1001/jamanetworkopen.2021.42057)
Supplement: Supplement 4. — Data Sharing Statement [file jamanetwopen-e2142057-s004.pdf]

Data Sharing Statement

Forster. Feasibility of SARS-CoV-2 Surveillance Testing Among Children and Childcare Workers at German Day Care Centers. *JAMA Netw Open*. Published January 04, 2022. doi:10.1001/jamanetworkopen.2021.42057

Data
Data available: Yes
Data types: Deidentified participant data
How to access data: okurzai@hygiene.uni-wuerzburg.de (corresponding author)
When available: With publication

Supporting Documents
Document types: None

Additional Information
Who can access the data: Researchers whose proposed use of the data has been approved
Types of analyses: Research in the context infectious diseases spreading in child day care
Mechanisms of data availability: After approval of a proposal by the corresponding author
